# Urinary extracellular vesicles carry multiple activators and regulators of coagulation

**DOI:** 10.3389/fcell.2022.967482

**Published:** 2022-09-07

**Authors:** Mayank Saraswat, Beata Przybyla, Sakari Joenvaara, Tiialotta Tohmola, Tomas Strandin, Maija Puhka, Annukka Jouppila, Riitta Lassila, Risto Renkonen

**Affiliations:** ^1^ Transplantation Laboratory, University of Helsinki, Helsinki, Finland; ^2^ Coagulation Unit, Helsinki University Central Hospital, Helsinki, Finland; ^3^ HUSLAB, Helsinki University Hospital, Helsinki, Finland; ^4^ Department of Biosciences, University of Helsinki, Helsinki, Finland; ^5^ Department of Virology, Medicum, Univeristy of Helsinki, Helsinki, Finland; ^6^ EV Core and Institute for Molecular Medicine Finland, Helsinki, Finland; ^7^ Helsinki University Hospital Research Institute, Helsinki, Finland; ^8^ Research Program Unit in Systems Oncology, Coagulation Disorders Unit, Hematology and Cancer Center, Helsinki University Central Hospital, University of Helsinki, Helsinki, Finland

**Keywords:** urinary exosomes, blood coagulation, extracellular vesicles, hemostasis, urine

## Abstract

Cells shape their extracellular milieu by secreting intracellular products into the environment including extracellular vesicles which are lipid-bilayer limited membrane particles. These vesicles carry out a range of functions, including regulation of coagulation, via multiple contributor mechanisms. Urinary extracellular vesicles are secreted by various cells, lining the urinary space, including the nephron and bladder. They are known to have procoagulant properties, however, the details of this function, beyond tissue factor are not well known. The aim of the study was to access the role of urinary extracellular vesicles in impacting coagulation upon supplementation to plasma. This could indicate their physiological function upon kidney injury or pathology. Supplementation to standard human plasma and plasmas deficient in various coagulation factors was used for this purpose, and calibrated automated thrombogram (CAT^®^) was the major technique applied. We found that these vesicles contain multiple coagulation-related factors, and their lipid composition affects coagulation activities of plasma upon supplementation. Remarkably, these vesicles can restore thrombin generation in FVII, FVIII, FIX and FXI -deficient plasmas. This study explores the multiple roles of urinary extracellular vesicles in coagulation in *in vitro* blood coagulation and implies their importance in its regulation by several mechanisms.

## Introduction

Cells condition their environment by secreting the intracellular products into the extracellular milieu. These products act in an autocrine or paracrine manner to provide conditions and regulation for cell growth and maintenance. Extracellular vesicles (EVs; including exosomes and microvesicles), function in intercellular exchange and cargo of signals and informational contents (proteins, RNAs), regulation of angiogenesis, immune and coagulation mechanisms, among others (Berckmans et al., 2011; del Conde et al., 2005; Huber and Holvoet, 2015; Kapustin and Shanahan, 2016; Mause and Weber, 2010; Thery et al., 2009; Valadi et al., 2007).

A role of EVs in coagulation has been long recognized (Wolf, 1967; Siljander et al., 1996), and they were shown to support thrombin generation. Assembly of coagulation factors requires cell membranes, specifically negatively charged phospholipid surfaces, which are provided mostly by activated platelets and EVs circulating in blood (Aatonen et al., 2014). Urinary exosomes contain phosphatidylserine species (Skotland et al., 2017), which may support the assembly of coagulation factors. Intra-glomerular coagulation has been reported to play a role in development of glomerulonephritis (Vassalli and McCluskey, 1971; Hancock and Atkins, 1985) and hypercoagulability has been observed in association with chronic kidney disease (Adams et al., 2008; Salmela et al., 2015). However, the underlying mechanisms, in the context of kidneys, are largely unknown. Glomerular deposition of cross-linked fibrin in various human kidney diseases support the pathogenic role of the coagulation system (Takemura et al., 1987; Madhusudhan et al., 2016). Moreover, various coagulation factors are expressed in kidney (Madhusudhan et al., 2016; Oe et al., 2016) with uncertain functional roles. EVs can contribute to the procoagulant activity in biofluids (Kurosawa et al., 1984; Berckmans et al., 2011), with unclear details or mechanisms. EVs secreted by various cells lining the urinary tract are potential carriers of coagulation factors and functions and may importantly regulate intrarenal coagulation and subsequent pathogenesis.

We designed the current study to assess the thrombin generation (TG) by EVs in human plasma using calibrated automated thrombogram (CAT^®^). We further compared urinary exosomes with microvesicles for their hemostatic potential and characterized the presence and activity of several coagulation factors (F) in both vesicle species. We show that EVs enhance TG in plasma to varying degrees. Multiple coagulation factors, such as FII, VII, VIII, IX, X, XI, XIII, tissue factor (TF), fibrinogen and naturally occurring anticoagulants, such as tissue factor pathway inhibitor (TFPI), antithrombin and activated protein C (APC) are present in urinary EVs, many of them in their active form. The implication of coagulation factors, their regulation and the thrombotic potential of EVs appears important in *in vitro* blood coagulation.

## Materials and methods

### Aims, design and setting of the study

The aim of the study was to establish the effect of urinary extracellular vesicles on blood coagulation. This will indicate and reflect on their coagulation related functions in the kidney. The study was designed to reveal which coagulation factors are important in procoagulant and anticoagulant functions of urinary EVs purified from healthy individuals. Calibrated Automated Thrombogram (CAT^®^) was used to establish the influence of EVs on thrombin generation capacity of standard and various factor deficient plasmas. Various other biochemical assays were also utilized as detailed below.

### Urine collection, ethics, consent and permissions

First morning urine samples were collected from nine healthy volunteers, unless otherwise specified, ranging from 24 to 57 years of age (Five males and four females). Samples were tested by Combur 10 Test®D dipstick (Roche Diagnostics; Mannheim, Germany) and values were within normal range or negative. Coordinating Ethics Committee, Hospital District of Helsinki and Uusimaa, Finland, approved the study with following number: 114/13/03/00/16. Written informed consent was obtained from the healthy volunteers. All methodology complies with the relevant guidelines and regulations.

### Extracellular vesicles purification

The EVs (exosomes and microvesicles) were purified as previously described (Musante et al., 2012; Musante et al., 2013; Saraswat et al., 2015). Briefly, urine samples were centrifuged at 2,000 g for 20 min (Eppendorf 5810R, rotor F34-6-38, Hamburg, Germany). The resulting supernatant was filtered and concentrated at the same time using Amicon ultra 15 (MWCO 100 kDa, Merck, Darmstadt, Germany) to 20 times less volume. The resulting concentrate was centrifuged at 18,000 g for 30 min (Eppendorf 5810R, rotor F34-6-38). The pellet was resuspended in MQ water or PBS depending on the downstream application or assay. This pellet was designated microvesicle pellet or P18. The supernatant from this step was further centrifuged at 200,000 g for 2 h (Beckman centrifuge, rotor 70.1Ti, fixed angle). The pellet from this step was termed as exosome pellet or P200. A few details to note are that after centrifugation at 2,000 g to remove bigger debris, cells etc., the urine was concentrated using a 100 kDa filter, which would remove all soluble proteins smaller than 100 kDa. Filter was washed two times by adding ten time the volume of water and every pellet that was washed with PBS without disturbing the pellet, and inverted tubes cleaned with no-lint paper before reconstitution of the pellet. This left no residual supernatant or smaller soluble proteins. P18 and P200 were always reconstituted and used at the total protein concentration of 50 μg/ml in all assays, unless otherwise stated.

### Calibrated automated thrombogram

Total thrombin generation was assessed with CAT^®^ (Labscan, Thermo Fisher, Helsinki, Finland) (Hemker et al., 2002) in 3.2% citrated standard human plasma (Siemens Healthcare, Marburg, Germany). More details are provided in Supplementary Methods.

### Measurement of various coagulation factors

All factor levels were analyzed with BCS XP analyzer (Siemens, Erlangen, Germany) with the reagents from Siemens. Reference values used for various factors were as follows: FII: 68–144 International unit per deciliter (IU/dL), FV: 65–140 IU/dL, FVII: 76–170 IU/dL, FVIII: 52–148 IU/dL, FIX: 67–135 IU/dL, FX 76–146 IU/dL, FXI 60–120 IU/dL, FXII 60–150 IU/dL.

### Activity assays

APC activity was measured in P18 (microvesicles) and P200 (exosomes) as instructed by the manufacturer (SEA738Hu, Cloud-Clone Corp. TX, United States ). Factor Xa activity was measured in P18 and P200 with and without adding PPP reagent Low^®^ and PRP reagent^®^ (without addition of phospholipids) according to manufacturer’s instructions (Abcam, Cambridge, Great Britain).

### Trypsin digestion and proteomic analysis

Trypsin digestion and proteomics analysis has been described previously (Saraswat et al., 2019; Joenväärä et al., 2022), with all modifications noted here. Mass spectrometry analysis was performed as described previously (Saraswat et al., 2016). Further detailed methods are available in Supplementary Material.

### Transmission electron microscopy

Samples were prepared for EM and imaged as described (Puhka et al., 2017). Further details are furnished in Supplementary Material.

Further detailed methods for several procedures can be found in Supplementary Material.

## Results

### Proteomic analysis differentiates exosomes and microvesicles purified from healthy human urine

**FIGURE 1:**
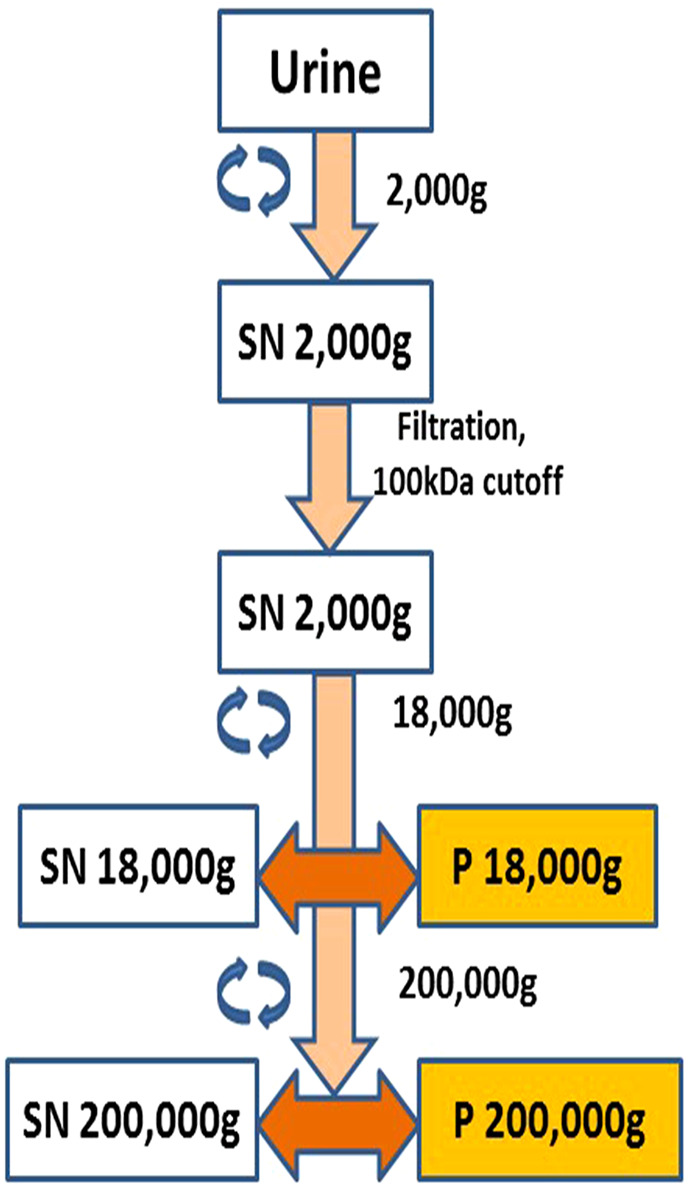
Schematic diagram showing extracellular vesicle (EV) purification and isolation. Pellet 18,000 g (P18) was designated the microvesicles pellet and Pellet 200,000 g (P200) was designated the exosomes pellet.

Previously published protocols were used for isolation of microvesicles (P18) and exosomes (P200) from healthy volunteer’s urine (Musante et al., 2012; Musante et al., 2013; Saraswat et al., 2015) (Figure 1). P18 and P200 were analyzed by LC-MSE, and 586 proteins were identified with two or more unique peptides (Supplementary Table S1). Three proteins were uniquely detected in P200, while all the others were common to both P18 and P200. These three proteins were Gamma-interferon-inducible protein IP-30, transmembrane seven superfamily member three and lysosomal membrane protein 2. Protein abundances were markedly different between P18 and P200. EV marker proteins (CD81, CD9, Alix, Tumor susceptibility protein 101 (TSG101) was more abundant in P200 than P18. Using the multivariate data analysis technique orthogonal projections to latent structures-discriminant analysis (OPLS-DA), it was possible to find the proteins, which distinctly classified the two fractions (Supplementary Figure S1 and Supplementary Table S2).

CD63, Alix and TSG101 were verified by western blot, and their profiles differed between the P18 and P200 (Supplementary Figure S2). Representative transmission electron microscopy profiles of typical vesicles associated with P18 and P200 are shown in Supplementary Figure S3.

### Various active coagulation factors are present in EVs and shorten activated partial thromboplastin time in plasma

FII, FV, FVII, FVIII, FIX, FX, FXI, FXII, and FXIII were measured in P18 (at 1 mg/ml total protein) and P200 (at 1 mg/ml total protein). FII, FV, FX, and FXIII were below the detection limit. The levels varied for different factors; FVII (P18: 7.5 IU/ml and P200: 32 IU/ml), FVIII (P18: 10 IU/ml and P200: 5 IU/ml), FIX (P18: 1.25 IU/ml and P200: 2 IU/ml), FXI (P18: 15 IU/ml and P200: 18 IU/ml) and FXII (P18: 10 IU/ml and P200: 10 IU/ml). Subsequently, we purified P18 and P200 from eight different individuals, and they were screened Calibrated Automated Thrombogram (CAT^®^), activity assay and western blotting. Thrombin generation was measured by CAT upon supplementation to standard human plasma and plasma deificient in various coagulation factors at defined total protein concentration of EVs (50 μg/ml total protein for both P18 and P200).

### Urinary EVs enhance thrombin generation in plasma and restore it in lipid-devoid plasma

**FIGURE 2:**
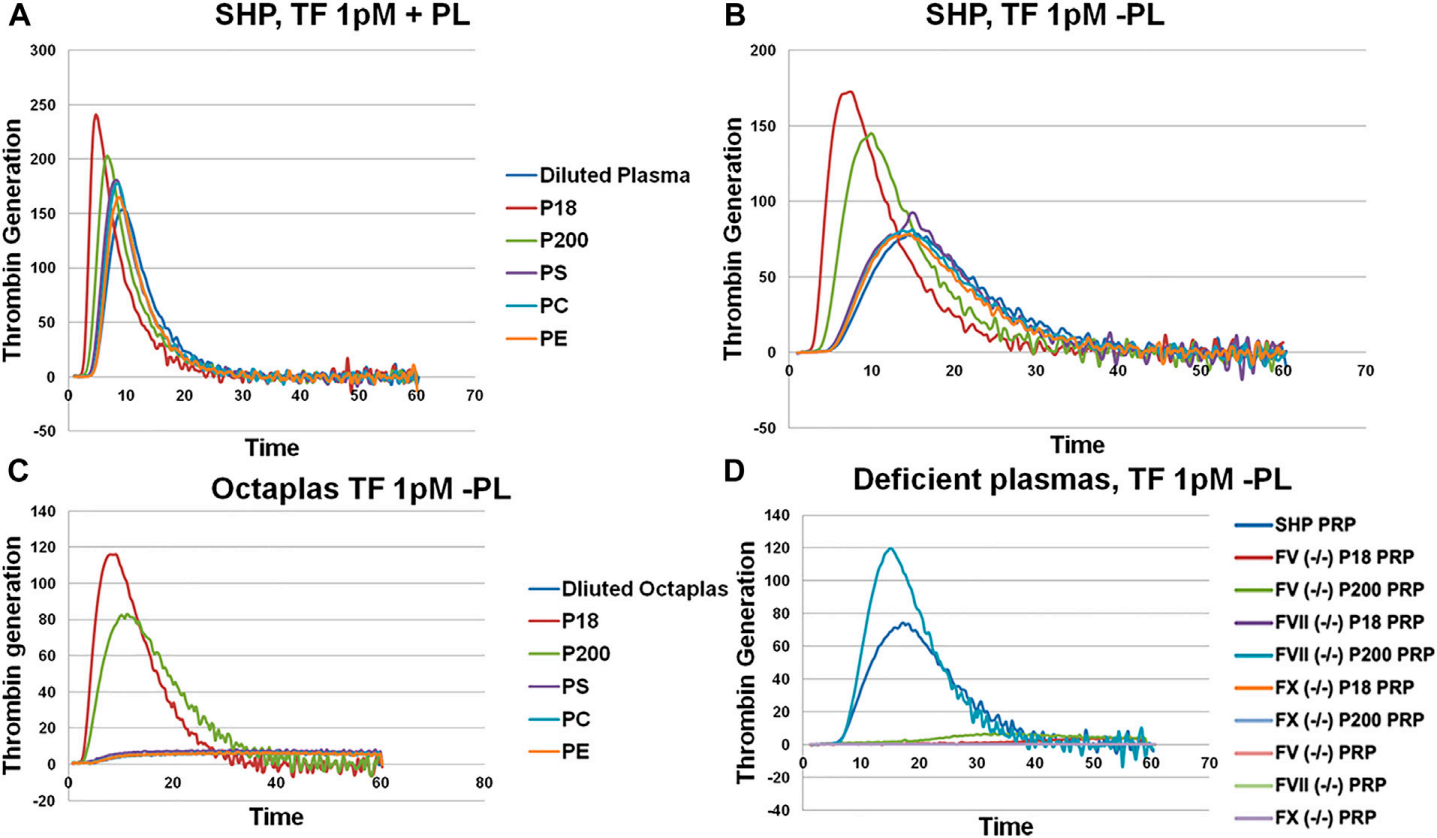
Thrombin generation measured by Calibrated Automated Thrombogram (CAT®). EVs (P18 (microvesicles) and P200 (exosomes)) were supplemented into plasma, and TG was measured after challenging the plasma with PPPLow **(A)** (1pM Tissue Factor (TF) + Phospholipids (PL)) or PRP reagents **(B)** (1pM Tissue Factor (TF) without PLs). Diluted plasma (with PBS at same volume as EVs) was used a control. A fixed concentration (25 µg/ml) of PLs phosphatidylserine (PS), phosphatidylcholine (PC) and phosphatidylethanolamine (PE) was added to plasma instead of EVs in separate wells, and TG was measured as additional controls (A and B). TG was also measured in Octaplas (SDP) by diluting with either PBS or EVs (P18 and P200) or PLs (PS, PC, PE) and subsequently challenged with the PRP reagent **(C)**. Specific plasmas, deficient in various coagulation factors (FV, FVII and FX) were also challenged with the PRP reagent with or without supplementation with P18 or P200, and TG was measured **(D)**. X-axis represents time and Y-axis TG in nM.

Next, we assessed thrombin generation to clarify the global impact of EV on coagulation using Calibrated Automated Thrombogram (CAT^®^). Low TF concentration (1 pM) was used to trigger TG, wherein the contact pathway and the role of TFPI are also involved. When the P18 and P200 were added (both at 50 μg/ml protein) to standard human plasma, they enhanced the thrombin generation compared with standard human plasma diluted with the same volume of PBS instead of P18 or P200 (Figure 2), supporting that the vesicles were procoagulant. Next, to explore the role of EV’s phospholipids (PL), both the PPPLow reagent (1 pM TF with exogenous PLs) and the PRP reagent (1 pM TF without exogenous PLs) were used (Figures 2A,B). The PPPLow reagent increased thrombin generation upon supplementation with EVs more than PRP reagent. It confirms that phospholipids are an integral part of thrombin generation in plasma and that P18 and P200 can supply the required lipid species.

P18 shortened lag time, and thrombin peak inclined clearly more by P200 (in both PPPLow and PRP reagent challenge). Also, both P18 and P200 enhanced thrombin generation capacity in comparison with the plasma control (plasma diluted by same amount of PBS instead of P18 or P200). Endogenous thrombin potential was significantly enhanced compared with control, but similarly with P18 and P200. Addition of phosphatidylserine, phosphatidylcholine and phosphatidylethanolamine to plasma failed to further enhance the thrombin generation more than the plasma baseline (with the PRP reagent). It suggests that three-dimensionally arranged lipids (such as those in EVs) are better at supporting thrombin generation that merely adding purified lipids.

Solvent/detergent -treated plasma (contains no PL, Octaplas, Octapharma, Lachen, Switzerland) (Pitkänen et al., 2015) was used next to capture the effect of PL composition on TG upon plasma supplementation with EVs. Solvent/detergent -treated plasma challenged with the PRP reagent, did not support thrombin generation. However, addition of either P18 or P200 restored thrombin generation (Figure 2C). Again, P18 exceeded the activity of P200 in the thrombin generation variables, such as lag time and thrombin peak. It indicates the involvement of lipid species of EVs in supporting thrombin generation.

### Urinary EVs efficiently rescue various coagulation factors-deficient plasmas

To establish those coagulation factors, which enhanced thrombin generation in CAT^®^ in response to low TF concentrations, various factor-deficient plasmas were studied in the absence or presence of pre-incubated P18 and P200, without exogenous PL. Specifically, P200 rescued thrombin generation in FVII deficient plasma, whereas pre-incubation with P18 failed (Figure 2D). In contrast, FII (prothrombin), FV and FX -deficient plasmas were not rescued by adding either P200 or P18.

**FIGURE 3:**
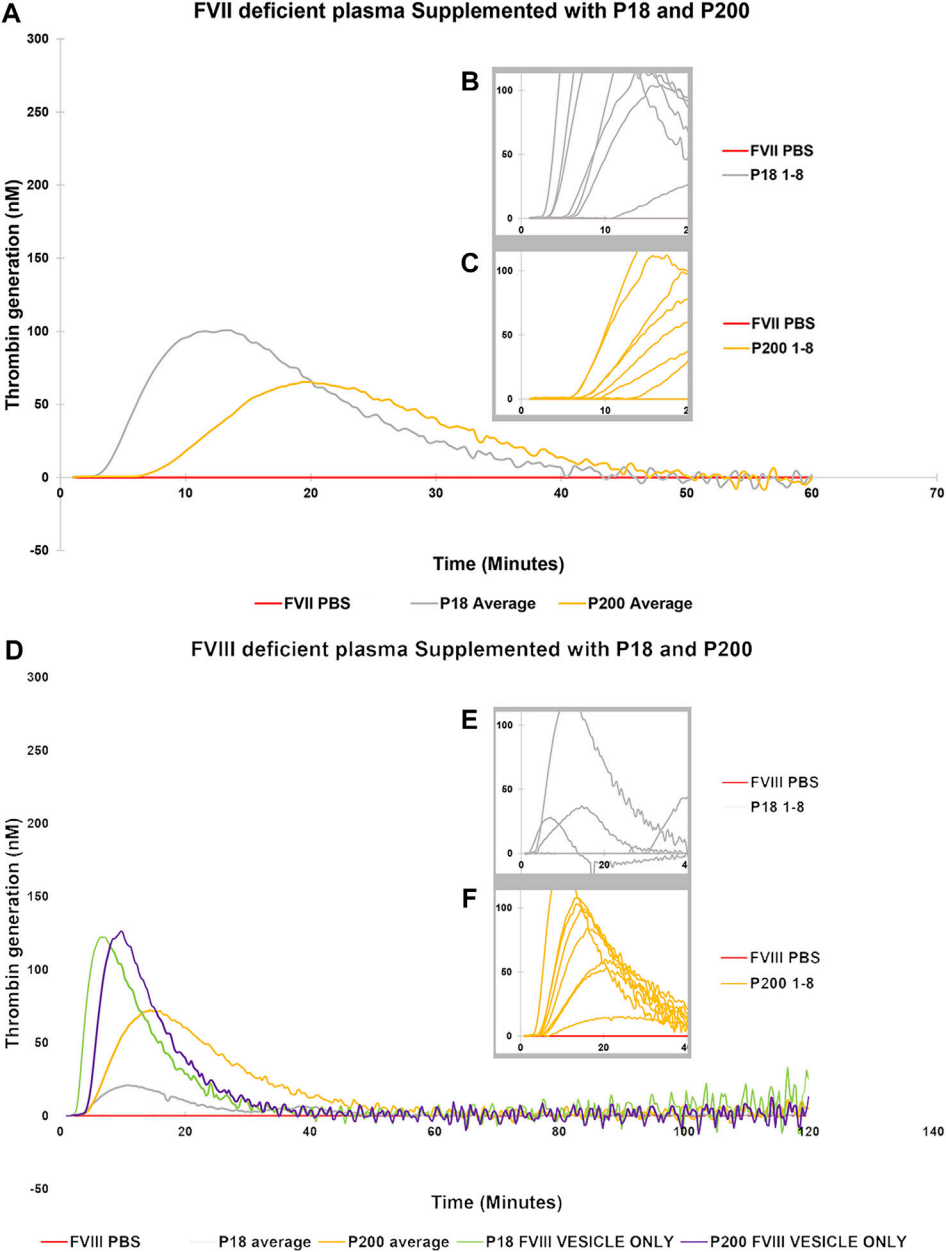
Thrombin Generation measured by Calibrated Automated Thrombogram (CAT®) is rescued in FVII and FVIII deficient plasmas by supplementation of urinary extracellular vesicles. EVs (P18 (Grey line, microvesicles) and P200 (Orange line, exosomes) were supplemented into FVII deficient plasma **(A–C)**, and TG was measured after challenging the plasma with the PRP reagent (1pM TF without PLs). Similarly, P18 and P200 were supplemented into FVIII deficient plasmas **(D–F)**, and TG was measured after challenging the plasma with PRP reagent. Diluted plasma (with PBS at same volume as EVs) was used a control (Red line). In A and B the averages at every time point for 8 measurements for both P18 and 8 P200 are shown. These 8 separate measurements are EVs (P18 and P200) purified from 8 individuals. The average is depicted to enhance clarity. Inset diagrams (B and C for FVII and E and F for FVIII) show a cut portion of the original diagram with 8 individuals’ measurements to highlight the differences in lag time (start of the TG) between the individuals (not evident in A and D). X-axis represents time and Y-axis TG in nM. Individual curves and statistics are described in Supplementary Figures S10, S11.

**FIGURE 4:**
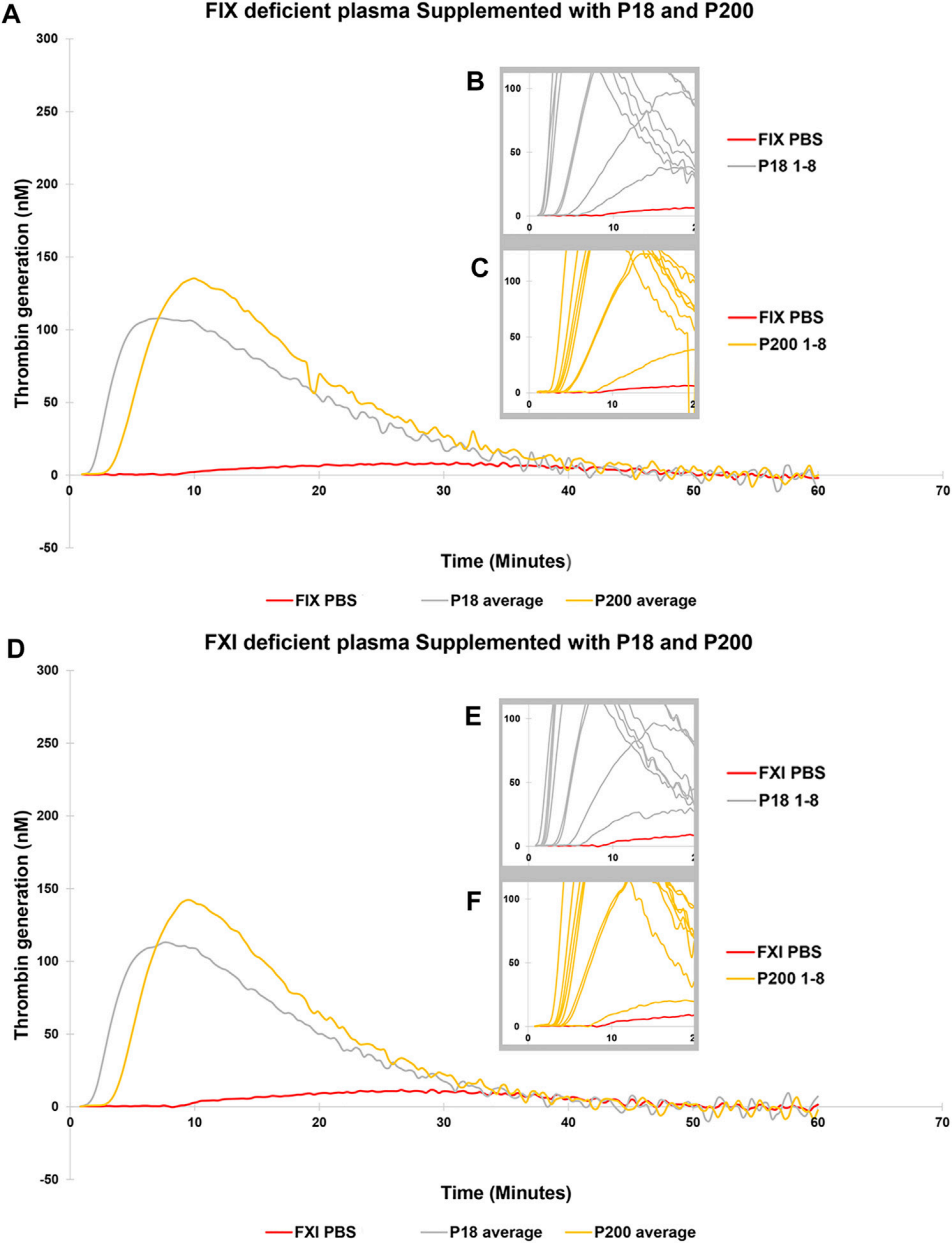
Thrombin Generation measured by Calibrated Automated Thrombogram (CAT®) is rescued in FIX and FXI deficient plasmas by adding urinary extracellular vesicles. EVs (P18 (Grey line, microvesicles) and P200 (Orange line, exosomes) were supplemented into FIX deficient plasma **(A–C)**, and TG was measured after challenging the plasma with the PRP reagent (1pM TF without PLs). Similarly, P18 and P200 were supplemented into FXI deficient plasma **(D–F)**, and TG was measured after challenging the plasma with PRP reagent. Diluted plasma (with PBS at same volume as EVs) was used a control (Red line). In A and B the averages at every time point for 8 samples for both P18 and P200 are shown. The average is depicted to enhance clarity. Inset diagrams (B and C for FVII and E and F for FVIII) show a cut portion of the original diagram with 8 individuals’ measurements to highlight the differences in lag time (start of the TG) between individuals (not evident in A and D). X-axis represents time and Y-axis TG in nM. Individual curves and statistics are described in Supplementary Figures S12, S13.

Next, to establish the thrombin generation support and variability by EVs among more donors, EVs (both P18 and P200) were purified from eight additional individuals and tested in the CAT^®^ assay using standard human plasma (Supplementary Figure S5), plasmas deficient of FVII (Figures 3A–C), FVIII (Figures 3D–F), FIX (Figures 4A–C) and FXI (Figures 4D–F). The EVs rescued all the tested deficient plasmas and generated thrombin upon supplementation at the same protein concentration. For FVII deficient plasma, in contrast to low TF without phospholipids (Figure 2D), PRP reagent (TF with minimal phospholipids) allowed rescue of thrombin generation by both P18 and P200. It suggests minimal amounts of phospholipids are required as a trigger for thrombin generation by P18. Further, the vesicles did not need any external trigger, such as addition of TF, to generate thrombin in FVIII, FIX and FXI deficient plasmas (Figure 3 and Supplementary Figure S6), since they spontaneously supported thrombin generation. The presence of FVIII was also evident in western blot but detected only in P200 (full length in non-reducing conditions, Supplementary Figure S7).

Vesicles alone, in the absence of plasma, when incubated with either the PPPLow or the PRP reagent did not generate any thrombin (data not shown). P18 and P200 were both positive for prothrombin and TF in western blot, whereas thrombin was absent (Supplementary Figure S8). FV was present in the western blot in both vesicles in a degraded form (Supplementary Figure S8). To exclude FV being a limiting factor for thrombin generation, exogenous purified FV and calcium ions were added to the vesicles, but thrombin did not form (data not shown), suggesting a regulatory element present in the EVs.

### FXa is present in urinary EVs and augmented by external tissue factor

**FIGURE 5:**
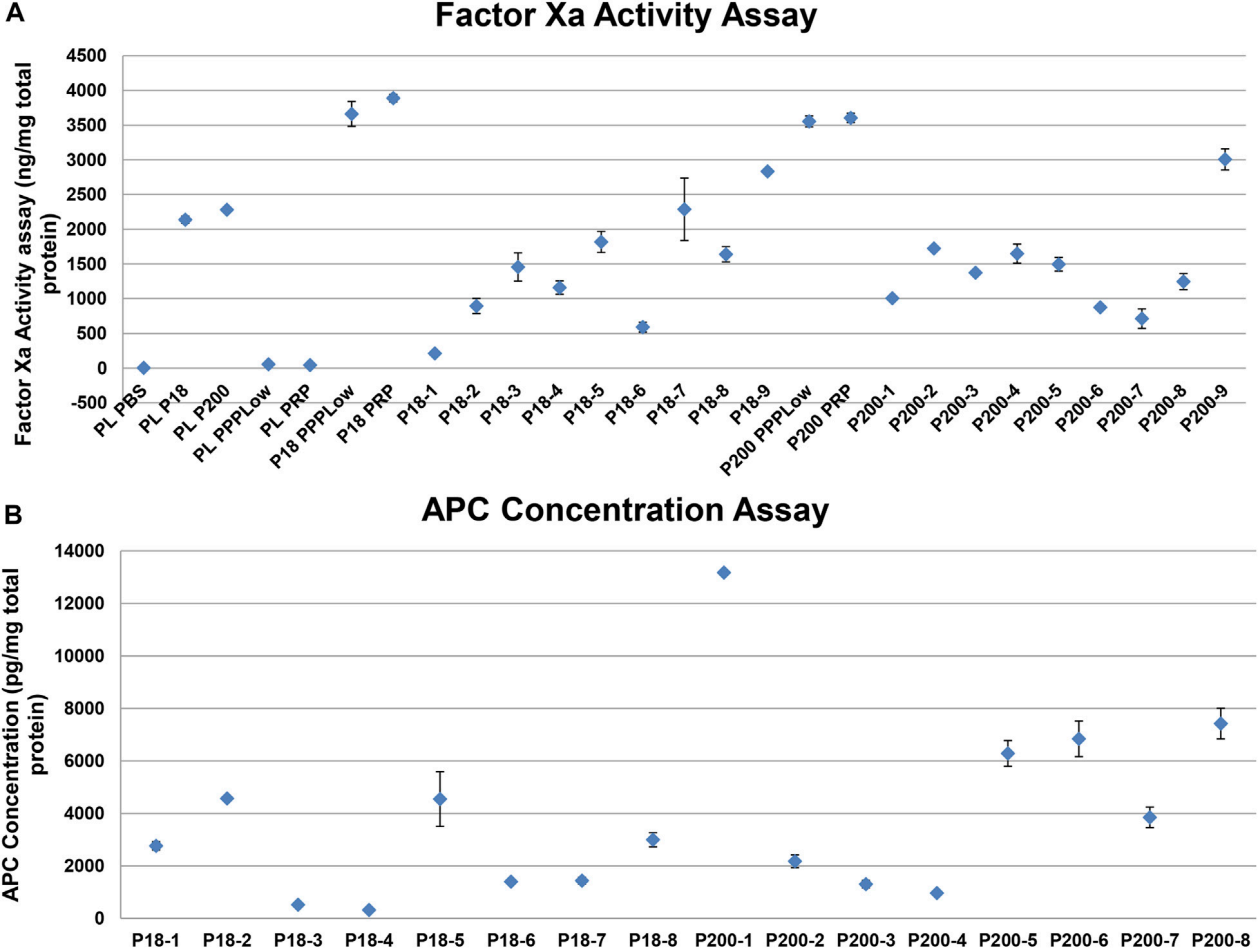
Activity measurement of factor Xa and activated protein C shows variable but definite activity among individual samples. **(A)** Dot plot showing Factor Xa activity (with SD of triplicates) in various samples. PLPBS (Plasma diluted with PBS to same volume as EVs). PLP18 (Plasma diluted with P18 5:4 at P18 concentration of 50 µg/m). PLP200 (Plasma diluted with P200, 5:4 at P200 concentration of 50 µg/ml). PLPPPLow and PLPRP (diluted plasma with the PPPLow reagent and the PRP reagent at 4:1). P18 (Microvesicles without any addition), P200 (Exosomes without any addition), P18PPPLow and P18PRP (P18 supplemented with the PPPLow and the PRP reagent, respectively at 4:1). P200PPPLow and P200PRP (P200 supplemented with the PPPLow and the PRP reagent, respectively at 4:1). **(B)** Activated protein C (APC) activity in P18 (Microvesicles) per 1mg of total protein from 8 donors (P18-1 to P18-8), and the APC activity in P200 (Exosomes) per 1mg of total protein from 8 donors (P200-1 to P200-8), using the fluorescent substrate-based assay.

FXa as measured with a fluorescence-based activity assay in standard human plasma, in the absence and presence of P18 or P200, and in vesicles alone. standard human plasma alone did not express any activity but adding either P18 or P200 resulted in FXa activity in standard human plasma (Figure 5). standard human plasma showed better FXa activity at P200 supplementation compared to P18, at the same total protein concentration. Stimulation by 1 pM TF and PLs, or 1 pM TF alone increased the FXa activity to the same extent, which exceeded many-fold that of P18 or P200 without stimulation. FX was positive in P200 by western blot, while absent in P18 (Supplementary Figure S9), while FXa was found in both fractions by activity assay as described above (Figure 5).

### Natural anticoagulants are present in urinary EVs

Despite the presence of both prothrombin and FXa in EVs, these alone failed to generate any thrombin. Therefore, we anticipated the presence of endogenous inhibitors of the prothrombinase complex, including TFPI and AT. Both these proteins were observed in both fractions, compatibly with the TG results, more visibly in P200 (P18 and P200, Supplementary Figure S7). The APC displayed generally stronger activity in P200 than in P18 among the eight individual samples (Figure 5). In accordance with the APC, FV seemed degraded in western blots of both types of vesicles (Supplementary Figure S8).

## Discussion

Urine has been long known to possess procoagulant properties. Urine procoagulant was reported unchanged in hemophilia patients suggesting it does not rely on the activation of the intrinsic pathway (Matsumura and von Kaulla, 1968). This procoagulant activity was later associated with vesicles having the size of 100–1,000 nm (Wiggins et al., 1986). The origin of urinary TF is likely tubular cells (Lwaleed et al., 2007), supported by clinical and biochemical evidence (Lwaleed et al., 1999). In the literature the effects of urine on coagulation system are largely attributed to vesicle-associated TF (Kurosawa et al., 1984). However, beyond TF human urine carries also anticoagulant properties (Takahashi et al., 1995). Platelet exosomes have been postulated to possess both procoagulant and anticoagulant roles (Srikanthan et al., 2014). Clearly, multiple factors participate and interact to contribute to the hemostatic and/or procoagulant potential of urinary vesicles. Our study suggests a new avenue to appreciate urinary EV’s potential involvement in intrarenal TG and overall coagulation in health and disease.

This study establishes the physiological presence of a multitude of coagulation factors both in urinary exosomes and microvesicles derived from healthy subjects. The TG by CAT^®^ assays and kit-based activity assays (for activity of various factors) were prominent in the nine subjects tested, however, inter-individual variability was present. Western blotting similarly displayed variable results in detecting the presence or absence of various factors. Further, we did not break open these vesicles and intact vesicles were able to support thrombin generation in standard human plasma an several plasma deficient in specific coagulation factors, suggesting these factors are present on EV surface.

Various plasmas deficient in coagulation factors were tested and remarkably, urinary EVs rescued the thrombin generation when supplemented in FVII, FVIII, FIX, and FXI deficient plasmas. To elaborate on the mechanisms of coagulation and its regulation more sensitive test, i.e., calibrated automated thrombogram (CAT^®^) was employed to assess the thrombin generation potential of the exosomes and microvesicles. It relied on both 1) the low TF concentration, allowing the exploration of natural anticoagulants, and 2) the absence of exogeneous PLs in the assay, relying on the potential effects of vesicle PLs. Upon addition of exosomes and microvesicles (equal protein quantity) to standard human plasma, lag time decreased, and peak thrombin and endogenous thrombin potential increased (challenging with both the PPPLow and PRP reagent). These effects were more pronounced with P18 than P200, which exceeded these parameters observed in only standard human plasma, emphasizing the role of vesicle membrane PLs. Peak height of thrombin benefited from the PPPLow reagent more than PRP reagent, the latter relying only on the PLs of the exosomes or vesicles. Thrombin peak has been suggested to reflect the concentrations of prothrombin, FVIII, FIX, FX, FXI, fibrinogen and fibrin monomers (Machlus et al., 2009; Pitkänen et al., 2017). Accordingly, urinary EVs were able to rescue thrombin generation in FVIII, FIX, and FXI deficient plasmas.

Both P18 and P200, similarly increased endogenous thrombin potential in standard human plasma, having similar factor sensitivity as peak height. However, our western blot and activity assays, CAT^®^ and proteomic analysis suggested that these factors (prothrombin, FVIII, FX, and fibrinogen) were more enriched in P200 than P18. Therefore, we suggest that P18-based thrombin generation enhancement is due to its appropriate lipid species versus P200. However, further investigation is needed. This suggestion was also supported by solvent/detergent-delipidated plasma, which being unable to generate thrombin by itself, could be rescued more efficiently by P18 than by P200. However, lipids alone do not enhance thrombin generation, as added pure PLs failed to improve the thrombin generation in standard human plasma in contrast to P18 and P200. Importantly, the three-dimensional arrangement of lipid species may not be simulated by the dissolved lipids, which are added to the plasma. In platelet-poor plasma, the main determinant for thrombin generation is the exogenous procoagulant lipid species of phosphatidylserine (PS). However, phosphatidylethanolamine is also involved in thrombin generation (Falls et al., 2000; von dem Borne et al., 1997; Tavoosi et al., 2013). In solvent/detergent treated plasma, when the PPPLow reagent (PL and 1 pM TF) was used, the difference between P18 and P200 attenuated. However, intriguingly upon adding the PRP reagent (1 pM TF without PL) the difference exaggerated. Our results imply that P18 contain the appropriate lipid quantity and species to support thrombin generation better than P200.

Literature reveals that FVII is crucial for the procoagulant behavior of vesicles (Kurosawa et al., 1984). Therefore, we tested the effect of P18 and P200 addition on thrombin generation in FVII deficient plasma. We observed the rescue effect by both P18 and P200 on thrombin generation in the FVII deficient plasma. This occurred despite that both P18 and P200 lacked FVII bands in the western blots. This could be explained by a non-working antibody or a minimal amount of FVII. Our observation of EV rescue of FVII deficient plasma is supported by the published reports (Kurosawa et al., 1984). Since high TF concentration can bypass the FVII requirement for thrombin generation (O'Brien et al., 1988), we targeted the low TF concentration (1 pM), which discerns and highlights the effects of natural coagulation components in the EVs. The vesicles also rescued the FVIII, FIX and FXI deficient plasmas in an individual-dependent manner. These vesicles, both P18 (microvesicles) and P200 (exosomes), when purified from healthy individual’s urine seem to act as hemostatic agents on various factor-deficient plasmas and restore their defective thrombin generation.

Despite containing most components of intrinsic and extrinsic coagulation pathway, the vesicles alone (without plasma) did not generate thrombin even upon the addition of 1 pM TF with and without PL (data not shown). This prompted us to study the regulatory anticoagulant system, potentially present in these vesicles. FXa activity hydrolyzed the fluorescent substrate, but, it could also be in an inhibited state, while the small peptide substrate can still access the active site and be converted to produce the signal. P200 contained more AT and TFPI than P18. This physiological mechanism could suppress the unnecessary thrombin generation in healthy kidneys avoiding any damage [17]. The co-presence of TFPI and extrinsic coagulation pathway components in these vesicles contribute to the net effect of thrombin generation when supplemented to plasma. Moreover, P18 and P200 contained APC, as judged by the ELISA and accorded with the degraded FV in these vesicles. However, FVIII was intact and active, despite APC. This could be explained by likely absence of protein S (not tested) or presence of protein C inhibitor (PCI, not tested) in the vesicles. As the APC kit is an ELISA the APC-PCI complex may be detected. Additionally, while vesicle FVIII is present in concentrations like plasma, but APC is present at 1,000-fold smaller amounts and combined with limited cofactors (Protein S and FV), FVIII would not be degraded. These conjectures are subject to further ongoing investigations.

Despite the lack of indigenous/endogenous thrombin generation, upon mixing with plasma EVs enhance thrombin generation, likely mediating the hemostatic balance in the kidney. Overall, microvesicles and exosomes are distinctly composed by coagulation proteins and show different capacity to generate thrombin in plasma environment. Different routes of uptake and secretion for these two different types of vesicles in the kidney may occur. We could not mechanistically establish if thrombin generation supported by the urinary EVs is mediated by intrinsic or extrinsic coagulation pathway and this warrants future studies. Additionally, urinary extracellular vesicles depicted several roles in *in vitro* coagulation processes and regulatory mechanisms. The *in vivo* role of EVs should be further investigated, and targeted studies of pathological mechanisms of fibrin deposition in kidneys are warranted.

## Data Availability

The original contributions presented in the study are included in the article/Supplementary Material, further inquiries can be directed to the corresponding author.

## References

[B1] Aatonen M. T. Ohman T. Nyman T. A. Laitinen S. Grönholm M. Siljander P. R. M. (2014). Isolation and characterization of platelet-derived extracellular vesicles. J. Extracell. Vesicles 3, 24692. 3402/Jev.V3.24692. 10.3402/jev.v3.24692 PMC4125723 25147646

[B2] Adams M. J. Irish A. B. Watts G. F. Oostryck R. Dogra G. K. (2008). Hypercoagulability in chronic kidney disease is associated with coagulation activation but not endothelial function. Thromb. Res. 123, 374–380. 10.1016/j.thromres.2008.03.024 18486198

[B3] Berckmans R. J. Sturk A. Van Tienen L. M. Schaap M. C. L. Nieuwland R. (2011). Cell-derived vesicles exposing coagulant tissue factor in saliva. Blood 117, 3172–3180. 10.1182/blood-2010-06-290460 21248061

[B4] Del Conde I. Shrimpton C. N. Thiagarajan P. López J. A. (2005). Tissue-factor–bearing microvesicles arise from lipid rafts and fuse with activated platelets to initiate coagulation. Blood 106, 1604–1611. 10.1182/blood-2004-03-1095 15741221

[B5] Falls L. A. Furie B. Furie B. C. (2000). Role of phosphatidylethanolamine in assembly and function of the factor Ixa−Factor viiia complex on membrane surfaces. Biochemistry 39, 13216–13222. 10.1021/bi0009789 11052674

[B6] Hancock W. Atkins R. (1985). Activation of coagulation pathways and fibrin deposition in human glomerulonephritis. Semin. Nephrol. 5, 69–77. 3843783

[B7] Hemker H. C. Giesen P. Aldieri R. Regnault V. De Smed E. Wagenvoord R. (2002). The calibrated automated thrombogram (cat): A universal routine test for hyper- and hypocoagulability. Pathophysiol. Haemost. Thromb. 32, 249–253. 10.1159/000073575 13679651

[B8] Huber H. J. Holvoet P. (2015). Exosomes: Emerging roles in communication between blood cells and vascular tissues during atherosclerosis. Curr. Opin. Lipidol. 26, 412–419. 10.1097/MOL.0000000000000214 26309202 PMC4564019

[B9] Joenväärä S. Holm M. Saraswat M. Agarwal R. Tohmola T. Kajantie E. (2022). Quantitative urine proteomics in pregnant women for the identification of predictive biomarkers for preeclampsia. Transl. Med. Commun. 7, 1. 10.1186/s41231-022-00108-6

[B10] Kapustin A. N. Shanahan C. M. (2016). Emerging roles for vascular smooth muscle cell exosomes in calcification and coagulation. J. Physiol. 594, 2905–2914. 10.1113/JP271340 26864864 PMC4887700

[B11] Kurosawa S.-I. Matsuda M. Aoki N. (1984). Urinary procoagulant behaves as tissue factor by promoting factor viia-catalyzed activation of factor X. Thromb. Res. 33, 595–606. 10.1016/0049-3848(84)90114-2 6609450

[B12] Lwaleed B. A. Bass P. S. Francis J. L. Chisholm M. (1999). Functional and structural properties of urinary tissue factor. Nephrol. Dial. Transpl. 14, 588–596. 10.1093/ndt/14.3.588 10193804

[B13] Lwaleed B. A. Vayro S. Racusen L. C. Cooper A. J. (2007). Tissue factor expression by A human kidney proximal tubular cell line *in vitro*: A model relevant to urinary tissue factor secretion in disease? J. Clin. Pathol. 60, 762–767. 10.1136/jcp.2006.039636 17158639 PMC1995797

[B14] Machlus K. R. Colby E. A. Wu J. R. Koch G. G. Key N. S. Wolberg A. S. (2009). Effects of tissue factor, thrombomodulin and elevated clotting factor levels on thrombin generation in the calibrated automated thrombogram. Thromb. Haemost. 102, 936–944. 10.1160/TH09-03-0180 19888532 PMC2904819

[B15] Madhusudhan T. Kerlin B. A. Isermann B. (2016). The emerging role of coagulation proteases in kidney disease. Nat. Rev. Nephrol. 12, 94–109. 10.1038/nrneph.2015.177 26592189 PMC4933505

[B16] Matsumura T. Von Kaulla K. N. (1968). Procoagulant content of human urine in health and disease. Am. J. Clin. Pathol. 50, 198–210. 10.1093/ajcp/50.2.198 5673085

[B17] Mause S. F. Weber C. (2010). Protagonists of A novel communication network for intercellular information exchange. Microparticles 107, 1047–1057. 10.1161/CIRCRESAHA.110.226456 21030722

[B18] Musante L. Saraswat M. Duriez E. Byrne B. Ravidà A. Domon B. (2012). Biochemical and physical characterisation of urinary nanovesicles following chaps treatment. Plos One 7, E37279. 10.1371/journal.pone.0037279 22808001 PMC3395701

[B19] Musante L. Saraswat M. Ravidà A. Byrne B. Holthofer H. (2013). Recovery of urinary nanovesicles from ultracentrifugation supernatants. Nephrol. Dial. Transpl. 28, 1425–1433. 10.1093/ndt/gfs564 23258757

[B20] O'brien D. P. Giles A. R. Tate K. M. Vehar G. A. (1988). Factor viii-bypassing activity of bovine tissue factor using the canine hemophilic model. J. Clin. Invest. 82, 206–211. 10.1172/JCI113571 3134399 PMC303495

[B21] Oe Y. Hayashi S. Fushima T. Sato E. Kisu K. Sato H. (2016). Coagulation factor Xa and protease-activated receptor 2 as novel therapeutic targets for diabetic nephropathy. Arterioscler. Thromb. Vasc. Biol. 36, 1525–1533. 10.1161/ATVBAHA.116.307883 27283743

[B22] Pitkänen H. H. Jouppila A. Lemponen M. Ilmakunnas M. Ahonen J. Lassila R. (2017). Factor xiii deficiency enhances thrombin generation due to impaired fibrin polymerization — an effect corrected by factor xiii replacement. Thrombosis Res. 149, 56–61. 10.1016/j.thromres.2016.11.012 27902939

[B23] Pitkänen H. Jouppila A. Mowinckel M.-C. Lemponen M. Patiwael S. Sandset P. M. (2015). Enhanced thrombin generation and reduced intact protein S in processed solvent detergent plasma. Thromb. Res. 135, 167–174. 10.1016/j.thromres.2014.10.020 25466844

[B24] Puhka M. Nordberg M. E. Valkonen S. Rannikko A. Kallioniemi O. Siljander P. (2017). Keepex, A simple dilution protocol for improving extracellular vesicle yields from urine. Eur. J. Pharm. Sci. 98, 30–39. 10.1016/j.ejps.2016.10.021 27771514

[B25] Salmela A. Ekstrand A. Joutsi-Korhonen L. Räisänen-Sokolowski A. Lassila R. (2015). Activation of endothelium, coagulation and fibrinolysis is enhanced and associates with renal anti-neutrophil cytoplasmic antibody-associated vasculitis. Nephrol. Dial. Transpl. 30, I53–I59. 10.1093/ndt/gfu379 25523447

[B26] Saraswat M. Joenväärä S. Jain T. Tomar A. K. Sinha A. Singh S. (2016). Human spermatozoa quantitative proteomic signature classifies normo- and asthenozoospermia. Mol. Cell. Proteomics 16, 57–72. 10.1074/mcp.M116.061028 27895139 PMC5217782

[B27] Saraswat M. Joenväära S. Musante L. Peltoniemi H. Holthofer H. Renkonen R. (2015). N-linked (N-) glycoproteomics of urinary exosomes. [Corrected]. Mol. Cell. Proteomics. 14, 263–276. 10.1074/mcp.M114.040345 25452312 PMC4350024

[B28] Saraswat M. Nieminen H. Joenvaara S. Tohmola T. Seppänen H. Ristimäki A. (2019). Label-free serum proteomics and multivariate data analysis identifies biomarkers and expression trends that differentiate intraductal papillary mucinous neoplasia from pancreatic adenocarcinoma and healthy controls. Transl. Med. Commun. 4, 6. 10.1186/s41231-019-0037-4

[B29] Siljander P. Carpen O. Lassila R. (1996). Platelet-derived microparticles associate with fibrin during thrombosis. Blood 87, 4651–4663. 10.1182/blood.v87.11.4651.bloodjournal87114651 8639834

[B30] Skotland T. Ekroos K. Kauhanen D. Simolin H. Seierstad T. Berge V. (2017). Molecular lipid species in urinary exosomes as potential prostate cancer biomarkers. Eur. J. Cancer 70, 122–132. 10.1016/j.ejca.2016.10.011 27914242

[B31] Srikanthan S. Li W. Silverstein R. L. Mcintyre T. M. (2014). Exosome poly-ubiquitin inhibits platelet activation, downregulates Cd36 and inhibits pro-atherothombotic cellular functions. J. Thromb. Haemost. 12, 1906–1917. 10.1111/jth.12712 25163645 PMC4229405

[B32] Takahashi Y. Hosaka Y. Niina H. Nagasawa K. Naotsuka M. Sakai K. (1995). Soluble thrombomodulin purified from human urine exhibits A potent anticoagulant effect *in vitro* and *in vivo* . Thromb. Haemost. 73, 805–811. 10.1055/s-0038-1653872 7482407

[B33] Takemura T. Yoshioka K. Akano N. Miyamoto H. Matsumoto K. Maki S. (1987). Glomerular deposition of cross–linked fibrin in human kidney diseases. Kidney Int. 32, 102–111. 10.1038/ki.1987.178 3306094

[B34] Tavoosi N. Smith S. A. Davis-Harrison R. L. Morrissey J. H. (2013). Factor vii and protein C are phosphatidic acid-binding proteins. Biochemistry 52, 5545–5552. 10.1021/bi4006368 23879866 PMC3784305

[B35] Thery C. Ostrowski M. Segura E. (2009). Membrane vesicles as conveyors of immune responses. Nat. Rev. Immunol. 9, 581–593. 10.1038/nri2567 19498381

[B36] Valadi H. Ekstrom K. Bossios A. Sjostrand M. Lee J. J. Lotvall J. O. (2007). Exosome-mediated transfer of mrnas and micrornas is A novel mechanism of genetic exchange between cells. Nat. Cell Biol. 9, 654–659. 10.1038/ncb1596 17486113

[B37] Vassalli P. Mccluskey R. T. (1971). The pathogenetic role of the coagulation process in glomerular diseases of immunologic origin. Adv. Nephrol. Necker Hosp. 1, 47–63. 4143241

[B38] Von Dem Borne P. A. Mosnier L. O. Tans G. Meijers J. C. Bouma B. N. (1997). Factor xi activation by meizothrombin: Stimulation by phospholipid vesicles containing both phosphatidylserine and phosphatidylethanolamine. Thromb. Haemost. 78, 834–839. 10.1055/s-0038-1657637 9268180

[B39] Wiggins R. C. Glatfelter A. Kshirsagar B. Brukman J. (1986). Procoagulant activity in normal human urine associated with subcellular particles. Kidney Int. 29, 591–597. 10.1038/ki.1986.39 3702215

[B40] Wolf P. (1967). The nature and significance of platelet products in human plasma. Br. J. Haematol. 13, 269–288. 10.1111/j.1365-2141.1967.tb08741.x 6025241

