# Supplementary material for: Urinary extracellular vesicles carry multiple activators and regulators of coagulation
Source: Front Cell Dev Biol. 2022 Sep 7;10:967482. doi: 10.3389/fcell.2022.967482 (PMC9489905; doi:10.3389/fcell.2022.967482)
Supplement: Supplementary file 1 [file DataSheet2.PDF]

Urinary extracellular vesicles carry multiple activators and regulators of coagulation

Mayank Saraswat, Beata przybyla, Sakari Joenvaara, Tialotta Tohmola, Tomas Strandin, Maija Puhka, Annukka Jouppila, Riitta Lassila, Risto Renkonen

Supplementary Figures

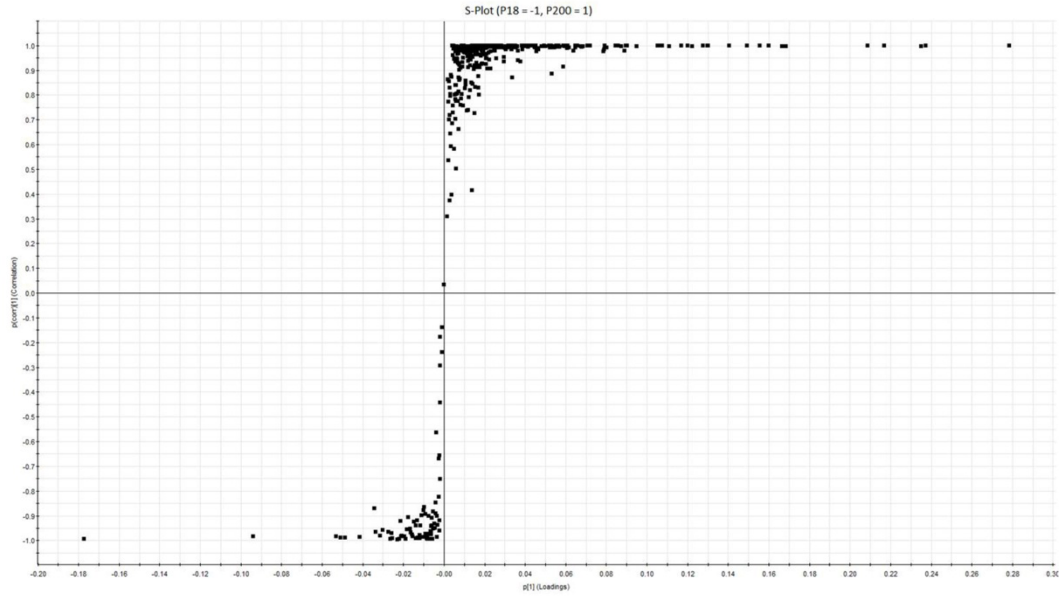

Supplementary Figure 1: S-Plot to show P18 and P200 differ in their proteome composition and abundance. Orthogonal projections to latent structures-discriminant analysis (OPLS-DA) modeling-based S-Plot was generated to find if the two pellets (P18 and P200) were truly different from each other. Loadings (p[1], X-axis) were plotted against correlation (p(Corr)[1], Y-axis) to generate S-Plot. Proteins separating these two pellets are given in supplementary Table 2.

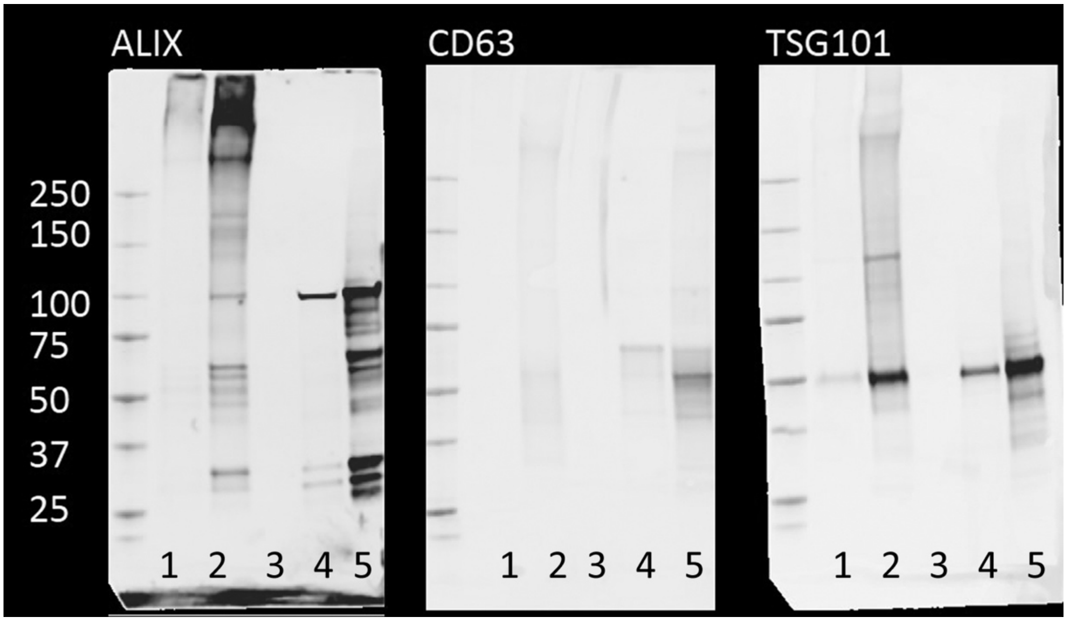

Supplementary Figure 2: Western blotting to show the molecular weight distribution of ALIX, CD63 and tumor susceptibility protein 101 (TSG101) in representative pooled samples. Lanes 1: P18 (non-reducing conditions) 2: P200 (non-reducing conditions) 3: Empty 4: P18 (reducing conditions) 5: P200 (reducing conditions). Molecular weight markers are shown as numbers on the extreme left. The images were converted to grayscale and inverted by Photoshop CS6. No other manipulation was involved.

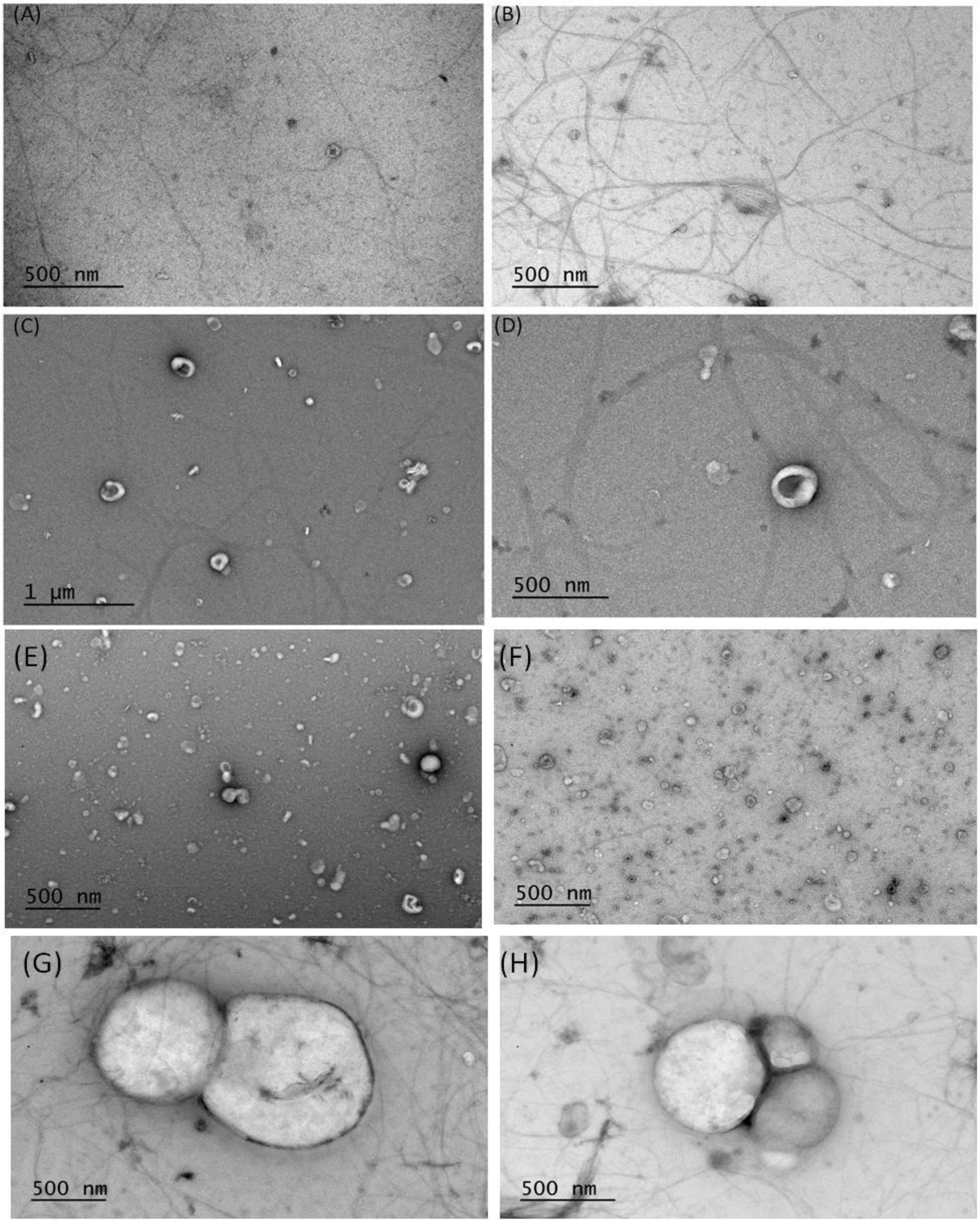

Supplementary figure 3: Transmission electron micrographs of (A), (B), (E) and (F) is exosomes (P200) and (C), (D), (G) and (H) are microvesicles (P18). Scale bars in (A), (B), (D) (E), (F), (G)

and (H) is 500 nm and in (C) is 1000 nm. (A), (B), (C) and (D) are from a single donor whereas (E), (F), (G) and (H) are from pools of eight donors.

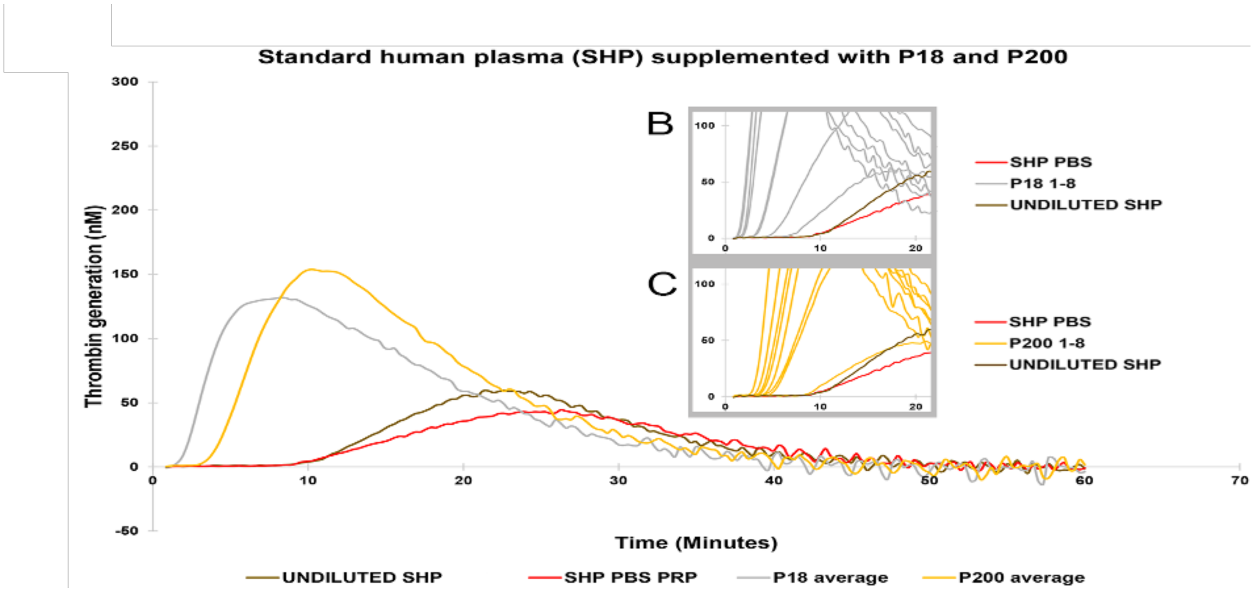

Supplementary Figure 4: Thrombin generation, as measured by Calibrated Automated Thrombogram (CAT) is enhanced upon supplementation of urinary extracellular vesicles (P18 and P200). EVs (P18 (Grey line, microvesicles) and P200 (Orange line, exosomes) were supplemented into Standard human plasma (SHP) (A, B and C), and thrombin generation was measured after challenging the plasma with PRP reagent (1pM tissue factor with no phospholipids). Undiluted plasma (Brown line) and diluted plasma (with PBS at same volume as EVs) was used a control (Red line). The measurement in A and B is shown as averages at every time point for eight different measurements for P18 and eight different measurements for P200. These eight measurements are EVs (P18 and P200) purified from eight different individuals and measured separately. The average was taken for ease of understanding and clarity in the figure. Inset diagrams (B and C for SHP) show a cut portion of the original diagram with eight separate individuals' measurements. Insets are shown to highlight the differences in lagtime (start of the

thrombin generation) between individuals which are not clear from averages in A and D. X-axis represents time and Y-axis thrombin generation.

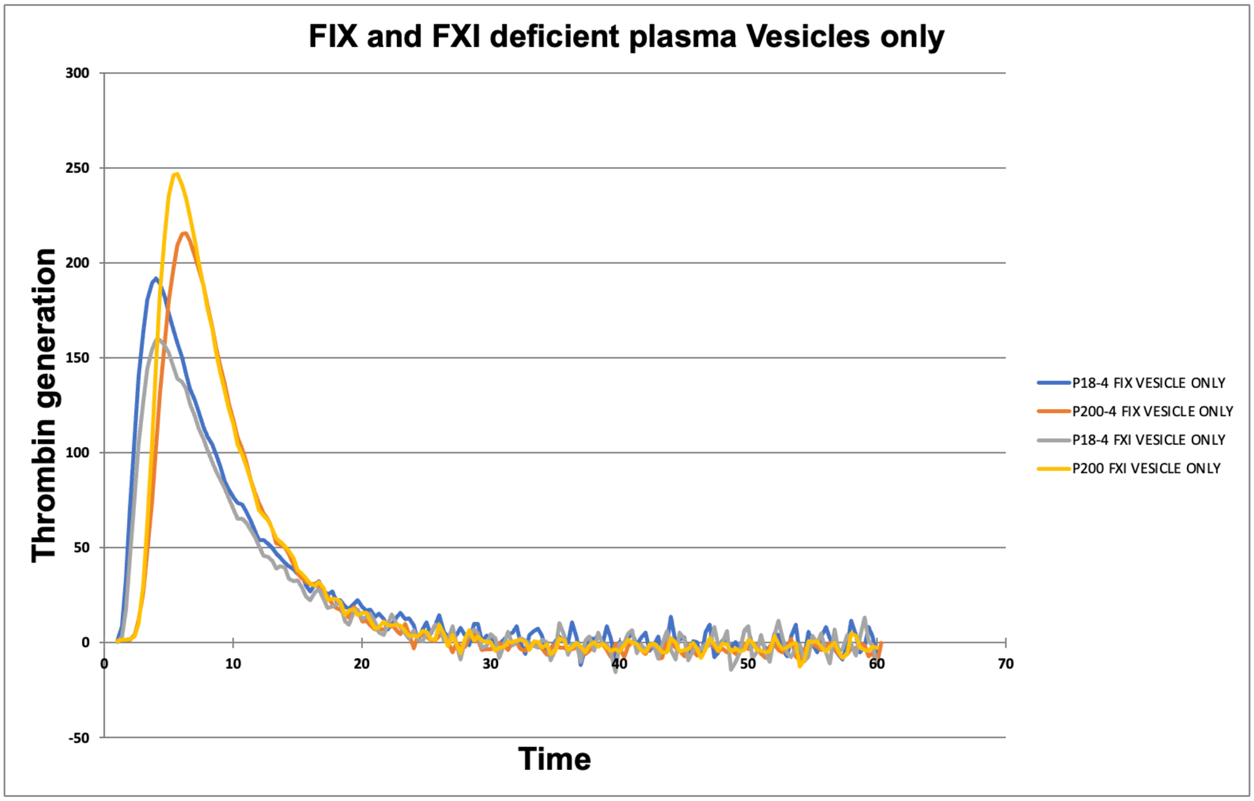

Supplementary Figure 5: Thrombin generation, as measured by Calibrated Automated Thrombogram (CAT) is observed in FIX- and FXI-deficient plasma without the trigger of exogenous tissue factor. EVs (P18 (microvesicles) and P200 (exosomes)) were supplemented into FIX and FXI deficient plasma, and thrombin generation was measured without challenging the plasma. The measurement is shown for exosomes (P200) and microvesicles (P18) from one representative sample (For comparison P18-4 and P200-4 from other figures). X-axis represents time and Y-axis thrombin generation.

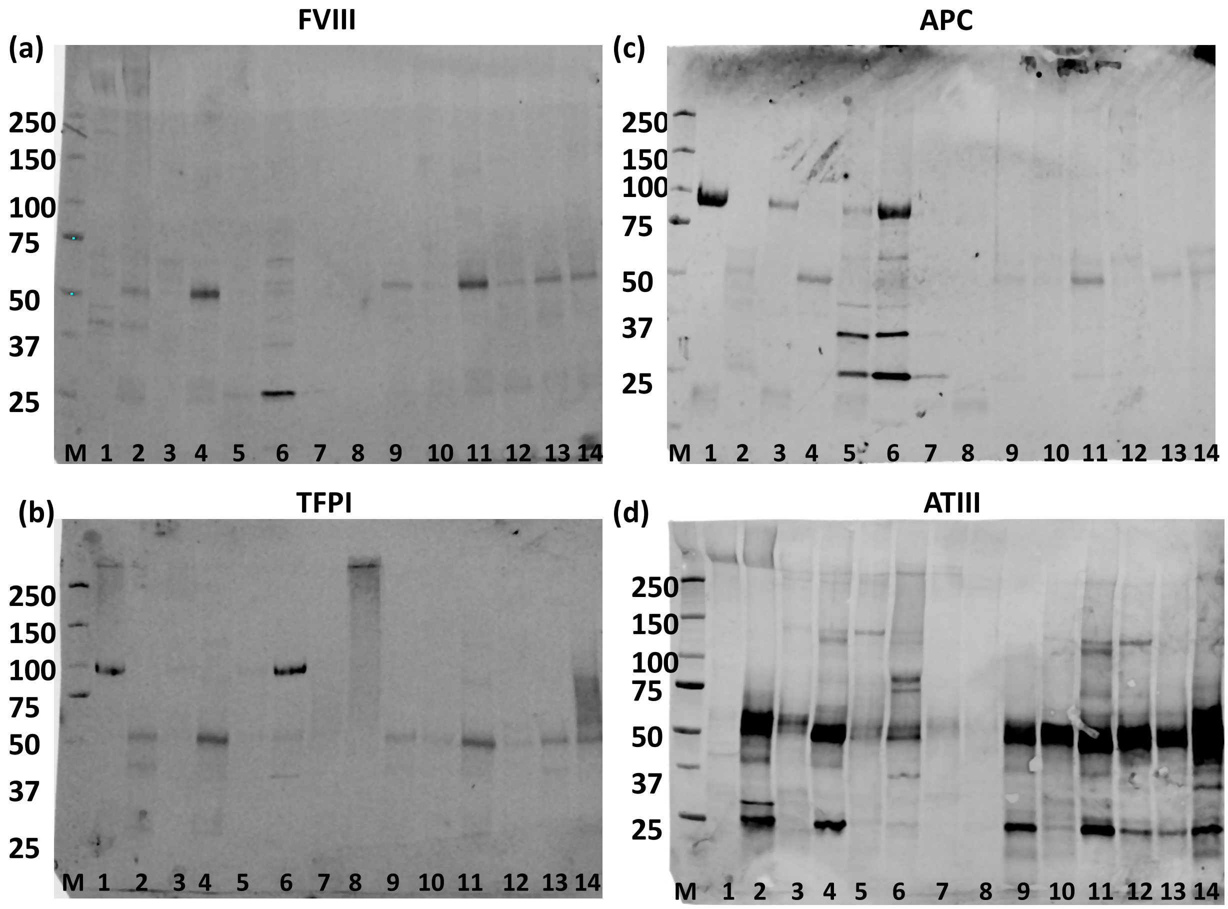

Supplementary Figure 6: Western blot of P18 and P200 using antibodies against (A) Factor VIII, (B) Tissue factor pathway inhibitor (TFPI), (C) activated protein C (APC) and (D) antithrombin (AT). Lane M: Marker Lanes 1 to 7: P18 from 7 individual donors, Lanes 8-14: P200 from 7 individual donors. All samples were boiled and processed in reducing conditions.

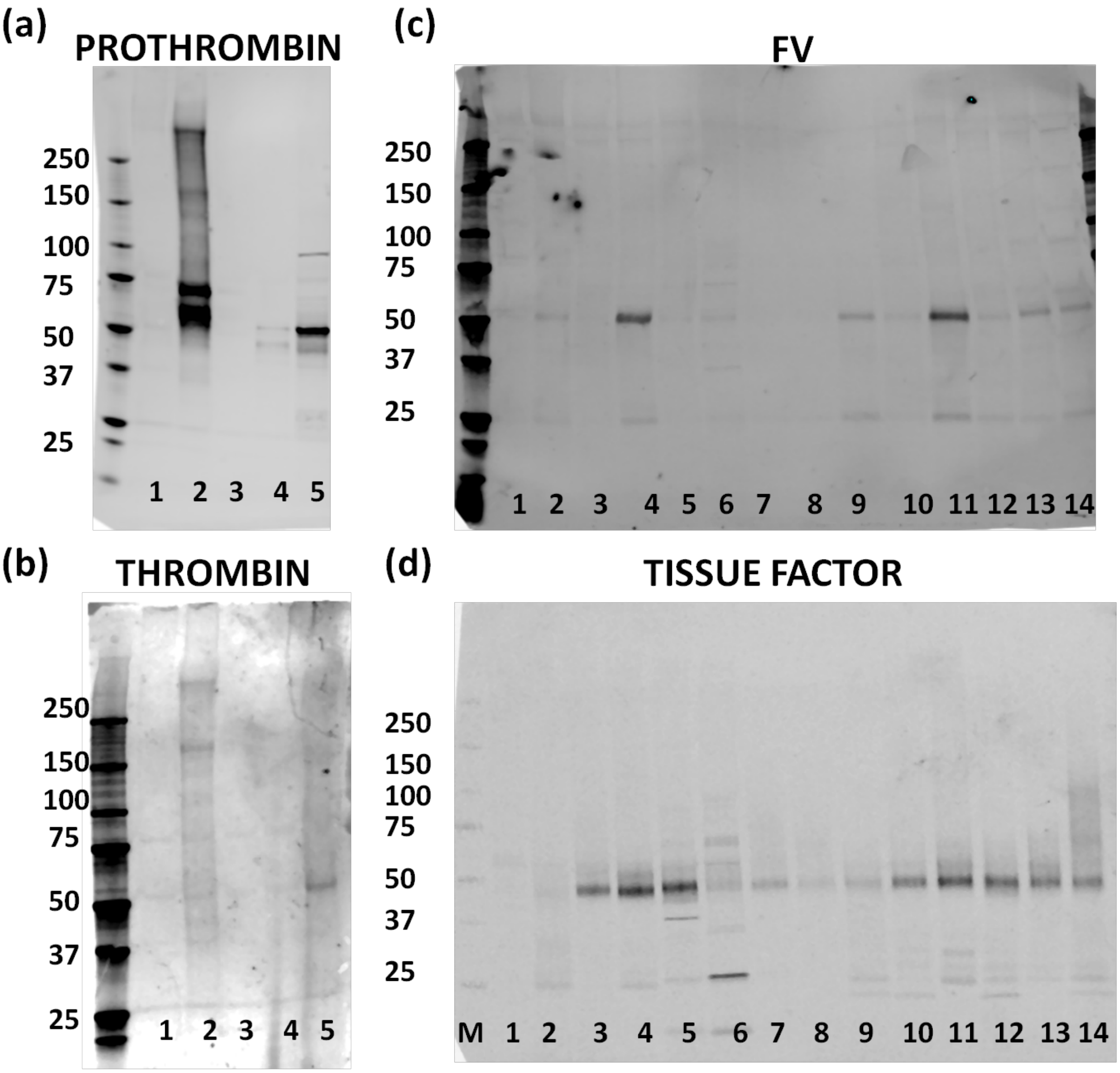

Supplementary Figure 7: Western blot analysis of P18 (microvesicles) and P200 (exosomes) in both reducing and non-reducing conditions using antibodies against prothrombin (FII), thrombin (FIIa), Factor V (FV) and Tissue factor (TF). Molecular weight markers are written as numbers in kDa from 250 to 25. In (a) and (b), Lanes 1: P18, non-reducing conditions, Lane 2: P200, non-reducing conditions, Lane 3: Empty, Lane 4: P18, reducing conditions (50mM DTT and boiling), Lane 5: P200, reducing conditions (50mM DTT and boiling). In (c) and (d), Lane M: Marker Lanes

1 to 7: P18 from 7 individual donors, Lanes 8-14: P200 from 7 individual donors. All samples were boiled and processed in reducing conditions.

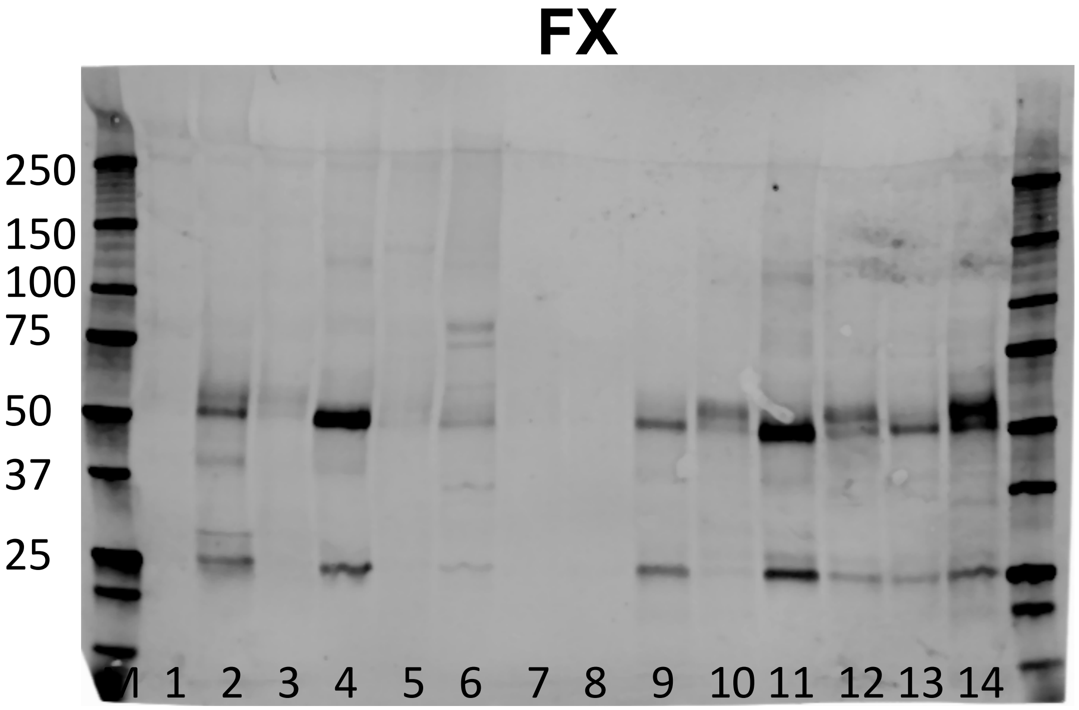

Supplementary Figure 8: Western blot of P18 and P200 using antibodies against Factor X. Lane M: Marker Lanes 1 to 7: P18 from 7 individual donors, Lanes 8-14: P200 from 7 individual donors. All samples were boiled and processed in reducing conditions.

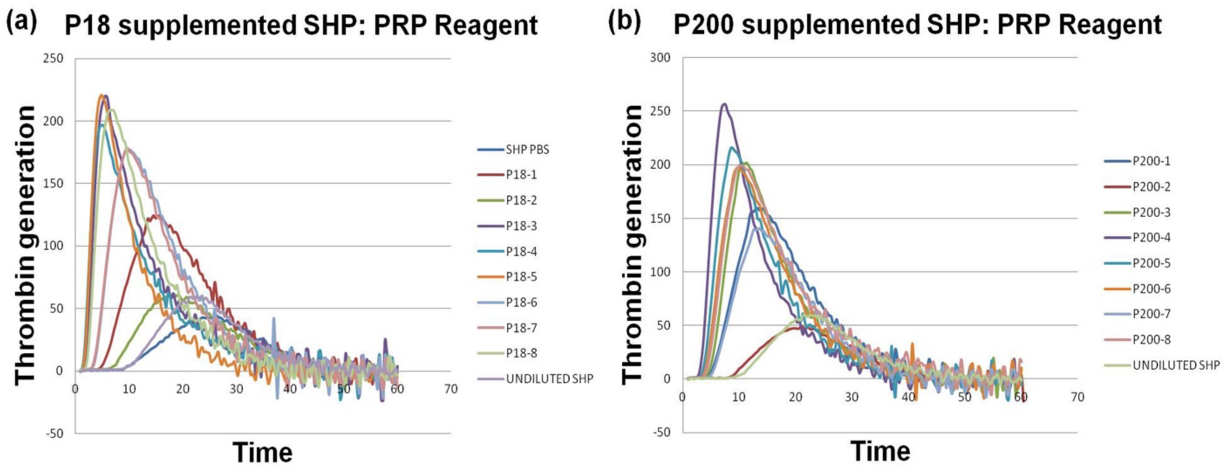

Supplementary Figure 9: Standard human plasma supplemented by P18 and P200 support enhanced thrombin generation. Thrombin generation was measured after challenging the plasma with PRP reagent (1pM tissue factor with no phospholipids) and either PBS or P18 (a) or P200 (b) was pre-added. This figure serves as a reference to supplementary figure 4, where average of these individual curves is shown. All P18 addition curves were significantly different from standard human plasma ($p < 0.05$), and in P200 one curve (P200-2) was not significant while all others were significant ($p < 0.05$). Endogenous thrombin potential (area under the curve) of P18 were significantly different from standard human plasma ($p < 0.05$) and in P200 one curve (P200-2) was not significant while all others were significant ($p < 0.05$).

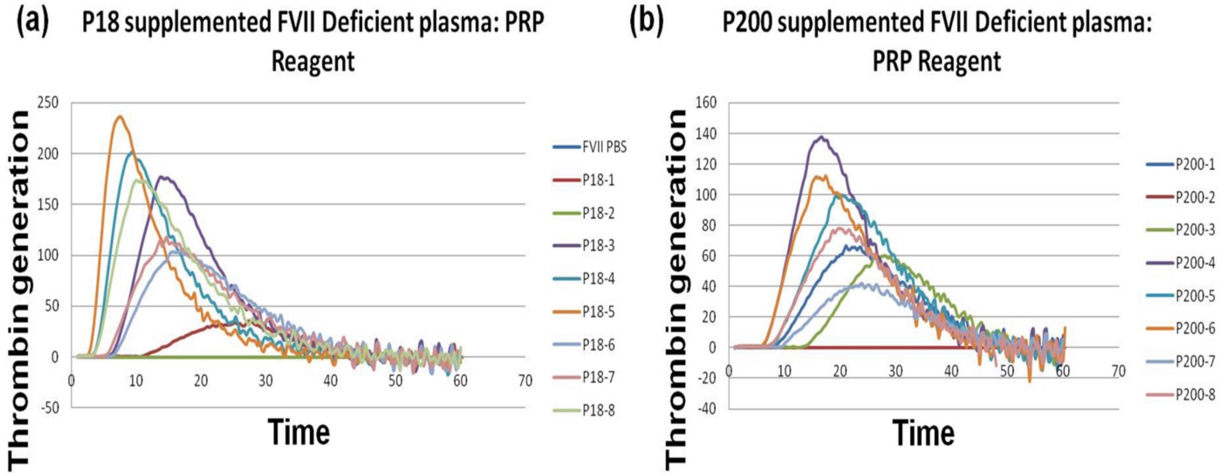

Supplementary Figure 10: P18 or P200, when added to FVII deficient plasma rescue thrombin generation. This figure serves as a reference to main text Figure 3A, where average P18 or P200 curves are shown. Thrombin generation was measured after challenging the deficient plasma with PRP reagent (1pM tissue factor with no phospholipids) and either PBS or P18 (a) or P200 (b) were pre-added. Control FVII deficient plasma diluted with PBS did not support thrombin generation therefore comparative statistics could not be applied.

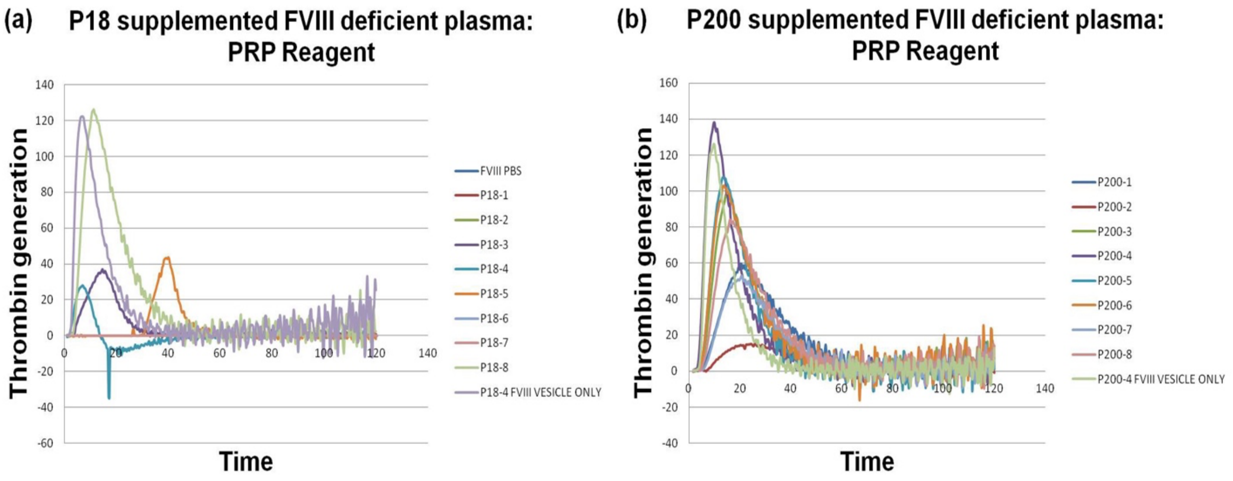

Supplementary Figure 11: P18 or P200, when added to FVIII deficient plasma rescue thrombin generation. This figure serves as a reference to main text Figure 3B, where average

P18 or P200 curves are shown. Thrombin generation was measured after challenging the deficient plasma with PRP reagent (1pM tissue factor with no phospholipids) and either PBS or P18 (a) or P200 (b) were pre-added. Control FVIII deficient plasma diluted with PBS did not support thrombin generation therefore comparative statistics could not be applied.

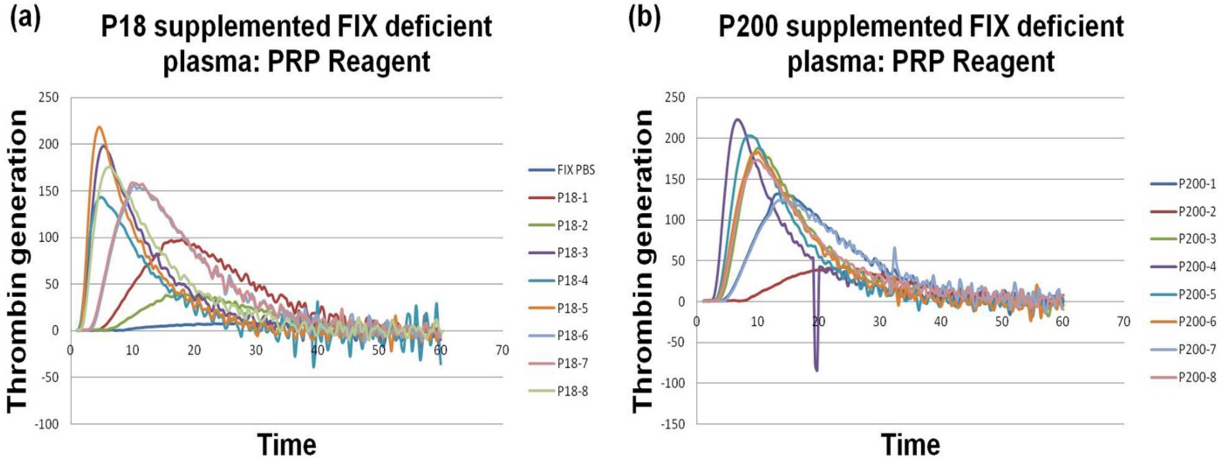

Supplementary Figure 12: P18 or P200, when added to FIX deficient plasma rescue thrombin generation. This figure serves as a reference to main text Figure 4A, where average P18 or P200 curves are shown. Thrombin generation was measured after challenging the deficient plasma with PRP reagent (1pM tissue factor with no phospholipids) and either PBS or P18 (a) or P200 (b) were pre-added. All P18 and P200 addition curves were significantly different from standard human plasma ($p < 0.05$) and so was endogenous thrombin potential (area under the curve, $p < 0.05$).

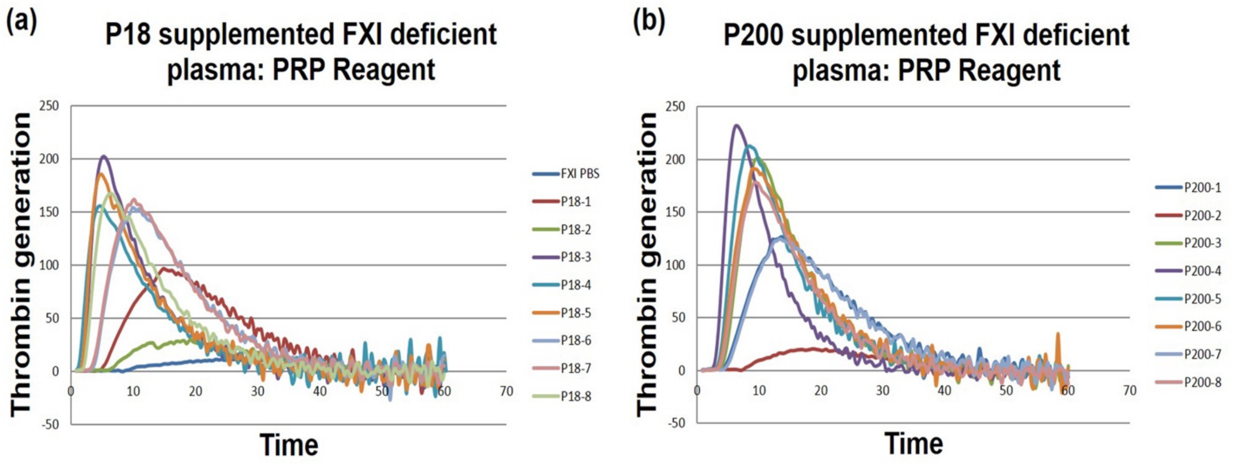

Supplementary Figure 13: P18 or P200, when added to FXI deficient plasma rescue thrombin generation. This figure serves as a reference to main text Figure 4B, where average P18 or P200 curves are shown. Thrombin generation was measured after challenging the deficient plasma with PRP reagent (1pM tissue factor with no phospholipids) and either PBS or P18 (a) or P200 (b) were pre-added. All P18 and P200 addition curves were significantly different from standard human plasma ($p < 0.05$) so was endogenous thrombin potential (area under the curve, $p < 0.05$).

Supplementary Table 1: All the protein identified in the study with 2 or more peptides. Peptide count, accession, Unique peptides, confidence score, ANOVA p values, maximum fold change between P18 and P200, and highest mean condition along with description of the protein are given in the table. Fold change is calculated by dividing the protein's relative abundance in highest mean condition by lowest mean condition.

Accession	Peptide count	Unique peptides	Confidence score	ANOVA (p)	Max fold change	Highest mean condition	Lowest mean condition	Description
A0PJK1	7	7	54.2086	0.142289	1.219414	P18	P200	Sodium/glucose cotransporter 5 OS=Homo sapiens GN=SLC5A10 PE=1 SV=2
A5A3E0	33	2	204.6087	0.227821	1.499102	P18	P200	POTE ankyrin domain family member F OS=Homo sapiens GN=POTEF PE=1 SV=2
O14745	25	23	262.3358	0.000742	2.018742	P18	P200	Na(+)/H(+) exchange regulatory cofactor NHE-RF1 OS=Homo sapiens GN=SLC9A3R1 PE=1 SV=4
O43707	28	20	177.5453	7.94E-05	1.539728	P18	P200	Alpha-actinin-4 OS=Homo sapiens GN=ACTN4 PE=1 SV=2
O75936	12	11	73.1624	0.787298	1.0173	P18	P200	Gamma-butyrobetaine dioxygenase OS=Homo sapiens GN=BBOX1 PE=1 SV=1
P00441	5	5	50.4342	0.023954	1.960582	P18	P200	Superoxide dismutase [Cu-Zn] OS=Homo sapiens GN=SOD1 PE=1 SV=2
P00491	4	2	20.8661	4.80E-05	3.133913	P18	P200	Purine nucleoside phosphorylase OS=Homo sapiens GN=PNP PE=1 SV=2
P00558	23	17	191.8436	0.010916	1.239913	P18	P200	Phosphoglycerate kinase 1 OS=Homo sapiens GN=PGK1 PE=1 SV=3
P00746	8	8	62.802	6.64E-05	11.74906	P18	P200	Complement factor D OS=Homo sapiens GN=CFD PE=1 SV=5
P00918	11	10	96.2537	0.007108	1.318685	P18	P200	Carbonic anhydrase 2 OS=Homo sapiens GN=CA2 PE=1 SV=2
P02647	18	18	148.5344	0.000126	1.770827	P18	P200	Apolipoprotein A-I OS=Homo sapiens GN=APOA1 PE=1 SV=1
P02679	9	8	58.6363	0.033894	1.241367	P18	P200	Fibrinogen gamma chain OS=Homo sapiens GN=FGG PE=1 SV=3
P02749	4	4	26.461	0.017123	1.455247	P18	P200	Beta-2-glycoprotein 1 OS=Homo sapiens GN=APOH PE=1 SV=3
P02753	9	9	64.4453	0.001055	1.835392	P18	P200	Retinol-binding protein 4 OS=Homo sapiens GN=RBP4 PE=1 SV=3
P04899	13	6	71.7357	0.091091	1.057763	P18	P200	Guanine nucleotide-binding protein G(i) subunit alpha-2 OS=Homo sapiens GN=GNAI2 PE=1 SV=3
P05023	19	9	106.2362	0.058177	1.783518	P18	P200	Sodium/potassium-transporting ATPase

								subunit alpha-1 OS=Homo sapiens GN=ATP1A1 PE=1 SV=1
P05062	24	20	189.2203	0.002369	1.204879	P18	P200	Fructose-bisphosphate aldolase B OS=Homo sapiens GN=ALDOB PE=1 SV=2
P05787	13	4	64.4844	0.690323	1.05123	P18	P200	Keratin, type II cytoskeletal 8 OS=Homo sapiens GN=KRT8 PE=1 SV=7
P06727	37	32	344.5497	0.002699	1.67934	P18	P200	Apolipoprotein A-IV OS=Homo sapiens GN=APOA4 PE=1 SV=3
P06733;P09104	25	21	240.2741	0.011706	1.137937	P18	P200	Alpha-enolase OS=Homo sapiens GN=ENO1 PE=1 SV=2
P07148	9	9	58.7109	0.005073	1.47067	P18	P200	Fatty acid-binding protein, liver OS=Homo sapiens GN=FABP1 PE=1 SV=1
P07195	20	15	138.1347	0.008523	1.238964	P18	P200	L-lactate dehydrogenase B chain OS=Homo sapiens GN=LDHB PE=1 SV=2
P07437	14	3	103.6779	0.006425	1.653962	P18	P200	Tubulin beta chain OS=Homo sapiens GN=TUBB PE=1 SV=2
P07737	8	8	67.2596	0.410542	1.098919	P18	P200	Profilin-1 OS=Homo sapiens GN=PFN1 PE=1 SV=2
P07864	4	3	20.2727	0.050807	1.7943	P18	P200	L-lactate dehydrogenase C chain OS=Homo sapiens GN=LDHC PE=1 SV=4
P07911	146	133	527.001	3.17E-05	1.686777	P18	P200	Uromodulin OS=Homo sapiens GN=UMOD PE=1 SV=1
P08238	20	8	140.9013	0.951609	1.010498	P18	P200	Heat shock protein HSP 90-beta OS=Homo sapiens GN=HSP90AB1 PE=1 SV=4
P08263;P09210;Q16772;Q7RTV2	9	6	65.0347	0.000654	2.60792	P18	P200	Glutathione S-transferase A1 OS=Homo sapiens GN=GSTA1 PE=1 SV=3
P08311	3	3	15.3184	7.02E-05	1.859028	P18	P200	Cathepsin G OS=Homo sapiens GN=CTSG PE=1 SV=2
P09211	5	5	49.8997	0.013651	1.192112	P18	P200	Glutathione S-transferase P OS=Homo sapiens GN=GSTP1 PE=1 SV=2
P09327	20	19	132.1546	0.001518	1.795536	P18	P200	Villin-1 OS=Homo sapiens GN=VIL1 PE=1 SV=4
P10451	2	2	11.7756	0.020681	1.460735	P18	P200	Osteopontin OS=Homo sapiens GN=SPP1 PE=1 SV=1
P10599	4	4	29.0223	0.253841	1.147099	P18	P200	Thioredoxin OS=Homo sapiens GN=TXN PE=1 SV=3
P11488	7	4	27.8579	0.042518	1.305709	P18	P200	Guanine nucleotide-binding protein G(t) subunit alpha-1 OS=Homo sapiens GN=GNAT1 PE=1 SV=5

P11766	3	2	26.5877	0.474413	1.098311	P18	P200	Alcohol dehydrogenase class-3 OS=Homo sapiens GN=ADH5 PE=1 SV=4
P12277	16	14	152.6855	0.083593	1.065727	P18	P200	Creatine kinase B-type OS=Homo sapiens GN=CKB PE=1 SV=1
P13489	2	2	10.3135	0.015165	1.680369	P18	P200	Ribonuclease inhibitor OS=Homo sapiens GN=RNH1 PE=1 SV=2
P13646	23	9	179.1512	0.001117	2.460677	P18	P200	Keratin, type I cytoskeletal 13 OS=Homo sapiens GN=KRT13 PE=1 SV=4
P14550	18	17	118.0378	0.515206	1.046383	P18	P200	Alcohol dehydrogenase [NADP(+)] OS=Homo sapiens GN=AKR1A1 PE=1 SV=3
P15311	55	28	392.0232	0.925082	1.005249	P18	P200	Ezrin OS=Homo sapiens GN=EZR PE=1 SV=4
P15531;P22392	2	2	9.9153	0.061545	3.30004	P18	P200	Nucleoside diphosphate kinase A OS=Homo sapiens GN=NME1 PE=1 SV=1
P16083	3	2	26.0098	0.070266	3.026635	P18	P200	Ribosyldihydronicotinamide dehydrogenase [quinone] OS=Homo sapiens GN=NQO2 PE=1 SV=5
P16870	3	2	19.2781	6.65E-05	2.713608	P18	P200	Carboxypeptidase E OS=Homo sapiens GN=CPE PE=1 SV=1
P17174	7	6	51.5346	0.002419	1.510526	P18	P200	Aspartate aminotransferase, cytoplasmic OS=Homo sapiens GN=GOT1 PE=1 SV=3
P17302	2	2	8.9995	0.002379	1.427174	P18	P200	Gap junction alpha-1 protein OS=Homo sapiens GN=GJA1 PE=1 SV=2
P18065	4	3	20.7598	0.000978	2.44089	P18	P200	Insulin-like growth factor-binding protein 2 OS=Homo sapiens GN=IGFBP2 PE=1 SV=2
P19013	27	19	193.2684	0.243616	1.229115	P18	P200	Keratin, type II cytoskeletal 4 OS=Homo sapiens GN=KRT4 PE=1 SV=4
P21266	7	6	37.823	0.000346	4.379611	P18	P200	Glutathione S-transferase Mu 3 OS=Homo sapiens GN=GSTM3 PE=1 SV=3
P21462	3	3	17.5909	7.25E-05	2.023746	P18	P200	fMet-Leu-Phe receptor OS=Homo sapiens GN=FPR1 PE=1 SV=3
P21695	4	4	27.7453	0.00124	1.319589	P18	P200	Glycerol-3-phosphate dehydrogenase [NAD(+)], cytoplasmic OS=Homo sapiens GN=GPD1 PE=1 SV=4
P21796	12	12	93.243	0.000581	1.410593	P18	P200	Voltage-dependent anion-selective channel protein 1 OS=Homo sapiens GN=VDAC1 PE=1 SV=2
P22314	4	3	20.5296	0.165587	1.15831	P18	P200	Ubiquitin-like modifier-activating enzyme 1 OS=Homo sapiens GN=UBA1 PE=1 SV=3
P23528;Q9Y281	8	7	69.2974	0.000243	1.944567	P18	P200	Cofilin-1 OS=Homo sapiens GN=CFL1 PE=1 SV=3

P27348	6	2	38.155	0.02632	1.609802	P18	P200	14-3-3 protein theta OS=Homo sapiens GN=YWHAQ PE=1 SV=1
P30041	9	8	77.0865	0.106178	1.210067	P18	P200	Peroxiredoxin-6 OS=Homo sapiens GN=PRDX6 PE=1 SV=3
P30044	5	4	33.8908	0.031435	1.935668	P18	P200	Peroxiredoxin-5, mitochondrial OS=Homo sapiens GN=PRDX5 PE=1 SV=4
P30084	2	2	9.9697	5.81E-05	5.630558	P18	P200	Enoyl-CoA hydratase, mitochondrial OS=Homo sapiens GN=ECHS1 PE=1 SV=4
P30086	11	11	81.1546	0.262259	1.387101	P18	P200	Phosphatidylethanolamine-binding protein 1 OS=Homo sapiens GN=PEBP1 PE=1 SV=3
P30613	4	3	20.7613	0.841267	1.011746	P18	P200	Pyruvate kinase PKLR OS=Homo sapiens GN=PKLR PE=1 SV=2
P31639	6	6	39.4378	0.700624	1.021775	P18	P200	Sodium/glucose cotransporter 2 OS=Homo sapiens GN=SLC5A2 PE=1 SV=1
P31946	8	3	50.6014	0.202166	1.32099	P18	P200	14-3-3 protein beta/alpha OS=Homo sapiens GN=YWHAB PE=1 SV=3
P35241	30	5	164.9296	0.000473	1.932486	P18	P200	Radixin OS=Homo sapiens GN=RDX PE=1 SV=1
P35558	16	12	92.0221	0.004341	1.224211	P18	P200	Phosphoenolpyruvate carboxykinase, cytosolic [GTP] OS=Homo sapiens GN=PCK1 PE=1 SV=3
P35579	11	10	58.8357	0.660247	1.018134	P18	P200	Myosin-9 OS=Homo sapiens GN=MYH9 PE=1 SV=4
P35609	7	2	40.9181	0.004969	2.607609	P18	P200	Alpha-actinin-2 OS=Homo sapiens GN=ACTN2 PE=1 SV=1
P40925	9	8	61.155	0.00044	1.636076	P18	P200	Malate dehydrogenase, cytoplasmic OS=Homo sapiens GN=MDH1 PE=1 SV=4
P42330	3	3	15.5777	0.006601	2.583392	P18	P200	Aldo-keto reductase family 1 member C3 OS=Homo sapiens GN=AKR1C3 PE=1 SV=4
P43490	3	2	13.9039	0.002719	1.384188	P18	P200	Nicotinamide phosphoribosyltransferase OS=Homo sapiens GN=NAMPT PE=1 SV=1
P47755	5	3	34.2544	0.499909	1.119932	P18	P200	F-actin-capping protein subunit alpha-2 OS=Homo sapiens GN=CAPZA2 PE=1 SV=3
P48735	6	4	32.3206	0.055947	1.580967	P18	P200	Isocitrate dehydrogenase [NADP], mitochondrial OS=Homo sapiens GN=IDH2 PE=1 SV=2
P49189	5	3	25.1243	0.109384	2.6188	P18	P200	4-trimethylaminobutyraldehyde dehydrogenase OS=Homo sapiens

								GN=ALDH9A1 PE=1 SV=3
P50226;P50225	2	2	10.3522	0.000167	3.183576	P18	P200	Sulfotransferase 1A2 OS=Homo sapiens GN=SULT1A2 PE=1 SV=2
P50502;Q8IZP2	2	2	11.4293	0.009677	2.699544	P18	P200	Hsc70-interacting protein OS=Homo sapiens GN=ST13 PE=1 SV=2
P60174	16	15	148.2727	0.003368	1.356889	P18	P200	Triosephosphate isomerase OS=Homo sapiens GN=TPI1 PE=1 SV=3
P60660	6	4	45.919	0.929883	1.00474	P18	P200	Myosin light polypeptide 6 OS=Homo sapiens GN=MYL6 PE=1 SV=2
P60709;P63261	80	25	589.8953	0.000375	1.682214	P18	P200	Actin, cytoplasmic 1 OS=Homo sapiens GN=ACTB PE=1 SV=1
P61158;Q9C0K3;Q9P1U1	10	9	64.828	0.538234	1.048979	P18	P200	Actin-related protein 3 OS=Homo sapiens GN=ACTR3 PE=1 SV=3
P61981	7	3	44.055	1.80E-05	5.013078	P18	P200	14-3-3 protein gamma OS=Homo sapiens GN=YWHAG PE=1 SV=2
P62158	7	6	46.2951	0.007647	1.346468	P18	P200	Calmodulin OS=Homo sapiens GN=CALM1 PE=1 SV=2
P63096	12	3	63.1799	7.51E-06	7.663907	P18	P200	Guanine nucleotide-binding protein G(i) subunit alpha-1 OS=Homo sapiens GN=GNAI1 PE=1 SV=2
P68133;P62736;P63267;P68032	51	3	336.4933	5.34E-05	4.091547	P18	P200	Actin, alpha skeletal muscle OS=Homo sapiens GN=ACTA1 PE=1 SV=1
P68363;Q13748;Q6PEY2;Q71U36;Q9BQE3;Q9NY65	14	3	87.3776	0.005681	1.532157	P18	P200	Tubulin alpha-1B chain OS=Homo sapiens GN=TUBA1B PE=1 SV=1
P78371	2	2	9.3735	0.006239	2.572595	P18	P200	T-complex protein 1 subunit beta OS=Homo sapiens GN=CCT2 PE=1 SV=4
Q2M2I5	10	5	63.1688	0.000894	1.279319	P18	P200	Keratin, type I cytoskeletal 24 OS=Homo sapiens GN=KRT24 PE=1 SV=1
Q5T2W1;A8MUH7	33	29	306.2869	0.000651	1.634577	P18	P200	Na(+)/H(+) exchange regulatory cofactor NHE-RF3 OS=Homo sapiens GN=PDZK1 PE=1 SV=2
Q5VTE0;P68104;Q05639	16	15	109.3506	0.434268	1.028455	P18	P200	Putative elongation factor 1-alpha-like 3 OS=Homo sapiens GN=EEF1A1P5 PE=5 SV=1
Q6KB66	2	2	10.5285	0.010075	2.423942	P18	P200	Keratin, type II cytoskeletal 80 OS=Homo sapiens GN=KRT80 PE=1 SV=2
Q8NFJ5	2	2	8.5511	0.003991	4.213991	P18	P200	Retinoic acid-induced protein 3 OS=Homo sapiens GN=GPRC5A PE=1 SV=2
Q9H2M3	9	3	61.8956	0.030259	2.270447	P18	P200	S-methylmethionine--homocysteine S-methyltransferase

								BHMT2 OS=Homo sapiens GN=BHMT2 PE=1 SV=1
Q9H6S3	7	7	49.7585	0.017411	1.205315	P18	P200	Epidermal growth factor receptor kinase substrate 8-like protein 2 OS=Homo sapiens GN=EPS8L2 PE=1 SV=2
Q9HCU0	2	2	9.8813	0.436115	1.093363	P18	P200	Endosialin OS=Homo sapiens GN=CD248 PE=1 SV=1
Q9UBI6	5	5	39.3869	0.737019	1.018529	P18	P200	Guanine nucleotide-binding protein G(I)/G(S)/G(O) subunit gamma-12 OS=Homo sapiens GN=GNG12 PE=1 SV=3
Q9UHG0	2	2	14.7012	0.001971	2.305375	P18	P200	Doublecortin domain-containing protein 2 OS=Homo sapiens GN=DCDC2 PE=1 SV=2
Q9UM54	5	5	29.2188	0.00404	2.098248	P18	P200	Unconventional myosin-VI OS=Homo sapiens GN=MYO6 PE=1 SV=4
Q9UQ52	4	2	18.1933	0.242231	1.177367	P18	P200	Contactin-6 OS=Homo sapiens GN=CNTN6 PE=1 SV=1
Q9Y5I7	2	2	10.2636	0.015876	1.509406	P18	P200	Claudin-16 OS=Homo sapiens GN=CLDN16 PE=1 SV=1
Q9Y536	6	2	25.4227	0.001366	3.041867	P18	P200	Peptidyl-prolyl cis-trans isomerase A-like 4A OS=Homo sapiens GN=PPIAL4A PE=2 SV=1
Q9Y696	21	16	162.7207	9.47E-05	1.819931	P18	P200	Chloride intracellular channel protein 4 OS=Homo sapiens GN=CLIC4 PE=1 SV=4
Q96EB1	2	2	4.799	0.263637	1.455153	P18	P200	Elongator complex protein 4 OS=Homo sapiens GN=ELP4 PE=1 SV=2
Q96KP4	24	22	164.6912	0.626884	1.015684	P18	P200	Cytosolic non-specific dipeptidase OS=Homo sapiens GN=CNDP2 PE=1 SV=2
Q00325	3	3	17.9835	0.001208	3.112391	P18	P200	Phosphate carrier protein, mitochondrial OS=Homo sapiens GN=SLC25A3 PE=1 SV=2
Q695T7	3	2	13.428	0.665489	1.04964	P18	P200	Sodium-dependent neutral amino acid transporter B(0)AT1 OS=Homo sapiens GN=SLC6A19 PE=1 SV=1
Q03154	30	27	208.1992	0.001413	1.335899	P18	P200	Aminoacylase-1 OS=Homo sapiens GN=ACY1 PE=1 SV=1
Q08257	2	2	10.6579	0.023573	1.627876	P18	P200	Quinone oxidoreductase OS=Homo sapiens GN=CRYZ PE=1 SV=1
Q13113	4	4	31.8754	0.015637	1.398874	P18	P200	PDZK1-interacting protein 1 OS=Homo sapiens GN=PDZK1IP1 PE=1 SV=1
Q13509	8	2	60.7652	0.011939	1.645913	P18	P200	Tubulin beta-3 chain OS=Homo sapiens GN=TUBB3 PE=1 SV=2

Q13621	17	16	120.7044	0.680142	1.042513	P18	P200	Solute carrier family 12 member 1 OS=Homo sapiens GN=SLC12A1 PE=1 SV=2
Q14019	3	3	26.4901	0.009572	25.25246	P18	P200	Coactosin-like protein OS=Homo sapiens GN=COTL1 PE=1 SV=3
Q14247	2	2	10.2336	0.001868	2.588666	P18	P200	Src substrate cortactin OS=Homo sapiens GN=CTTN PE=1 SV=2
Q14508	3	3	20.6646	0.000117	2.605106	P18	P200	WAP four-disulfide core domain protein 2 OS=Homo sapiens GN=WFDC2 PE=1 SV=2
Q14651	2	2	16.8959	0.016072	1.711345	P18	P200	Plastin-1 OS=Homo sapiens GN=PLS1 PE=1 SV=2
Q15181	3	3	14.1385	0.003793	1.554335	P18	P200	Inorganic pyrophosphatase OS=Homo sapiens GN=PPA1 PE=1 SV=2
Q15365;P57721;Q15366	3	3	18.2825	0.0519	1.439524	P18	P200	Poly(rC)-binding protein 1 OS=Homo sapiens GN=PCBP1 PE=1 SV=2
Q15833	4	3	26.6033	0.163032	1.199968	P18	P200	Syntaxin-binding protein 2 OS=Homo sapiens GN=STXBP2 PE=1 SV=2
Q93088	21	16	192.8418	0.361588	1.026732	P18	P200	Betaine--homocysteine S-methyltransferase 1 OS=Homo sapiens GN=BHMT PE=1 SV=2
A6NFK2	3	2	15.4644	3.22E-06	5.393008	P200	P18	Glutaredoxin domain-containing cysteine-rich protein 2 OS=Homo sapiens GN=GRXCR2 PE=3 SV=1
B9A064;P0CG04	12	2	115.6017	3.79E-06	47.03396	P200	P18	Immunoglobulin lambda-like polypeptide 5 OS=Homo sapiens GN=IGLL5 PE=2 SV=2
O00159	9	7	53.4205	0.000373	5.103956	P200	P18	Unconventional myosin-Ic OS=Homo sapiens GN=MYO1C PE=1 SV=4
O00161	2	2	10.6793	0.018247	2.098926	P200	P18	Synaptosomal-associated protein 23 OS=Homo sapiens GN=SNAP23 PE=1 SV=1
O00187	20	16	139.9892	5.96E-06	2.952286	P200	P18	Mannan-binding lectin serine protease 2 OS=Homo sapiens GN=MASP2 PE=1 SV=4
O00299	7	5	57.9226	1.27E-06	4.954057	P200	P18	Chloride intracellular channel protein 1 OS=Homo sapiens GN=CLIC1 PE=1 SV=4
O00322	8	7	62.7235	0.00032	2.282185	P200	P18	Uroplakin-1a OS=Homo sapiens GN=UPK1A PE=2 SV=1
O00468	26	24	148.9278	8.07E-06	4.637168	P200	P18	Agrin OS=Homo sapiens GN=AGRN PE=1 SV=5
O00526	6	6	36.8072	0.006739	1.402181	P200	P18	Uroplakin-2 OS=Homo sapiens GN=UPK2 PE=1 SV=2
O00560	27	25	299.5981	3.58E-07	13.88041	P200	P18	Syntenin-1 OS=Homo sapiens GN=SDCBP PE=1 SV=1
O00584	5	5	34.5321	0.000169	24.96952	P200	P18	Ribonuclease T2 OS=Homo sapiens

								GN=RNASET2 PE=1 SV=2
O00592	5	5	23.9791	0.491912	1.230258	P200	P18	Podocalyxin OS=Homo sapiens GN=PODXL PE=1 SV=2
O00754	13	13	93.2475	4.56E-06	5.400517	P200	P18	Lysosomal alpha-mannosidase OS=Homo sapiens GN=MAN2B1 PE=1 SV=3
O14672	2	2	8.6262	0.001815	2.981732	P200	P18	Disintegrin and metalloproteinase domain-containing protein 10 OS=Homo sapiens GN=ADAM10 PE=1 SV=1
O14773	16	14	142.042	3.44E-07	14.9402	P200	P18	Tripeptidyl-peptidase 1 OS=Homo sapiens GN=TPP1 PE=1 SV=2
O15144	3	3	17.7732	0.005432	1.67814	P200	P18	Actin-related protein 2/3 complex subunit 2 OS=Homo sapiens GN=ARPC2 PE=1 SV=1
O15162	2	2	11.5064	0.010114	24.07671	P200	P18	Phospholipid scramblase 1 OS=Homo sapiens GN=PLSCR1 PE=1 SV=1
O15393	9	9	50.1289	3.57E-05	59.9443	P200	P18	Transmembrane protease serine 2 OS=Homo sapiens GN=TMPRSS2 PE=1 SV=3
O15484	4	4	19.4479	2.20E-07	20.4226	P200	P18	Calpain-5 OS=Homo sapiens GN=CAPN5 PE=1 SV=2
O43172	4	4	17.5923	5.64E-06	5.677486	P200	P18	U4/U6 small nuclear ribonucleoprotein Prp4 OS=Homo sapiens GN=PRPF4 PE=1 SV=2
O43396	3	3	13.2276	3.43E-07	10.89494	P200	P18	Thioredoxin-like protein 1 OS=Homo sapiens GN=TXNL1 PE=1 SV=3
O43451	58	51	489.1954	1.02E-06	11.58549	P200	P18	Maltase-glucoamylase, intestinal OS=Homo sapiens GN=MGAM PE=1 SV=5
O43490	26	26	261.5175	4.74E-07	17.86139	P200	P18	Prominin-1 OS=Homo sapiens GN=PROM1 PE=1 SV=1
O43633	11	11	84.0451	4.99E-06	3.971843	P200	P18	Charged multivesicular body protein 2a OS=Homo sapiens GN=CHMP2A PE=1 SV=1
O43653	11	11	63.5559	1.19E-05	4.227666	P200	P18	Prostate stem cell antigen OS=Homo sapiens GN=PSCA PE=1 SV=1
O43719	4	2	20.7401	6.09E-05	11.93222	P200	P18	HIV Tat-specific factor 1 OS=Homo sapiens GN=HTATSF1 PE=1 SV=1
O43895	23	17	155.8148	0.000123	2.455201	P200	P18	Xaa-Pro aminopeptidase 2 OS=Homo sapiens GN=XPNPEP2 PE=2 SV=3
O60282	4	3	20.4169	3.66E-05	231.1451	P200	P18	Kinesin heavy chain isoform 5C OS=Homo sapiens GN=KIF5C PE=1 SV=1
O60635	7	7	56.1424	2.50E-07	13.33097	P200	P18	Tetraspanin-1 OS=Homo sapiens GN=TSPAN1 PE=1 SV=2

O60701	2	2	10.0152	0.516472	1.241455	P200	P18	UDP-glucose 6-dehydrogenase OS=Homo sapiens GN=UGDH PE=1 SV=1
O60784	4	4	19.7697	1.70E-05	6.826414	P200	P18	Target of Myb protein 1 OS=Homo sapiens GN=TOM1 PE=1 SV=2
O75083	9	8	68.4352	1.01E-05	6.283885	P200	P18	WD repeat-containing protein 1 OS=Homo sapiens GN=WDR1 PE=1 SV=4
O75131	6	3	32.9887	2.20E-07	12.86486	P200	P18	Copine-3 OS=Homo sapiens GN=CPNE3 PE=1 SV=1
O75264	5	5	30.7011	0.000163	2.389801	P200	P18	Small integral membrane protein 24 OS=Homo sapiens GN=SMIM24 PE=2 SV=2
O75340	9	7	78.8172	1.93E-05	4.720485	P200	P18	Programmed cell death protein 6 OS=Homo sapiens GN=PDCD6 PE=1 SV=1
O75351	14	9	103.4219	1.36E-06	6.257083	P200	P18	Vacuolar protein sorting-associated protein 4B OS=Homo sapiens GN=VPS4B PE=1 SV=2
O75436	3	3	20.2301	0.015699	1.444735	P200	P18	Vacuolar protein sorting-associated protein 26A OS=Homo sapiens GN=VPS26A PE=1 SV=2
O75478	3	3	17.7144	7.22E-08	12.15622	P200	P18	Transcriptional adapter 2-alpha OS=Homo sapiens GN=TADA2A PE=1 SV=3
O75594	4	4	51.4256	3.99E-06	11.73819	P200	P18	Peptidoglycan recognition protein 1 OS=Homo sapiens GN=PGLYRP1 PE=1 SV=1
O75631	4	4	25.7959	0.000187	2.088864	P200	P18	Uroplakin-3a OS=Homo sapiens GN=UPK3A PE=1 SV=3
O75635	2	2	9.1829	0.000666	4.765783	P200	P18	Serpin B7 OS=Homo sapiens GN=SERPINB7 PE=1 SV=1
O75787	5	4	38.3856	4.49E-06	5.775357	P200	P18	Renin receptor OS=Homo sapiens GN=ATP6AP2 PE=1 SV=2
O75874	20	17	124.5856	0.000379	2.166502	P200	P18	Isocitrate dehydrogenase [NADP] cytoplasmic OS=Homo sapiens GN=IDH1 PE=1 SV=2
O75882	36	33	281.4914	1.21E-05	7.497905	P200	P18	Attractin OS=Homo sapiens GN=ATRN PE=1 SV=2
O75891	19	18	126.4026	0.009763	1.246352	P200	P18	Cytosolic 10-formyltetrahydrofolate dehydrogenase OS=Homo sapiens GN=ALDH1L1 PE=1 SV=2
O75955	7	7	36.7511	3.12E-05	8.917542	P200	P18	Flotillin-1 OS=Homo sapiens GN=FLOT1 PE=1 SV=3
O76014;O76015	4	2	22.2494	7.65E-06	187.472	P200	P18	Keratin, type I cuticular Ha7 OS=Homo sapiens GN=KRT37 PE=3 SV=3
O94760	3	2	18.7823	8.40E-06	14.19506	P200	P18	N(G),N(G)-dimethylarginine dimethylaminohydrolase

								1 OS=Homo sapiens GN=DDAH1 PE=1 SV=3
O95154	12	10	105.8657	0.004178	1.397434	P200	P18	Aflatoxin B1 aldehyde reductase member 3 OS=Homo sapiens GN=AKR7A3 PE=1 SV=2
O95336	9	9	66.8758	8.34E-06	4.207555	P200	P18	6-phosphogluconolactonase OS=Homo sapiens GN=PGLS PE=1 SV=2
O95865	7	5	53.4419	1.66E-06	1.9707	P200	P18	N(G),N(G)-dimethylarginine dimethylaminohydrolase 2 OS=Homo sapiens GN=DDAH2 PE=1 SV=1
O95954	9	6	57.0429	0.006505	1.299149	P200	P18	Formimidoyltransferase-cyclodeaminase OS=Homo sapiens GN=FTCD PE=1 SV=2
O95967	5	4	32.0261	4.24E-05	3.378542	P200	P18	EGF-containing fibulin-like extracellular matrix protein 2 OS=Homo sapiens GN=EFEMP2 PE=1 SV=3
O96006	4	2	21.8401	0.000128	3.195818	P200	P18	Zinc finger BED domain-containing protein 1 OS=Homo sapiens GN=ZBED1 PE=1 SV=1
O96009	24	23	129.738	6.99E-06	4.474548	P200	P18	Napsin-A OS=Homo sapiens GN=NAPSA PE=1 SV=1
P0DJD8;P0DJD7;P0DJD9	12	12	34.9218	8.91E-07	12.31001	P200	P18	Pepsin A-3 OS=Homo sapiens GN=PGA3 PE=1 SV=1
P0DMV8;P0DMV9	16	9	106.0384	0.018178	1.665317	P200	P18	Heat shock 70 kDa protein 1A OS=Homo sapiens GN=HSPA1A PE=1 SV=1
P00338	14	8	87.338	0.001386	1.653291	P200	P18	L-lactate dehydrogenase A chain OS=Homo sapiens GN=LDHA PE=1 SV=2
P00352	17	15	128.3869	0.000131	1.845944	P200	P18	Retinal dehydrogenase 1 OS=Homo sapiens GN=ALDH1A1 PE=1 SV=2
P00450	55	53	493.1579	9.52E-09	31.46597	P200	P18	Ceruloplasmin OS=Homo sapiens GN=CP PE=1 SV=1
P00734	10	10	78.2151	1.16E-06	3.543495	P200	P18	Prothrombin OS=Homo sapiens GN=F2 PE=1 SV=2
P00738;P00739	32	30	303.721	2.86E-07	7.854765	P200	P18	Haptoglobin OS=Homo sapiens GN=HP PE=1 SV=1
P00747	24	22	150.4732	0.000658	1.848367	P200	P18	Plasminogen OS=Homo sapiens GN=PLG PE=1 SV=2
P00749	6	5	42.1427	9.93E-05	7.40295	P200	P18	Urokinase-type plasminogen activator OS=Homo sapiens GN=PLAU PE=1 SV=2
P00751	7	7	36.8775	0.016659	2.299438	P200	P18	Complement factor B OS=Homo sapiens GN=CFB PE=1 SV=2
P00966	27	25	211.7525	0.122795	1.211745	P200	P18	Argininosuccinate synthase OS=Homo

								sapiens GN=ASS1 PE=1 SV=2
P01008	5	5	27.6251	1.31E-05	7.78427	P200	P18	Antithrombin-III OS=Homo sapiens GN=SERPINC1 PE=1 SV=1
P01009	39	35	361.7852	5.45E-07	11.43272	P200	P18	Alpha-1-antitrypsin OS=Homo sapiens GN=SERPINA1 PE=1 SV=3
P01011	16	16	114.0075	2.54E-08	10.86453	P200	P18	Alpha-1-antichymotrypsin OS=Homo sapiens GN=SERPINA3 PE=1 SV=2
P01023	25	24	177.1772	7.20E-07	8.401645	P200	P18	Alpha-2-macroglobulin OS=Homo sapiens GN=A2M PE=1 SV=3
P01024	68	63	564.3867	5.30E-07	6.351114	P200	P18	Complement C3 OS=Homo sapiens GN=C3 PE=1 SV=2
P01034	6	4	53.2858	0.011436	1.510522	P200	P18	Cystatin-C OS=Homo sapiens GN=CST3 PE=1 SV=1
P01042	56	51	418.3305	2.85E-07	6.387758	P200	P18	Kininogen-1 OS=Homo sapiens GN=KNG1 PE=1 SV=2
P01133	115	114	849.2033	2.48E-08	16.12683	P200	P18	Pro-epidermal growth factor OS=Homo sapiens GN=EGF PE=1 SV=2
P01591	20	20	116.825	1.25E-07	32.2929	P200	P18	Immunoglobulin J chain OS=Homo sapiens GN=JCHAIN PE=1 SV=4
P01597	2	2	11.7964	0.008958	171.4752	P200	P18	Ig kappa chain V-I region DEE OS=Homo sapiens PE=1 SV=1
P01617;P01614;P06309;P06310	4	2	43.0749	2.08E-05	4.29825	P200	P18	Ig kappa chain V-II region TEW OS=Homo sapiens PE=1 SV=1
P01623;P01620;P01622;P04206;P18135;P18136	10	9	70.2918	2.04E-07	6.889975	P200	P18	Ig kappa chain V-III region WOL OS=Homo sapiens PE=1 SV=1
P01625	5	2	36.8317	0.001011	4.681851	P200	P18	Ig kappa chain V-IV region Len OS=Homo sapiens PE=1 SV=2
P01714	2	2	21.0317	3.47E-05	20.09554	P200	P18	Ig lambda chain V-III region SH OS=Homo sapiens PE=1 SV=1
P01764	3	3	27.003	1.74E-05	12.85006	P200	P18	Ig heavy chain V-III region 23 OS=Homo sapiens GN=IGHV3-23 PE=1 SV=2
P01765;P01774;P01776;P01779	2	2	15.6066	0.042141	2.681199	P200	P18	Ig heavy chain V-III region TIL OS=Homo sapiens PE=1 SV=1
P01766;P01763;P01777	6	3	17.5492	1.16E-06	31.92404	P200	P18	Ig heavy chain V-III region BRO OS=Homo sapiens PE=1 SV=1
P01767	4	2	20.879	0.002173	14.09521	P200	P18	Ig heavy chain V-III region BUT OS=Homo sapiens PE=1 SV=1
P01781	3	3	12.2378	7.52E-05	4.604084	P200	P18	Ig heavy chain V-III region GAL OS=Homo sapiens PE=1 SV=1
P01833	43	42	408.384	8.13E-08	66.0165	P200	P18	Polymeric immunoglobulin receptor OS=Homo sapiens GN=PIGR PE=1 SV=4

P01834	28	27	162.8897	6.55E-07	6.55153	P200	P18	Ig kappa chain C region OS=Homo sapiens GN=IGKC PE=1 SV=1
P01857	23	7	197.2576	2.29E-05	10.78664	P200	P18	Ig gamma-1 chain C region OS=Homo sapiens GN=IGHG1 PE=1 SV=1
P01859	25	10	208.4535	2.29E-06	12.14798	P200	P18	Ig gamma-2 chain C region OS=Homo sapiens GN=IGHG2 PE=1 SV=2
P01861	16	2	95.9518	6.81E-07	20.608	P200	P18	Ig gamma-4 chain C region OS=Homo sapiens GN=IGHG4 PE=1 SV=1
P01871	19	7	166.8971	3.34E-06	66.70932	P200	P18	Ig mu chain C region OS=Homo sapiens GN=IGHM PE=1 SV=3
P01876	53	16	260.5912	3.00E-08	71.72799	P200	P18	Ig alpha-1 chain C region OS=Homo sapiens GN=IGHA1 PE=1 SV=2
P01877	43	7	214.8242	6.10E-06	62.6428	P200	P18	Ig alpha-2 chain C region OS=Homo sapiens GN=IGHA2 PE=1 SV=3
P02042	6	2	46.6321	3.58E-05	3.884764	P200	P18	Hemoglobin subunit delta OS=Homo sapiens GN=HBD PE=1 SV=2
P02511	8	8	43.0366	0.518736	1.045224	P200	P18	Alpha-crystallin B chain OS=Homo sapiens GN=CRYAB PE=1 SV=2
P02533	33	10	243.07	0.015837	1.289727	P200	P18	Keratin, type I cytoskeletal 14 OS=Homo sapiens GN=KRT14 PE=1 SV=4
P02538	32	4	255.4309	0.000107	5.477425	P200	P18	Keratin, type II cytoskeletal 6A OS=Homo sapiens GN=KRT6A PE=1 SV=3
P02649	7	7	39.8316	5.03E-05	4.605708	P200	P18	Apolipoprotein E OS=Homo sapiens GN=APOE PE=1 SV=1
P02671	22	19	240.7848	8.50E-08	14.07832	P200	P18	Fibrinogen alpha chain OS=Homo sapiens GN=FGA PE=1 SV=2
P02675	6	4	28.5459	5.21E-05	1.719405	P200	P18	Fibrinogen beta chain OS=Homo sapiens GN=FGB PE=1 SV=2
P02743	6	6	44.053	3.61E-05	5.961354	P200	P18	Serum amyloid P-component OS=Homo sapiens GN=APCS PE=1 SV=2
P02748	9	9	69.9697	5.50E-07	22.35174	P200	P18	Complement component C9 OS=Homo sapiens GN=C9 PE=1 SV=2
P02750	3	3	15.608	7.91E-07	92.20844	P200	P18	Leucine-rich alpha-2-glycoprotein OS=Homo sapiens GN=LRG1 PE=1 SV=2
P02760	33	31	317.4963	7.70E-06	2.684598	P200	P18	Protein AMBP OS=Homo sapiens GN=AMBP PE=1 SV=1
P02763	5	3	40.4521	9.92E-06	7.802911	P200	P18	Alpha-1-acid glycoprotein 1 OS=Homo sapiens GN=ORM1 PE=1 SV=1
P02765	9	9	60.8797	2.38E-05	2.885492	P200	P18	Alpha-2-HS-glycoprotein OS=Homo sapiens GN=AHSG PE=1 SV=1
P02768	134	131	1044.131	7.52E-08	12.29847	P200	P18	Serum albumin OS=Homo sapiens GN=ALB PE=1 SV=2

P02774	10	10	54.5631	0.00036	6.968565	P200	P18	Vitamin D-binding protein OS=Homo sapiens GN=GC PE=1 SV=1
P02787	39	33	367.2951	7.80E-08	10.06653	P200	P18	Serotransferrin OS=Homo sapiens GN=TF PE=1 SV=3
P02788	25	20	186.3586	3.20E-07	5.568543	P200	P18	Lactotransferrin OS=Homo sapiens GN=LTF PE=1 SV=6
P02790	16	15	125.4376	4.70E-06	5.999131	P200	P18	Hemopexin OS=Homo sapiens GN=HPX PE=1 SV=2
P02792	5	5	50.0657	1.58E-06	13.11795	P200	P18	Ferritin light chain OS=Homo sapiens GN=FTL PE=1 SV=2
P04004	13	13	90.6419	1.08E-06	5.587961	P200	P18	Vitronectin OS=Homo sapiens GN=VTN PE=1 SV=1
P04066	7	7	48.2905	2.83E-07	20.68492	P200	P18	Tissue alpha-L-fucosidase OS=Homo sapiens GN=FUCA1 PE=1 SV=4
P04075	12	7	79.9912	0.000155	1.688673	P200	P18	Fructose-bisphosphate aldolase A OS=Homo sapiens GN=ALDOA PE=1 SV=2
P04083	9	9	78.2592	0.001901	2.602809	P200	P18	Annexin A1 OS=Homo sapiens GN=ANXA1 PE=1 SV=2
P04208;P01700	2	2	12.6056	6.35E-05	8.30362	P200	P18	Ig lambda chain V-I region WAH OS=Homo sapiens PE=1 SV=1
P04216	5	5	38.057	0.000258	6.028326	P200	P18	Thy-1 membrane glycoprotein OS=Homo sapiens GN=THY1 PE=1 SV=2
P04217	12	11	102.8045	2.55E-06	10.50183	P200	P18	Alpha-1B-glycoprotein OS=Homo sapiens GN=A1BG PE=1 SV=4
P04220	15	2	130.9188	0.000446	24.30712	P200	P18	Ig mu heavy chain disease protein OS=Homo sapiens PE=1 SV=1
P04264	106	82	652.8242	0.000669	1.46322	P200	P18	Keratin, type II cytoskeletal 1 OS=Homo sapiens GN=KRT1 PE=1 SV=6
P04406	28	27	258.5456	0.004188	1.294621	P200	P18	Glyceraldehyde-3-phosphate dehydrogenase OS=Homo sapiens GN=GAPDH PE=1 SV=3
P04424	2	2	10.0118	7.96E-08	13.88368	P200	P18	Argininosuccinate lyase OS=Homo sapiens GN=ASL PE=1 SV=4
P04792	7	7	57.7551	0.444628	1.542928	P200	P18	Heat shock protein beta-1 OS=Homo sapiens GN=HSPB1 PE=1 SV=2
P05026	9	7	47.7125	0.129005	1.138538	P200	P18	Sodium/potassium-transporting ATPase subunit beta-1 OS=Homo sapiens GN=ATP1B1 PE=1 SV=1
P05090	40	40	267.2123	3.94E-06	3.638717	P200	P18	Apolipoprotein D OS=Homo sapiens GN=APOD PE=1 SV=1
P05109	7	7	52.5872	0.0762	1.599155	P200	P18	Protein S100-A8 OS=Homo sapiens GN=S100A8 PE=1 SV=1
P05154	31	29	211.2236	3.59E-06	6.168881	P200	P18	Plasma serine protease inhibitor OS=Homo

								sapiens GN=SERPINA5 PE=1 SV=3
P05155	21	20	171.1741	8.85E-07	14.01773	P200	P18	Plasma protease C1 inhibitor OS=Homo sapiens GN=SERPING1 PE=1 SV=2
P05543	16	14	98.7384	3.75E-07	47.39814	P200	P18	Thyroxine-binding globulin OS=Homo sapiens GN=SERPINA7 PE=1 SV=2
P06280	16	16	122.5313	9.66E-08	22.84375	P200	P18	Alpha-galactosidase A OS=Homo sapiens GN=GLA PE=1 SV=1
P06313;P06312;P06314	5	2	16.1539	6.02E-06	76.81545	P200	P18	Ig kappa chain V-IV region JI OS=Homo sapiens PE=4 SV=1
P06396	19	17	145.4181	0.000183	1.585856	P200	P18	Gelsolin OS=Homo sapiens GN=GSN PE=1 SV=1
P06702	13	13	84.4202	0.071402	1.11062	P200	P18	Protein S100-A9 OS=Homo sapiens GN=S100A9 PE=1 SV=1
P06744	14	12	109.0541	0.000589	1.691804	P200	P18	Glucose-6-phosphate isomerase OS=Homo sapiens GN=GPI PE=1 SV=4
P06865	5	5	30.616	8.84E-06	8.58325	P200	P18	Beta-hexosaminidase subunit alpha OS=Homo sapiens GN=HEXA PE=1 SV=2
P07288;P20151	6	6	42.8951	0.000185	2.952386	P200	P18	Prostate-specific antigen OS=Homo sapiens GN=KLK3 PE=1 SV=2
P07339	26	21	236.1479	8.16E-07	9.485753	P200	P18	Cathepsin D OS=Homo sapiens GN=CTSD PE=1 SV=1
P07355;A6NMY6	15	14	100.2629	6.10E-05	1.939443	P200	P18	Annexin A2 OS=Homo sapiens GN=ANXA2 PE=1 SV=2
P07602	25	25	168.1823	8.31E-08	8.002607	P200	P18	Prosaposin OS=Homo sapiens GN=PSAP PE=1 SV=2
P07686	6	5	32.7292	3.01E-05	10.53095	P200	P18	Beta-hexosaminidase subunit beta OS=Homo sapiens GN=HEXB PE=1 SV=3
P07711	5	5	26.6341	2.40E-05	6.032868	P200	P18	Cathepsin L1 OS=Homo sapiens GN=CTSL PE=1 SV=2
P07741	2	2	10.3693	0.028665	1.541093	P200	P18	Adenine phosphoribosyltransferase OS=Homo sapiens GN=APRT PE=1 SV=2
P07858	10	9	100.4036	3.22E-06	12.64882	P200	P18	Cathepsin B OS=Homo sapiens GN=CTSB PE=1 SV=3
P07900	16	9	100.1961	0.904344	1.005508	P200	P18	Heat shock protein HSP 90-alpha OS=Homo sapiens GN=HSP90AA1 PE=1 SV=5
P07996	10	8	55.6782	7.22E-05	15.22691	P200	P18	Thrombospondin-1 OS=Homo sapiens GN=THBS1 PE=1 SV=2
P08133	22	20	119.8548	0.002991	2.127551	P200	P18	Annexin A6 OS=Homo sapiens GN=ANXA6 PE=1 SV=3
P08183	22	20	126.705	0.025111	2.303951	P200	P18	Multidrug resistance protein 1 OS=Homo

								sapiens GN=ABCB1 PE=1 SV=3
P08195	10	10	67.3015	7.65E-06	3.115175	P200	P18	4F2 cell-surface antigen heavy chain OS=Homo sapiens GN=SLC3A2 PE=1 SV=3
P08236	12	12	66.504	1.86E-06	19.04476	P200	P18	Beta-glucuronidase OS=Homo sapiens GN=GUSB PE=1 SV=2
P08294	7	7	63.8498	2.06E-07	29.24462	P200	P18	Extracellular superoxide dismutase [Cu-Zn] OS=Homo sapiens GN=SOD3 PE=1 SV=2
P08473	46	42	398.5136	3.72E-05	2.126716	P200	P18	Neprilysin OS=Homo sapiens GN=MME PE=1 SV=2
P08571	8	7	70.0135	3.01E-05	17.04845	P200	P18	Monocyte differentiation antigen CD14 OS=Homo sapiens GN=CD14 PE=1 SV=2
P08582	17	16	140.9333	1.64E-07	4.349685	P200	P18	Melanotransferrin OS=Homo sapiens GN=MFI2 PE=1 SV=2
P08603	10	9	49.0807	0.017048	2.083174	P200	P18	Complement factor H OS=Homo sapiens GN=CFH PE=1 SV=4
P08727;P05783	16	3	99.4363	4.10E-05	5.595272	P200	P18	Keratin, type I cytoskeletal 19 OS=Homo sapiens GN=KRT19 PE=1 SV=4
P08754	12	4	64.5304	0.371658	1.20138	P200	P18	Guanine nucleotide-binding protein G(k) subunit alpha OS=Homo sapiens GN=GNAI3 PE=1 SV=3
P08758	14	12	91.9838	0.000721	1.939184	P200	P18	Annexin A5 OS=Homo sapiens GN=ANXA5 PE=1 SV=2
P08779	31	12	232.0221	7.28E-05	2.930811	P200	P18	Keratin, type I cytoskeletal 16 OS=Homo sapiens GN=KRT16 PE=1 SV=4
P09467	11	10	75.9964	0.072677	1.224135	P200	P18	Fructose-1,6-bisphosphatase 1 OS=Homo sapiens GN=FBP1 PE=1 SV=5
P09525	19	17	154.5272	0.152367	1.3062	P200	P18	Annexin A4 OS=Homo sapiens GN=ANXA4 PE=1 SV=4
P09543	7	6	46.3632	0.06683	1.137372	P200	P18	2',3'-cyclic-nucleotide 3'-phosphodiesterase OS=Homo sapiens GN=CNP PE=1 SV=2
P09668	5	5	30.6623	1.35E-05	10.06287	P200	P18	Pro-cathepsin H OS=Homo sapiens GN=CTSH PE=1 SV=4
P10153	7	7	53.9281	3.30E-05	6.210424	P200	P18	Non-secretory ribonuclease OS=Homo sapiens GN=RNASE2 PE=1 SV=2
P10253	25	25	195.4526	5.16E-08	23.73156	P200	P18	Lysosomal alpha-glucosidase OS=Homo sapiens GN=GAA PE=1 SV=4
P10619	39	37	314.8174	1.36E-07	48.36277	P200	P18	Lysosomal protective protein OS=Homo sapiens GN=CTSA PE=1 SV=2

P10909	23	22	223.0875	8.92E-08	17.99821	P200	P18	Clusterin OS=Homo sapiens GN=CLU PE=1 SV=1
P11117	13	13	88.1383	7.68E-06	10.10527	P200	P18	Lysosomal acid phosphatase OS=Homo sapiens GN=ACP2 PE=1 SV=3
P11142	22	11	169.7863	4.30E-06	5.968006	P200	P18	Heat shock cognate 71 kDa protein OS=Homo sapiens GN=HSPA8 PE=1 SV=1
P11279	5	4	39.4928	8.66E-06	5.071345	P200	P18	Lysosome-associated membrane glycoprotein 1 OS=Homo sapiens GN=LAMP1 PE=1 SV=3
P11586	8	7	45.437	0.013625	1.534251	P200	P18	C-1-tetrahydrofolate synthase, cytoplasmic OS=Homo sapiens GN=MTHFD1 PE=1 SV=3
P11597	5	5	27.642	6.68E-06	12.22551	P200	P18	Cholesteryl ester transfer protein OS=Homo sapiens GN=CETP PE=1 SV=2
P12109	12	9	102.2874	1.37E-05	4.383302	P200	P18	Collagen alpha-1(VI) chain OS=Homo sapiens GN=COL6A1 PE=1 SV=3
P12273	6	5	45.7716	6.53E-05	2.605522	P200	P18	Prolactin-inducible protein OS=Homo sapiens GN=PIP PE=1 SV=1
P12821	5	5	25.5696	0.00033	295.8467	P200	P18	Angiotensin-converting enzyme OS=Homo sapiens GN=ACE PE=1 SV=1
P12830	5	5	31.498	2.28E-05	25.26785	P200	P18	Cadherin-1 OS=Homo sapiens GN=CDH1 PE=1 SV=3
P13284	3	3	18.026	7.50E-08	Infinity	P200	P18	Gamma-interferon-inducible lysosomal thiol reductase OS=Homo sapiens GN=IFI30 PE=1 SV=3
P13473	2	2	11.703	9.29E-05	4.824592	P200	P18	Lysosome-associated membrane glycoprotein 2 OS=Homo sapiens GN=LAMP2 PE=1 SV=2
P13637	15	6	88.0667	2.26E-06	9.259453	P200	P18	Sodium/potassium-transporting ATPase subunit alpha-3 OS=Homo sapiens GN=ATP1A3 PE=1 SV=3
P13639	7	5	33.2321	0.011692	1.925674	P200	P18	Elongation factor 2 OS=Homo sapiens GN=EEF2 PE=1 SV=4
P13645;O76013;Q14525;Q15323;Q7Z3Y8;Q7Z3Z0	59	44	502.9591	0.006381	1.450892	P200	P18	Keratin, type I cytoskeletal 10 OS=Homo sapiens GN=KRT10 PE=1 SV=6
P13647	27	10	204.4754	1.20E-07	7.188967	P200	P18	Keratin, type II cytoskeletal 5 OS=Homo sapiens GN=KRT5 PE=1 SV=3
P13797	6	6	32.4061	0.112414	1.55436	P200	P18	Plastin-3 OS=Homo sapiens GN=PLS3 PE=1 SV=4
P13866	8	7	44.8587	0.000584	3.117142	P200	P18	Sodium/glucose cotransporter 1 OS=Homo

								sapiens GN=SLC5A1 PE=1 SV=1
P13987	11	11	87.8074	0.008253	1.731432	P200	P18	CD59 glycoprotein OS=Homo sapiens GN=CD59 PE=1 SV=1
P14174	4	4	35.7793	0.390817	1.027433	P200	P18	Macrophage migration inhibitory factor OS=Homo sapiens GN=MIF PE=1 SV=4
P14384	7	6	50.6242	0.000112	9.054351	P200	P18	Carboxypeptidase M OS=Homo sapiens GN=CPM PE=1 SV=2
P14410	6	3	42.9167	0.003766	331.1832	P200	P18	Sucrase-isomaltase, intestinal OS=Homo sapiens GN=SI PE=1 SV=6
P14543	14	12	113.3804	5.45E-06	8.40653	P200	P18	Nidogen-1 OS=Homo sapiens GN=NID1 PE=1 SV=3
P14618	22	22	161.8982	0.001653	2.036224	P200	P18	Pyruvate kinase PKM OS=Homo sapiens GN=PKM PE=1 SV=4
P14649	4	2	23.4318	0.001421	3.665722	P200	P18	Myosin light chain 6B OS=Homo sapiens GN=MYL6B PE=1 SV=1
P15121	3	3	20.6742	0.439997	1.066198	P200	P18	Aldose reductase OS=Homo sapiens GN=AKR1B1 PE=1 SV=3
P15144	97	91	792.9501	3.90E-06	2.301666	P200	P18	Aminopeptidase N OS=Homo sapiens GN=ANPEP PE=1 SV=4
P15289	10	10	82.9983	8.83E-06	16.96563	P200	P18	Arylsulfatase A OS=Homo sapiens GN=ARSA PE=1 SV=3
P15291	4	4	37.9875	1.47E-05	26.50991	P200	P18	Beta-1,4-galactosyltransferase 1 OS=Homo sapiens GN=B4GALT1 PE=1 SV=5
P15309	14	14	137.7349	1.16E-05	5.057093	P200	P18	Prostatic acid phosphatase OS=Homo sapiens GN=ACPP PE=1 SV=3
P15586	9	8	56.5535	2.09E-05	16.76327	P200	P18	N-acetylglucosamine-6-sulfatase OS=Homo sapiens GN=GNS PE=1 SV=3
P15848	7	7	37.1393	5.01E-06	16.31725	P200	P18	Arylsulfatase B OS=Homo sapiens GN=ARSB PE=1 SV=1
P15941	13	13	99.2991	1.63E-05	3.700735	P200	P18	Mucin-1 OS=Homo sapiens GN=MUC1 PE=1 SV=3
P16070	5	5	30.6787	1.36E-05	10.83166	P200	P18	CD44 antigen OS=Homo sapiens GN=CD44 PE=1 SV=3
P16152;O75828	7	6	67.3806	0.214868	1.194251	P200	P18	Carbonyl reductase [NADPH] 1 OS=Homo sapiens GN=CBR1 PE=1 SV=3
P16278;Q6UWU2	81	74	476.837	2.01E-08	30.43532	P200	P18	Beta-galactosidase OS=Homo sapiens GN=GLB1 PE=1 SV=2
P16444	37	36	352.9832	0.000226	1.61002	P200	P18	Dipeptidase 1 OS=Homo sapiens GN=DPEP1 PE=1 SV=3
P17050	3	2	16.5593	3.41E-06	97.91723	P200	P18	Alpha-N-acetylgalactosaminidase

								OS=Homo sapiens GN=NAGA PE=1 SV=2
P17066;P48741	12	6	82.0161	4.98E-07	6.204532	P200	P18	Heat shock 70 kDa protein 6 OS=Homo sapiens GN=HSPA6 PE=1 SV=2
P17900	5	5	52.6332	2.81E-05	5.530659	P200	P18	Ganglioside GM2 activator OS=Homo sapiens GN=GM2A PE=1 SV=4
P18669;P15259;Q8N0Y7	7	7	51.1569	0.000837	1.314457	P200	P18	Phosphoglycerate mutase 1 OS=Homo sapiens GN=PGAM1 PE=1 SV=2
P18827	2	2	12.1914	2.06E-05	4.914771	P200	P18	Syndecan-1 OS=Homo sapiens GN=SDC1 PE=1 SV=3
P19012	21	3	128.7873	0.001591	6.090997	P200	P18	Keratin, type I cytoskeletal 15 OS=Homo sapiens GN=KRT15 PE=1 SV=3
P19440;B5MD39;Q14390	29	2	178.3269	0.011426	2.072659	P200	P18	Gamma-glutamyltranspeptidase 1 OS=Homo sapiens GN=GGT1 PE=1 SV=2
P19652	3	2	18.5916	0.000233	4.762037	P200	P18	Alpha-1-acid glycoprotein 2 OS=Homo sapiens GN=ORM2 PE=1 SV=2
P19801	17	15	123.2943	1.52E-06	9.889772	P200	P18	Amiloride-sensitive amine oxidase [copper-containing] OS=Homo sapiens GN=AOC1 PE=1 SV=4
P19835	6	5	31.0134	1.49E-06	40.57259	P200	P18	Bile salt-activated lipase OS=Homo sapiens GN=CEL PE=1 SV=3
P20073	13	12	82.4013	1.55E-05	2.230243	P200	P18	Annexin A7 OS=Homo sapiens GN=ANXA7 PE=1 SV=3
P20336;O95716;Q96E17	8	3	50.086	7.41E-06	14.34786	P200	P18	Ras-related protein Rab-3A OS=Homo sapiens GN=RAB3A PE=1 SV=1
P20711	10	7	63.6064	0.000223	1.300239	P200	P18	Aromatic-L-amino-acid decarboxylase OS=Homo sapiens GN=DDC PE=1 SV=2
P21281	4	3	18.9759	0.810305	1.111274	P200	P18	V-type proton ATPase subunit B, brain isoform OS=Homo sapiens GN=ATP6V1B2 PE=1 SV=3
P21399	11	11	75.072	0.484204	1.08944	P200	P18	Cytoplasmic aconitate hydratase OS=Homo sapiens GN=ACO1 PE=1 SV=3
P21810	3	3	22.2664	4.91E-05	14.33661	P200	P18	Biglycan OS=Homo sapiens GN=BGN PE=1 SV=2
P21926	6	6	55.64	2.44E-06	27.80445	P200	P18	CD9 antigen OS=Homo sapiens GN=CD9 PE=1 SV=4
P22676	4	2	21.7458	1.66E-05	7.042069	P200	P18	Calretinin OS=Homo sapiens GN=CALB2 PE=2 SV=2
P22732	10	9	74.661	1.53E-06	3.242494	P200	P18	Solute carrier family 2, facilitated glucose transporter member 5 OS=Homo sapiens GN=SLC2A5 PE=1 SV=1

P22748	5	5	33.9117	3.08E-05	4.745778	P200	P18	Carbonic anhydrase 4 OS=Homo sapiens GN=CA4 PE=1 SV=2
P22792	18	18	150.2287	3.30E-07	37.68953	P200	P18	Carboxypeptidase N subunit 2 OS=Homo sapiens GN=CPN2 PE=1 SV=3
P22891	21	20	194.1994	1.15E-06	23.35703	P200	P18	Vitamin K-dependent protein Z OS=Homo sapiens GN=PROZ PE=1 SV=2
P23526	4	3	21.0215	8.03E-05	6.478994	P200	P18	Adenosylhomocysteinase OS=Homo sapiens GN=AHCY PE=1 SV=4
P25311	10	9	79.3116	0.001491	2.636637	P200	P18	Zinc-alpha-2-glycoprotein OS=Homo sapiens GN=AZGP1 PE=1 SV=2
P25325	6	6	43.5832	0.001251	6.148742	P200	P18	3-mercaptopyruvate sulfurtransferase OS=Homo sapiens GN=MPST PE=1 SV=3
P26038	46	21	305.4966	0.001545	1.341638	P200	P18	Moesin OS=Homo sapiens GN=MSN PE=1 SV=3
P27105	11	7	107.1394	1.84E-05	3.26884	P200	P18	Erythrocyte band 7 integral membrane protein OS=Homo sapiens GN=STOM PE=1 SV=3
P27487	58	49	423.8597	4.17E-06	3.154538	P200	P18	Dipeptidyl peptidase 4 OS=Homo sapiens GN=DPP4 PE=1 SV=2
P28066	5	4	33.7155	1.64E-05	4.363361	P200	P18	Proteasome subunit alpha type-5 OS=Homo sapiens GN=PSMA5 PE=1 SV=3
P28799	8	7	61.7112	2.39E-05	4.456694	P200	P18	Granulins OS=Homo sapiens GN=GRN PE=1 SV=2
P28838	10	10	46.4418	2.14E-05	8.088403	P200	P18	Cytosol aminopeptidase OS=Homo sapiens GN=LAP3 PE=1 SV=3
P29401	7	7	38.7843	6.87E-07	1.848942	P200	P18	Transketolase OS=Homo sapiens GN=TKT PE=1 SV=3
P29508	4	2	26.8532	3.08E-05	16.08756	P200	P18	Serpin B3 OS=Homo sapiens GN=SERPINB3 PE=1 SV=2
P29622	7	7	45.0049	1.90E-06	34.40564	P200	P18	Kallistatin OS=Homo sapiens GN=SERPINA4 PE=1 SV=3
P29972	5	5	44.7293	1.15E-05	2.454767	P200	P18	Aquaporin-1 OS=Homo sapiens GN=AQP1 PE=1 SV=3
P29992	8	6	42.0571	4.08E-07	19.74535	P200	P18	Guanine nucleotide-binding protein subunit alpha-11 OS=Homo sapiens GN=GNA11 PE=1 SV=2
P30039	3	3	25.1608	0.184804	1.25143	P200	P18	Phenazine biosynthesis-like domain-containing protein OS=Homo sapiens GN=PBLD PE=1 SV=2
P30046;A6NHG4	6	6	36.0772	0.063418	1.073213	P200	P18	D-dopachrome decarboxylase OS=Homo sapiens GN=DDT PE=1 SV=3
P30450;P10314;P16189;P18462;P30453;P3	7	2	37.1492	8.09E-08	7917.208	P200	P18	HLA class I histocompatibility antigen, A-26 alpha chain

0456;P304 57;P30459; P30512								OS=Homo sapiens GN=HLA-A PE=1 SV=2
P31150	13	6	71.8502	8.66E-06	5.170033	P200	P18	Rab GDP dissociation inhibitor alpha OS=Homo sapiens GN=GDI1 PE=1 SV=2
P31949	3	3	21.3438	0.014712	1.725228	P200	P18	Protein S100-A11 OS=Homo sapiens GN=S100A11 PE=1 SV=2
P32119	7	4	46.063	0.054641	1.224297	P200	P18	Peroxiredoxin-2 OS=Homo sapiens GN=PRDX2 PE=1 SV=5
P33908	27	26	226.4388	6.05E-07	8.540658	P200	P18	Mannosyl-oligosaccharide 1,2-alpha-mannosidase IA OS=Homo sapiens GN=MAN1A1 PE=1 SV=3
P34059	6	6	37.8783	1.57E-05	9.305278	P200	P18	N-acetylgalactosamine-6-sulfatase OS=Homo sapiens GN=GALNS PE=1 SV=1
P34896	11	10	72.8492	2.53E-06	4.797083	P200	P18	Serine hydroxymethyltransferase , cytosolic OS=Homo sapiens GN=SHMT1 PE=1 SV=1
P34931	8	2	53.451	0.000364	2.371074	P200	P18	Heat shock 70 kDa protein 1-like OS=Homo sapiens GN=HSPA1L PE=1 SV=2
P35527	75	67	623.3512	0.015935	1.174418	P200	P18	Keratin, type I cytoskeletal 9 OS=Homo sapiens GN=KRT9 PE=1 SV=3
P35858	4	3	30.3818	1.19E-05	206.5173	P200	P18	Insulin-like growth factor-binding protein complex acid labile subunit OS=Homo sapiens GN=IGFALS PE=1 SV=1
P35908	41	25	321.2351	0.004221	1.22014	P200	P18	Keratin, type II cytoskeletal 2 epidermal OS=Homo sapiens GN=KRT2 PE=1 SV=2
P36955	6	6	40.6385	3.11E-05	5.237654	P200	P18	Pigment epithelium-derived factor OS=Homo sapiens GN=SERPINF1 PE=1 SV=4
P36969	2	2	10.8496	0.000308	1.804303	P200	P18	Phospholipid hydroperoxide glutathione peroxidase, mitochondrial OS=Homo sapiens GN=GPX4 PE=1 SV=3
P38606	14	14	86.588	0.00029	1.459104	P200	P18	V-type proton ATPase catalytic subunit A OS=Homo sapiens GN=ATP6V1A PE=1 SV=2
P39059	13	12	91.1814	1.04E-07	10.36414	P200	P18	Collagen alpha-1(XV) chain OS=Homo sapiens GN=COL15A1 PE=1 SV=2
P40121	4	3	18.9347	0.00026	3.036086	P200	P18	Macrophage-capping protein OS=Homo sapiens GN=CAPG PE=1 SV=2
P41181	6	6	59.5104	3.18E-05	3.869706	P200	P18	Aquaporin-2 OS=Homo sapiens GN=AQP2 PE=1 SV=1

P41222	11	11	128.2113	8.26E-06	2.207256	P200	P18	Prostaglandin-H2 D-isomerase OS=Homo sapiens GN=PTGDS PE=1 SV=1
P42685	5	4	25.0448	0.022527	2.006215	P200	P18	Tyrosine-protein kinase FRK OS=Homo sapiens GN=FRK PE=1 SV=1
P42785	7	6	67.4978	6.20E-07	9.701791	P200	P18	Lysosomal Pro-X carboxypeptidase OS=Homo sapiens GN=PRCP PE=1 SV=1
P43251	5	5	50.4867	8.91E-06	91.30139	P200	P18	Biotinidase OS=Homo sapiens GN=BTD PE=1 SV=2
P45880	5	5	34.4512	0.000127	1.968594	P200	P18	Voltage-dependent anion-selective channel protein 2 OS=Homo sapiens GN=VDAC2 PE=1 SV=2
P49221	23	18	156.353	0.034458	1.425862	P200	P18	Protein-glutamine gamma-glutamyltransferase 4 OS=Homo sapiens GN=TGM4 PE=1 SV=2
P50148	5	3	31.2434	0.927472	1.357363	P200	P18	Guanine nucleotide-binding protein G(q) subunit alpha OS=Homo sapiens GN=GNAQ PE=1 SV=4
P50395	15	8	95.926	0.056548	1.338652	P200	P18	Rab GDP dissociation inhibitor beta OS=Homo sapiens GN=GDI2 PE=1 SV=2
P50440	5	3	29.2151	0.029617	1.273994	P200	P18	Glycine amidinotransferase, mitochondrial OS=Homo sapiens GN=GATM PE=1 SV=1
P50897	2	2	12.8964	0.000495	2.818188	P200	P18	Palmitoyl-protein thioesterase 1 OS=Homo sapiens GN=PPT1 PE=1 SV=1
P50993	13	5	76.9761	1.61E-05	4.703669	P200	P18	Sodium/potassium-transporting ATPase subunit alpha-2 OS=Homo sapiens GN=ATP1A2 PE=1 SV=1
P50995	20	19	171.0912	5.04E-06	3.433635	P200	P18	Annexin A11 OS=Homo sapiens GN=ANXA11 PE=1 SV=1
P51149	12	9	58.4807	4.01E-05	2.994096	P200	P18	Ras-related protein Rab-7a OS=Homo sapiens GN=RAB7A PE=1 SV=1
P51654	4	4	28.7512	2.20E-06	48.50266	P200	P18	Glypican-3 OS=Homo sapiens GN=GPC3 PE=1 SV=1
P51688	3	3	15.2574	0.870027	1.028908	P200	P18	N-sulphoglucosamine sulphohydrolase OS=Homo sapiens GN=SGSH PE=1 SV=1
P52209	3	2	18.2032	0.7734	1.011757	P200	P18	6-phosphogluconate dehydrogenase, decarboxylating OS=Homo sapiens GN=PGD PE=1 SV=3
P52565	4	4	33.7029	0.196888	1.244884	P200	P18	Rho GDP-dissociation inhibitor 1 OS=Homo sapiens GN=ARHGDIA PE=1 SV=3

P52788	4	4	22.1553	2.92E-07	57.86301	P200	P18	Spermine synthase OS=Homo sapiens GN=SMS PE=1 SV=2
P52907	4	2	21.5777	0.118417	1.179864	P200	P18	F-actin-capping protein subunit alpha-1 OS=Homo sapiens GN=CAPZA1 PE=1 SV=3
P53634	28	27	277.5294	1.88E-07	19.03531	P200	P18	Dipeptidyl peptidase 1 OS=Homo sapiens GN=CTSC PE=1 SV=2
P53801	4	4	21.929	0.000468	2.947583	P200	P18	Pituitary tumor-transforming gene 1 protein-interacting protein OS=Homo sapiens GN=PTTG1IP PE=1 SV=1
P53990	19	19	191.0223	2.67E-07	8.189352	P200	P18	IST1 homolog OS=Homo sapiens GN=IST1 PE=1 SV=1
P54760	14	11	80.6623	0.000277	18.64368	P200	P18	Ephrin type-B receptor 4 OS=Homo sapiens GN=EPHB4 PE=1 SV=2
P54793	12	8	83.4175	0.000764	1.519606	P200	P18	Arylsulfatase F OS=Homo sapiens GN=ARSF PE=1 SV=4
P54802	60	58	379.89	5.46E-08	55.78374	P200	P18	Alpha-N-acetylglucosaminidase OS=Homo sapiens GN=NAGLU PE=1 SV=2
P54803	10	10	57.482	0.000268	21.21264	P200	P18	Galactocerebrosidase OS=Homo sapiens GN=GALC PE=1 SV=2
P54920	4	4	19.005	0.273804	1.152185	P200	P18	Alpha-soluble NSF attachment protein OS=Homo sapiens GN=NAPA PE=1 SV=3
P55017	6	6	39.1617	0.00149	13.53892	P200	P18	Solute carrier family 12 member 3 OS=Homo sapiens GN=SLC12A3 PE=1 SV=3
P55072	11	11	57.3394	0.00108	2.404294	P200	P18	Transitional endoplasmic reticulum ATPase OS=Homo sapiens GN=VCP PE=1 SV=4
P55083	2	2	27.2182	5.81E-07	20.5331	P200	P18	Microfibril-associated glycoprotein 4 OS=Homo sapiens GN=MFAP4 PE=1 SV=2
P55287	6	5	31.0429	0.000201	27.14618	P200	P18	Cadherin-11 OS=Homo sapiens GN=CDH11 PE=2 SV=2
P55291	5	5	27.0376	0.102261	1.07996	P200	P18	Cadherin-15 OS=Homo sapiens GN=CDH15 PE=1 SV=1
P56537	4	3	26.0177	0.002222	15.74918	P200	P18	Eukaryotic translation initiation factor 6 OS=Homo sapiens GN=EIF6 PE=1 SV=1
P59665;P59666	3	3	22.5716	0.614038	1.02773	P200	P18	Neutrophil defensin 1 OS=Homo sapiens GN=DEFA1 PE=1 SV=1
P60033	4	3	29.6184	1.12E-06	39.7995	P200	P18	CD81 antigen OS=Homo sapiens GN=CD81 PE=1 SV=1
P60900	3	3	17.3391	0.001853	3.932214	P200	P18	Proteasome subunit alpha type-6 OS=Homo sapiens GN=PSMA6 PE=1 SV=1

P60953	4	4	39.1758	0.008596	1.297947	P200	P18	Cell division control protein 42 homolog OS=Homo sapiens GN=CDC42 PE=1 SV=2
P61026	7	4	40.2569	0.000212	1.932018	P200	P18	Ras-related protein Rab-10 OS=Homo sapiens GN=RAB10 PE=1 SV=1
P61586;P62745	6	3	30.5241	0.026464	1.847064	P200	P18	Transforming protein RhoA OS=Homo sapiens GN=RHOA PE=1 SV=1
P61769	4	4	39.1086	0.568235	1.060841	P200	P18	Beta-2-microglobulin OS=Homo sapiens GN=B2M PE=1 SV=1
P61970	9	8	80.625	5.22E-06	22.1558	P200	P18	Nuclear transport factor 2 OS=Homo sapiens GN=NUTF2 PE=1 SV=1
P62258	11	8	70.2134	0.146544	1.192897	P200	P18	14-3-3 protein epsilon OS=Homo sapiens GN=YWHAE PE=1 SV=1
P62491;Q15907	6	6	36.3635	0.372078	1.165687	P200	P18	Ras-related protein Rab-11A OS=Homo sapiens GN=RAB11A PE=1 SV=3
P62805	3	2	18.7617	0.002282	1.222301	P200	P18	Histone H4 OS=Homo sapiens GN=HIST1H4A PE=1 SV=2
P62834	8	3	47.3691	8.98E-06	6.903057	P200	P18	Ras-related protein Rap-1A OS=Homo sapiens GN=RAP1A PE=1 SV=1
P62873	11	6	83.9318	0.000228	3.384458	P200	P18	Guanine nucleotide-binding protein G(I)/G(S)/G(T) subunit beta-1 OS=Homo sapiens GN=GNB1 PE=1 SV=3
P62879;Q9HAV0	9	5	78.3833	0.000787	3.117567	P200	P18	Guanine nucleotide-binding protein G(I)/G(S)/G(T) subunit beta-2 OS=Homo sapiens GN=GNB2 PE=1 SV=3
P62937	18	14	159.9164	0.005165	1.236743	P200	P18	Peptidyl-prolyl cis-trans isomerase A OS=Homo sapiens GN=PPIA PE=1 SV=2
P62942	2	2	9.8641	0.022517	1.272862	P200	P18	Peptidyl-prolyl cis-trans isomerase FKBP1A OS=Homo sapiens GN=FKBP1A PE=1 SV=2
P62979;P0CG47;P0CG48;P62987	14	13	83.7417	0.000561	1.993134	P200	P18	Ubiquitin-40S ribosomal protein S27a OS=Homo sapiens GN=RPS27A PE=1 SV=2
P63000;P15153;P60763	6	6	53.5013	0.022698	1.979591	P200	P18	Ras-related C3 botulinum toxin substrate 1 OS=Homo sapiens GN=RAC1 PE=1 SV=1
P63092;P19087;Q5JWF2	16	9	84.7771	0.013572	1.776715	P200	P18	Guanine nucleotide-binding protein G(s) subunit alpha isoforms short OS=Homo sapiens GN=GNAS PE=1 SV=1
P63104;P31947;Q04917	11	7	72.3562	0.000223	1.866048	P200	P18	14-3-3 protein zeta/delta OS=Homo sapiens GN=YWHAZ PE=1 SV=1
P68371;Q9BUF5	14	2	98.2057	3.52E-07	21.76106	P200	P18	Tubulin beta-4B chain OS=Homo sapiens

								GN=TUBB4B PE=1 SV=1
P68871;P02100	10	6	70.1368	0.044426	1.229916	P200	P18	Hemoglobin subunit beta OS=Homo sapiens GN=HBB PE=1 SV=2
P69905	4	2	29.2166	0.000845	2.258721	P200	P18	Hemoglobin subunit alpha OS=Homo sapiens GN=HBA1 PE=1 SV=2
P80723	11	10	79.7551	0.011975	1.477995	P200	P18	Brain acid soluble protein 1 OS=Homo sapiens GN=BASP1 PE=1 SV=2
P80748	2	2	7.2329	0.001472	32.61917	P200	P18	Ig lambda chain V-III region LOI OS=Homo sapiens PE=1 SV=1
P81605	4	4	48.0403	0.000716	1.560605	P200	P18	Dermcidin OS=Homo sapiens GN=DCD PE=1 SV=2
P84077;P61204;P84085	11	6	84.6175	0.003261	1.302951	P200	P18	ADP-ribosylation factor 1 OS=Homo sapiens GN=ARF1 PE=1 SV=2
P84095	4	4	19.6204	0.327138	2.015312	P200	P18	Rho-related GTP-binding protein RhoG OS=Homo sapiens GN=RHOG PE=1 SV=1
Q1EHB4	6	6	39.4753	0.001166	1.999333	P200	P18	Sodium-coupled monocarboxylate transporter 2 OS=Homo sapiens GN=SLC5A12 PE=2 SV=2
Q3LXA3	17	17	124.6095	0.029173	1.530417	P200	P18	Triokinase/FMN cyclase OS=Homo sapiens GN=TKFC PE=1 SV=2
Q5VW32	15	15	135.9	3.93E-06	9.694016	P200	P18	BRO1 domain-containing protein BROX OS=Homo sapiens GN=BROX PE=1 SV=1
Q6EMK4	6	3	35.7479	1.11E-05	5.015569	P200	P18	Vasorin OS=Homo sapiens GN=VASN PE=1 SV=1
Q6P9A2	3	3	17.5316	2.70E-05	24.72613	P200	P18	Polypeptide N-acetylgalactosaminyltransferase 18 OS=Homo sapiens GN=GALNT18 PE=2 SV=2
Q6UWR7	5	3	32.5221	0.388032	1.266668	P200	P18	Ectonucleotide pyrophosphatase/phosphodiesterase family member 6 OS=Homo sapiens GN=ENPP6 PE=1 SV=2
Q6UX06	48	44	369.0378	9.87E-07	7.030823	P200	P18	Olfactomedin-4 OS=Homo sapiens GN=OLFM4 PE=1 SV=1
Q6ZMV7	3	3	17.8972	0.000981	3.776714	P200	P18	Leucine-, glutamate- and lysine-rich protein 1 OS=Homo sapiens GN=LEKR1 PE=2 SV=2
Q7L5L3	4	3	26.0323	5.27E-05	3.718164	P200	P18	Glycerophosphodiester phosphodiesterase domain-containing protein 3 OS=Homo sapiens GN=GDPD3 PE=2 SV=3
Q7L273	5	3	24.3942	0.024449	1.621279	P200	P18	BTB/POZ domain-containing protein KCTD9 OS=Homo sapiens GN=KCTD9 PE=1 SV=1
Q7LBR1	6	4	45.7289	3.66E-05	12.15235	P200	P18	Charged multivesicular body protein 1b OS=Homo sapiens

								GN=CHMP1B PE=1 SV=1
Q7Z3Y9	6	4	40.9057	0.012476	5.30957	P200	P18	Keratin, type I cytoskeletal 26 OS=Homo sapiens GN=KRT26 PE=1 SV=2
Q7Z4W1	6	6	28.6921	0.000218	1.686449	P200	P18	L-xylulose reductase OS=Homo sapiens GN=DCXR PE=1 SV=2
Q7Z5L0	11	10	83.4707	3.60E-06	4.914007	P200	P18	Vitelline membrane outer layer protein 1 homolog OS=Homo sapiens GN=VMO1 PE=1 SV=1
Q7Z794	10	4	68.632	0.241473	1.236528	P200	P18	Keratin, type II cytoskeletal 1b OS=Homo sapiens GN=KRT77 PE=2 SV=3
Q8IWA5	2	2	10.9972	5.74E-07	10.6938	P200	P18	Choline transporter-like protein 2 OS=Homo sapiens GN=SLC44A2 PE=1 SV=3
Q8IX04	3	3	24.673	2.53E-05	3.870822	P200	P18	Ubiquitin-conjugating enzyme E2 variant 3 OS=Homo sapiens GN=UEVLD PE=1 SV=2
Q8IXS6	5	5	35.2264	5.89E-05	3.20493	P200	P18	Paralemmin-2 OS=Homo sapiens GN=PALM2 PE=1 SV=3
Q8N1E6	2	2	11.359	1.19E-05	185.0304	P200	P18	F-box/LRR-repeat protein 14 OS=Homo sapiens GN=FBXL14 PE=1 SV=1
Q8N2U0	2	2	24.2624	0.478239	1.0737	P200	P18	Transmembrane protein 256 OS=Homo sapiens GN=TMEM256 PE=3 SV=1
Q8N6Q3	2	2	10.769	2.51E-05	9.758901	P200	P18	CD177 antigen OS=Homo sapiens GN=CD177 PE=1 SV=2
Q8N335	4	2	23.7421	1.48E-05	19.88382	P200	P18	Glycerol-3-phosphate dehydrogenase 1-like protein OS=Homo sapiens GN=GPD1L PE=1 SV=1
Q8NCE2	3	2	15.6393	0.014146	3.036839	P200	P18	Myotubularin-related protein 14 OS=Homo sapiens GN=MTMR14 PE=1 SV=2
Q8NHP1;O43488	5	2	27.2412	0.007627	1.385529	P200	P18	Aflatoxin B1 aldehyde reductase member 4 OS=Homo sapiens GN=AKR7L PE=2 SV=6
Q8TAA3	2	2	9.3248	0.003093	2.671865	P200	P18	Proteasome subunit alpha type-7-like OS=Homo sapiens GN=PSMA8 PE=2 SV=3
Q8TF65	3	2	15.1131	0.000243	6.780788	P200	P18	PDZ domain-containing protein GIPC2 OS=Homo sapiens GN=GIPC2 PE=1 SV=1
Q8WUM4	50	47	411.6954	8.98E-07	5.498834	P200	P18	Programmed cell death 6-interacting protein OS=Homo sapiens GN=PDCD6IP PE=1 SV=1
Q8WV92	4	3	29.8848	2.04E-05	10.86231	P200	P18	MIT domain-containing protein 1 OS=Homo sapiens GN=MITD1 PE=1 SV=1
Q8WVN6	7	7	73.2873	4.98E-05	2.743081	P200	P18	Secreted and transmembrane protein 1

								OS=Homo sapiens GN=SECTM1 PE=1 SV=2
Q8WW52	7	6	32.8774	0.938106	1.130674	P200	P18	Protein FAM151A OS=Homo sapiens GN=FAM151A PE=2 SV=2
Q8WWA0	5	5	39.1878	1.95E-06	12.38696	P200	P18	Intelectin-1 OS=Homo sapiens GN=ITLN1 PE=1 SV=1
Q8WZ75	4	3	29.1506	0.002696	21.33365	P200	P18	Roundabout homolog 4 OS=Homo sapiens GN=ROBO4 PE=1 SV=1
Q9BRA2	4	4	22.2976	0.200841	1.217641	P200	P18	Thioredoxin domain-containing protein 17 OS=Homo sapiens GN=TXNDC17 PE=1 SV=1
Q9BRK3	27	23	179.9613	6.80E-07	8.398517	P200	P18	Matrix-remodeling-associated protein 8 OS=Homo sapiens GN=MXRA8 PE=1 SV=1
Q9BRK5	2	2	9.743	2.88E-06	9.097085	P200	P18	45 kDa calcium-binding protein OS=Homo sapiens GN=SDF4 PE=1 SV=1
Q9BTY2	3	3	18.2967	6.88E-08	32.55599	P200	P18	Plasma alpha-L-fucosidase OS=Homo sapiens GN=FUCA2 PE=1 SV=2
Q9BUT1	6	5	39.1591	0.418602	1.035383	P200	P18	3-hydroxybutyrate dehydrogenase type 2 OS=Homo sapiens GN=BDH2 PE=1 SV=2
Q9BVA1; Q13885	9	2	66.2093	0.052922	1.089896	P200	P18	Tubulin beta-2B chain OS=Homo sapiens GN=TUBB2B PE=1 SV=1
Q9BVJ6	7	6	45.4835	3.89E-05	6.595089	P200	P18	U3 small nucleolar RNA-associated protein 14 homolog A OS=Homo sapiens GN=UTP14A PE=1 SV=1
Q9BVM4	4	4	26.257	0.033492	1.432477	P200	P18	Gamma-glutamylaminecyclotransferase OS=Homo sapiens GN=GGACT PE=1 SV=2
Q9BXP8	25	23	186.1222	2.38E-05	5.480209	P200	P18	Pappalysin-2 OS=Homo sapiens GN=PAPPA2 PE=1 SV=4
Q9BXU9	2	2	10.0356	5.83E-06	98.87725	P200	P18	Calcium-binding protein 8 OS=Homo sapiens GN=CALN1 PE=2 SV=1
Q9BYE9	8	7	46.2035	3.89E-08	22.75491	P200	P18	Cadherin-related family member 2 OS=Homo sapiens GN=CDHR2 PE=1 SV=2
Q9BYF1	17	16	109.3497	1.45E-06	2.30208	P200	P18	Angiotensin-converting enzyme 2 OS=Homo sapiens GN=ACE2 PE=1 SV=2
Q9C0H2	4	4	36.9981	0.001033	1.738616	P200	P18	Protein tweety homolog 3 OS=Homo sapiens GN=TTYH3 PE=1 SV=3
Q9H0E2	2	2	10.3545	0.199362	1.408048	P200	P18	Toll-interacting protein OS=Homo sapiens GN=TOLLIP PE=1 SV=1
Q9H3G5	29	29	187.8471	1.01E-06	13.32813	P200	P18	Probable serine carboxypeptidase CPVL

								OS=Homo sapiens GN=CPVL PE=1 SV=2
Q9H3R2	4	2	26.5797	1.30E-05	24.83739	P200	P18	Mucin-13 OS=Homo sapiens GN=MUC13 PE=1 SV=3
Q9H4M9	5	5	28.6547	0.000463	3.637154	P200	P18	EH domain-containing protein 1 OS=Homo sapiens GN=EHD1 PE=1 SV=2
Q9H6X2	6	6	33.9507	5.25E-08	40.38128	P200	P18	Anthrax toxin receptor 1 OS=Homo sapiens GN=ANTXR1 PE=1 SV=2
Q9H9H4	2	2	10.1845	3.01E-05	21.58943	P200	P18	Vacuolar protein sorting-associated protein 37B OS=Homo sapiens GN=VPS37B PE=1 SV=1
Q9H9S4	2	2	10.7087	0.066074	1.337188	P200	P18	Calcium-binding protein 39-like OS=Homo sapiens GN=CAB39L PE=1 SV=3
Q9H223	7	5	35.6488	0.000512	1.76567	P200	P18	EH domain-containing protein 4 OS=Homo sapiens GN=EHD4 PE=1 SV=1
Q9H444	5	5	29.1603	0.00099	4.445501	P200	P18	Charged multivesicular body protein 4b OS=Homo sapiens GN=CHMP4B PE=1 SV=1
Q9HB40	12	12	86.8	6.99E-06	36.00293	P200	P18	Retinoid-inducible serine carboxypeptidase OS=Homo sapiens GN=SCPEP1 PE=1 SV=1
Q9HD89	3	3	22.1544	9.89E-06	9.105509	P200	P18	Resistin OS=Homo sapiens GN=RETN PE=1 SV=1
Q9NP79	9	9	63.2965	1.22E-05	4.47678	P200	P18	Vacuolar protein sorting-associated protein VTA1 homolog OS=Homo sapiens GN=VTA1 PE=1 SV=1
Q9NP85	2	2	11.7496	1.85E-05	3.551944	P200	P18	Podocin OS=Homo sapiens GN=NPHS2 PE=1 SV=1
Q9NQ84	13	13	100.4279	2.19E-06	11.73683	P200	P18	G-protein coupled receptor family C group 5 member C OS=Homo sapiens GN=GPRC5C PE=1 SV=2
Q9NQR4	4	4	21.3341	0.0979	1.199812	P200	P18	Omega-amidase NIT2 OS=Homo sapiens GN=NIT2 PE=1 SV=1
Q9NRW1	3	2	19.1891	0.048765	12.15422	P200	P18	Ras-related protein Rab-6B OS=Homo sapiens GN=RAB6B PE=1 SV=1
Q9NS93	2	2	11.9208	1.88E-08	Infinity	P200	P18	Transmembrane 7 superfamily member 3 OS=Homo sapiens GN=TM7SF3 PE=2 SV=1
Q9NXU5	2	2	12.3263	5.74E-06	10.14262	P200	P18	ADP-ribosylation factor-like protein 15 OS=Homo sapiens GN=ARL15 PE=1 SV=1
Q9NZH0	8	8	60.503	8.92E-07	15.71584	P200	P18	G-protein coupled receptor family C group 5 member B OS=Homo

								sapiens GN=GPRC5B PE=2 SV=2
Q9NZP8	4	4	23.5962	1.03E-06	14.15875	P200	P18	Complement C1r subcomponent-like protein OS=Homo sapiens GN=C1RL PE=1 SV=2
Q9NZT1	5	4	47.467	7.00E-06	14.1047	P200	P18	Calmodulin-like protein 5 OS=Homo sapiens GN=CALML5 PE=1 SV=2
Q9NZZ3	9	8	77.0558	5.60E-07	4.677916	P200	P18	Charged multivesicular body protein 5 OS=Homo sapiens GN=CHMP5 PE=1 SV=1
Q9UBD6	3	3	30.3648	4.38E-07	34.83127	P200	P18	Ammonium transporter Rh type C OS=Homo sapiens GN=RHCG PE=1 SV=1
Q9UBQ7	3	3	17.6153	0.95009	1.086345	P200	P18	Glyoxylate reductase/hydroxypyruvate reductase OS=Homo sapiens GN=GRHPR PE=1 SV=1
Q9UBR2	7	6	43.781	0.004603	1.558716	P200	P18	Cathepsin Z OS=Homo sapiens GN=CTSZ PE=1 SV=1
Q9UGT4	17	16	151.0337	1.63E-07	11.48119	P200	P18	Sushi domain-containing protein 2 OS=Homo sapiens GN=SUSD2 PE=1 SV=1
Q9UHG3	5	3	33.6496	0.000837	5.948857	P200	P18	Prenylcysteine oxidase 1 OS=Homo sapiens GN=PCYOX1 PE=1 SV=3
Q9UHI7	11	9	72.0423	0.044598	1.809244	P200	P18	Solute carrier family 23 member 1 OS=Homo sapiens GN=SLC23A1 PE=1 SV=3
Q9UHL4	2	2	12.1235	0.000147	18.72058	P200	P18	Dipeptidyl peptidase 2 OS=Homo sapiens GN=DPP7 PE=1 SV=3
Q9UK41	7	7	57.4677	2.00E-06	9.484376	P200	P18	Vacuolar protein sorting-associated protein 28 homolog OS=Homo sapiens GN=VPS28 PE=1 SV=1
Q9UKB1	2	2	9.9855	0.010307	6.192746	P200	P18	F-box/WD repeat-containing protein 11 OS=Homo sapiens GN=FBXW11 PE=1 SV=1
Q9UKL6	3	2	17.2775	5.08E-06	24.01343	P200	P18	Phosphatidylcholine transfer protein OS=Homo sapiens GN=PCTP PE=1 SV=1
Q9UKU6	11	10	68.0651	4.78E-07	17.31451	P200	P18	Thyrotropin-releasing hormone-degrading ectoenzyme OS=Homo sapiens GN=TRHDE PE=2 SV=1
Q9UKU9	38	34	217.1222	3.04E-06	8.997857	P200	P18	Angiopoietin-related protein 2 OS=Homo sapiens GN=ANGPTL2 PE=2 SV=1
Q9UN37	15	10	81.8276	4.94E-06	5.299933	P200	P18	Vacuolar protein sorting-associated protein 4A OS=Homo sapiens GN=VPS4A PE=1 SV=1

Q9UNH7	4	4	34.3219	0.000294	11.36807	P200	P18	Sorting nexin-6 OS=Homo sapiens GN=SNX6 PE=1 SV=1
Q9UNN8	3	3	22.0446	0.000103	13.88212	P200	P18	Endothelial protein C receptor OS=Homo sapiens GN=PROCR PE=1 SV=1
Q9UQB8	9	8	48.5989	0.001555	1.621644	P200	P18	Brain-specific angiogenesis inhibitor 1-associated protein 2 OS=Homo sapiens GN=BAIAP2 PE=1 SV=1
Q9UQN3	6	6	28.8521	0.018853	1.899887	P200	P18	Charged multivesicular body protein 2b OS=Homo sapiens GN=CHMP2B PE=1 SV=1
Q9Y2E5	9	9	52.5501	8.26E-05	8.258085	P200	P18	Epididymis-specific alpha-mannosidase OS=Homo sapiens GN=MAN2B2 PE=1 SV=4
Q9Y2S2	9	8	69.7873	0.000325	1.368218	P200	P18	Lambda-crystallin homolog OS=Homo sapiens GN=CRYL1 PE=1 SV=3
Q9Y4C0	2	2	10.2307	0.000912	80.23606	P200	P18	Neurexin-3 OS=Homo sapiens GN=NRXN3 PE=1 SV=4
Q9Y5L4	3	3	17.458	1.09E-06	7.675958	P200	P18	Mitochondrial import inner membrane translocase subunit Tim13 OS=Homo sapiens GN=TIMM13 PE=1 SV=1
Q9Y6R1	8	5	37.4783	0.001257	2.943905	P200	P18	Electrogenic sodium bicarbonate cotransporter 1 OS=Homo sapiens GN=SLC4A4 PE=1 SV=1
Q9Y6W3	21	19	147.2919	0.000192	2.936056	P200	P18	Calpain-7 OS=Homo sapiens GN=CAPN7 PE=1 SV=1
Q9Y274	3	3	12.0798	4.26E-05	2.046658	P200	P18	Type 2 lactosamine alpha-2,3-sialyltransferase OS=Homo sapiens GN=ST3GAL6 PE=1 SV=1
Q9Y277	4	3	22.9304	0.849462	1.072709	P200	P18	Voltage-dependent anion-selective channel protein 3 OS=Homo sapiens GN=VDAC3 PE=1 SV=1
Q9Y617	2	2	10.6811	0.373403	1.123012	P200	P18	Phosphoserine aminotransferase OS=Homo sapiens GN=PSAT1 PE=1 SV=2
Q9Y646	7	5	54.3895	1.86E-06	49.21583	P200	P18	Carboxypeptidase Q OS=Homo sapiens GN=CPQ PE=1 SV=1
Q58FF8	9	2	54.7543	3.64E-05	18.03858	P200	P18	Putative heat shock protein HSP 90-beta 2 OS=Homo sapiens GN=HSP90AB2P PE=1 SV=2
Q86T13	4	4	22.9638	0.00012	15.95892	P200	P18	C-type lectin domain family 14 member A OS=Homo sapiens GN=CLEC14A PE=1 SV=1

Q86YQ8	6	5	35.9673	7.29E-05	8.010783	P200	P18	Copine-8 OS=Homo sapiens GN=CPNE8 PE=1 SV=2
Q96BM9	2	2	9.0837	0.034987	1.181299	P200	P18	ADP-ribosylation factor-like protein 8A OS=Homo sapiens GN=ARL8A PE=1 SV=1
Q96DA0	7	7	59.6254	8.68E-07	11.67571	P200	P18	Zymogen granule protein 16 homolog B OS=Homo sapiens GN=ZG16B PE=1 SV=3
Q96DA2	4	2	19.4876	0.323622	1.057466	P200	P18	Ras-related protein Rab-39B OS=Homo sapiens GN=RAB39B PE=1 SV=1
Q96DG6	11	9	58.6135	6.90E-06	2.706031	P200	P18	Carboxymethylenebutenolidase homolog OS=Homo sapiens GN=CMBL PE=1 SV=1
Q96EY5	3	3	15.0554	3.20E-05	6.996069	P200	P18	Multivesicular body subunit 12A OS=Homo sapiens GN=MVB12A PE=1 SV=1
Q96IU4	10	9	72.2783	1.63E-05	3.123812	P200	P18	Protein ABHD14B OS=Homo sapiens GN=ABHD14B PE=1 SV=1
Q96PD5	9	9	56.0843	3.05E-06	6.163104	P200	P18	N-acetylmuramoyl-L-alanine amidase OS=Homo sapiens GN=PGLYRP2 PE=1 SV=1
Q96QR8	4	4	22.6674	0.000167	46.11299	P200	P18	Transcriptional activator protein Pur-beta OS=Homo sapiens GN=PURB PE=1 SV=3
Q562R1	26	4	168.8377	0.000294	2.985976	P200	P18	Beta-actin-like protein 2 OS=Homo sapiens GN=ACTBL2 PE=1 SV=2
Q00796	9	9	89.6177	0.016877	1.515628	P200	P18	Sorbitol dehydrogenase OS=Homo sapiens GN=SORD PE=1 SV=4
Q01518	8	6	37.2331	0.104455	1.671429	P200	P18	Adenylyl cyclase-associated protein 1 OS=Homo sapiens GN=CAP1 PE=1 SV=5
Q01546	11	3	84.52	0.001748	1.368069	P200	P18	Keratin, type II cytoskeletal 2 oral OS=Homo sapiens GN=KRT76 PE=1 SV=2
Q02083	3	3	17.8603	1.39E-06	29.51218	P200	P18	N-acylethanolamine-hydrolyzing acid amidase OS=Homo sapiens GN=NAAA PE=1 SV=3
Q02153	4	3	19.8755	0.048081	1.381065	P200	P18	Guanylate cyclase soluble subunit beta-1 OS=Homo sapiens GN=GUCY1B3 PE=1 SV=1
Q02413	4	3	25.5868	9.63E-05	1.707056	P200	P18	Desmoglein-1 OS=Homo sapiens GN=DSG1 PE=1 SV=2
Q02487	8	7	51.6284	8.99E-05	3.278154	P200	P18	Desmocollin-2 OS=Homo sapiens GN=DSC2 PE=1 SV=1
Q02790	2	2	9.4303	0.004635	1.320367	P200	P18	Peptidyl-prolyl cis-trans isomerase FKBP4 OS=Homo sapiens GN=FKBP4 PE=1 SV=3

Q04695	20	5	130.4566	0.212464	1.310525	P200	P18	Keratin, type I cytoskeletal 17 OS=Homo sapiens GN=KRT17 PE=1 SV=2
Q06830	13	12	82.4732	0.937517	1.010748	P200	P18	Peroxiredoxin-1 OS=Homo sapiens GN=PRDX1 PE=1 SV=1
Q07075	33	32	252.3323	2.55E-06	5.658045	P200	P18	Glutamyl aminopeptidase OS=Homo sapiens GN=ENPEP PE=1 SV=3
Q07507	4	4	32.8296	3.65E-07	37.34121	P200	P18	Dermatopontin OS=Homo sapiens GN=DPT PE=1 SV=2
Q07837	16	16	114.7155	0.003221	1.47208	P200	P18	Neutral and basic amino acid transport protein rBAT OS=Homo sapiens GN=SLC3A1 PE=1 SV=2
Q08380	79	77	390.0018	8.16E-08	272.2368	P200	P18	Galectin-3-binding protein OS=Homo sapiens GN=LGALS3BP PE=1 SV=1
Q09328	3	3	16.6255	0.001822	1.879182	P200	P18	Alpha-1,6-mannosylglycoprotein 6-beta-N-acetylglucosaminyltransferase A OS=Homo sapiens GN=MGAT5 PE=2 SV=1
Q12794	5	5	38.6856	2.10E-05	12.80856	P200	P18	Hyaluronidase-1 OS=Homo sapiens GN=HYAL1 PE=1 SV=2
Q12805	13	11	105.0878	1.02E-06	5.718591	P200	P18	EGF-containing fibulin-like extracellular matrix protein 1 OS=Homo sapiens GN=EFEMP1 PE=1 SV=2
Q12860	5	5	31.5342	2.05E-05	8.269527	P200	P18	Contactin-1 OS=Homo sapiens GN=CNTN1 PE=1 SV=1
Q12907	42	41	210.3653	1.65E-06	6.827963	P200	P18	Vesicular integral-membrane protein VIP36 OS=Homo sapiens GN=LMAN2 PE=1 SV=1
Q12929	4	4	20.7306	0.00302	2.358795	P200	P18	Epidermal growth factor receptor kinase substrate 8 OS=Homo sapiens GN=EPS8 PE=1 SV=1
Q12931	6	3	34.9186	0.001287	3.481269	P200	P18	Heat shock protein 75 kDa, mitochondrial OS=Homo sapiens GN=TRAP1 PE=1 SV=3
Q13242	3	2	12.9926	1.24E-06	103.211	P200	P18	Serine/arginine-rich splicing factor 9 OS=Homo sapiens GN=SRSF9 PE=1 SV=1
Q13277	2	2	10.0881	0.274202	1.362162	P200	P18	Syntaxin-3 OS=Homo sapiens GN=STX3 PE=1 SV=3
Q13287	2	2	9.9865	0.015781	147.8716	P200	P18	N-myc-interactor OS=Homo sapiens GN=NMI PE=1 SV=2
Q13510	39	38	395.0782	4.70E-08	25.7757	P200	P18	Acid ceramidase OS=Homo sapiens GN=ASAH1 PE=1 SV=5
Q14108	3	2	18.2671	1.36E-09	Infinity	P200	P18	Lysosome membrane protein 2 OS=Homo sapiens GN=SCARB2 PE=1 SV=2

Q14254	3	3	22.5312	3.06E-05	23.30027	P200	P18	Flotillin-2 OS=Homo sapiens GN=FLOT2 PE=1 SV=2
Q14314	17	13	163.1438	1.92E-07	19.1521	P200	P18	Fibroleukin OS=Homo sapiens GN=FGL2 PE=1 SV=1
Q14344	8	3	34.2178	4.24E-05	3.958071	P200	P18	Guanine nucleotide-binding protein subunit alpha-13 OS=Homo sapiens GN=GNA13 PE=1 SV=2
Q14624	10	8	63.422	8.91E-07	7.88477	P200	P18	Inter-alpha-trypsin inhibitor heavy chain H4 OS=Homo sapiens GN=ITIH4 PE=1 SV=4
Q14697	2	2	10.947	1.61E-05	62.29734	P200	P18	Neutral alpha-glucosidase AB OS=Homo sapiens GN=GANAB PE=1 SV=3
Q14894	6	5	34.0382	0.000618	1.86839	P200	P18	Ketimine reductase mu-crystallin OS=Homo sapiens GN=CRYM PE=1 SV=1
Q14914	11	8	77.1152	0.000121	2.663788	P200	P18	Prostaglandin reductase 1 OS=Homo sapiens GN=PTGR1 PE=1 SV=2
Q15286	7	3	36.8711	0.000402	3.812043	P200	P18	Ras-related protein Rab-35 OS=Homo sapiens GN=RAB35 PE=1 SV=1
Q15828	3	3	26.386	1.18E-07	12.68189	P200	P18	Cystatin-M OS=Homo sapiens GN=CST6 PE=1 SV=1
Q16270	3	3	23.9741	9.08E-05	7.194003	P200	P18	Insulin-like growth factor-binding protein 7 OS=Homo sapiens GN=IGFBP7 PE=1 SV=1
Q16706	13	12	67.6868	3.29E-06	8.773957	P200	P18	Alpha-mannosidase 2 OS=Homo sapiens GN=MAN2A1 PE=1 SV=2
Q16769	4	4	31.3837	2.67E-05	17.32382	P200	P18	Glutaminyl-peptide cyclotransferase OS=Homo sapiens GN=QPCT PE=1 SV=1
Q29960;P04222;P30484;P30504;P30505;P30508;P30510;Q07000;Q29865;Q29963;Q9TNN7	7	3	41.157	1.53E-08	17.06602	P200	P18	HLA class I histocompatibility antigen, Cw-16 alpha chain OS=Homo sapiens GN=HLA-C PE=1 SV=1
Q92820	11	10	110.8089	8.03E-08	9.70017	P200	P18	Gamma-glutamyl hydrolase OS=Homo sapiens GN=GGH PE=1 SV=2
Q92956	4	3	20.1929	0.034158	2.307134	P200	P18	Tumor necrosis factor receptor superfamily member 14 OS=Homo sapiens GN=TNFRSF14 PE=1 SV=3
Q93099	4	3	23.144	0.00011	3.435995	P200	P18	Homogentisate 1,2-dioxygenase OS=Homo sapiens GN=HGD PE=1 SV=2
Q99497	2	2	18.307	0.708695	1.139272	P200	P18	Protein deglycase DJ-1 OS=Homo sapiens GN=PARK7 PE=1 SV=2

Q99519	4	4	33.5352	3.88E-05	5.595892	P200	P18	Sialidase-1 OS=Homo sapiens GN=NEU1 PE=1 SV=1
Q99536	6	6	39.5879	0.597074	1.141933	P200	P18	Synaptic vesicle membrane protein VAT-1 homolog OS=Homo sapiens GN=VAT1 PE=1 SV=2
Q99816	10	9	70.4112	1.03E-05	4.257919	P200	P18	Tumor susceptibility gene 101 protein OS=Homo sapiens GN=TSG101 PE=1 SV=2

Supplementary Table 2: Protein significantly different in the S-Plot between P18 and P200 are given here with proteins names, highest mean conditions and p[1] loading and p(corr)[1] values. Proteins passing the cutoff of +0.9 or -0.9 only are included here. The most statistically differentially abundant proteins between the P18 and P200 are highlighted in yellow.

Highest Mean	Lowest Mean	Protein name	p[1]	p(corr)[1]
P200	P18	Gamma-interferon-inducible lysosomal thiol reductase OS=Homo sapiens GN=IFI30 PE=1 SV=3	0.00463022	0.987664
P200	P18	Transmembrane 7 superfamily member 3 OS=Homo sapiens GN=TM7SF3 PE=2 SV=1	0.00627019	0.992792
P200	P18	Lysosome membrane protein 2 OS=Homo sapiens GN=SCARB2 PE=1 SV=2	0.00486384	0.998324
P200	P18	HLA class I histocompatibility antigen, A-26 alpha chain OS=Homo sapiens GN=HLA-A PE=1 SV=2	0.00405396	0.999094
P200	P18	Sucrase-isomaltase, intestinal OS=Homo sapiens GN=SI PE=1 SV=6	0.0114027	0.994532
P200	P18	Angiotensin-converting enzyme OS=Homo sapiens GN=ACE PE=1 SV=1	0.0148135	0.905276
P200	P18	Galectin-3-binding protein OS=Homo sapiens GN=LGALS3BP PE=1 SV=1	0.23507	0.995984
P200	P18	Kinesin heavy chain isoform 5C OS=Homo sapiens GN=KIF5C PE=1 SV=1	0.014091	0.953419
P200	P18	Insulin-like growth factor-binding protein complex acid labile subunit OS=Homo sapiens GN=IGFALS PE=1 SV=1	0.00712225	0.967699
P200	P18	Keratin, type I cuticular Ha7 OS=Homo sapiens GN=KRT37 PE=3 SV=3	0.00785633	0.994394
P200	P18	F-box/LRR-repeat protein 14 OS=Homo sapiens GN=FBXL14 PE=1 SV=1	0.0169181	0.999493
P200	P18	Serine/arginine-rich splicing factor 9 OS=Homo sapiens GN=SRSF9 PE=1 SV=1	0.0219406	0.998378
P200	P18	Calcium-binding protein 8 OS=Homo sapiens GN=CALN1 PE=2 SV=1	0.0106104	0.988266
P200	P18	Alpha-N-acetylgalactosaminidase OS=Homo sapiens GN=NAGA PE=1 SV=2	0.00991876	0.992781
P200	P18	Leucine-rich alpha-2-glycoprotein OS=Homo sapiens GN=LRG1 PE=1 SV=2	0.0199737	0.998167
P200	P18	Biotinidase OS=Homo sapiens GN=BTD PE=1 SV=2	0.0200877	0.999794
P200	P18	Neurexin-3 OS=Homo sapiens GN=NRXN3 PE=1 SV=4	0.00633508	0.990035
P200	P18	Ig kappa chain V-IV region JI OS=Homo sapiens PE=4 SV=1	0.0145247	0.999535
P200	P18	Ig alpha-1 chain C region OS=Homo sapiens GN=IGHA1 PE=1 SV=2	0.15557	0.999523
P200	P18	Ig mu chain C region OS=Homo sapiens GN=IGHM PE=1 SV=3	0.0454902	0.97643
P200	P18	Polymeric immunoglobulin receptor OS=Homo sapiens GN=PIGR PE=1 SV=4	0.166479	0.996223
P200	P18	Ig alpha-2 chain C region OS=Homo sapiens GN=IGHA2 PE=1 SV=3	0.0790342	0.983339
P200	P18	Neutral alpha-glucosidase AB OS=Homo sapiens GN=GANAB PE=1 SV=3	0.00648712	0.945248
P200	P18	Transmembrane protease serine 2 OS=Homo sapiens GN=TMPRSS2 PE=1 SV=3	0.0363425	0.939542
P200	P18	Spermine synthase OS=Homo sapiens GN=SMS PE=1 SV=2	0.0174377	0.993833

P200	P18	Alpha-N-acetylglucosaminidase OS=Homo sapiens GN=NAGLU PE=1 SV=2	0.116844	0.998782
P200	P18	Carboxypeptidase Q OS=Homo sapiens GN=CPQ PE=1 SV=1	0.0176518	0.999243
P200	P18	Glypican-3 OS=Homo sapiens GN=GPC3 PE=1 SV=1	0.0130615	0.988221
P200	P18	Lysosomal protective protein OS=Homo sapiens GN=CTSA PE=1 SV=2	0.129906	0.998213
P200	P18	Thyroxine-binding globulin OS=Homo sapiens GN=SERPINA7 PE=1 SV=2	0.0321345	0.992482
P200	P18	Immunoglobulin lambda-like polypeptide 5 OS=Homo sapiens GN=IGLL5 PE=2 SV=2	0.0279484	0.999516
P200	P18	Transcriptional activator protein Pur-beta OS=Homo sapiens GN=PURB PE=1 SV=3	0.0173622	0.986604
P200	P18	Bile salt-activated lipase OS=Homo sapiens GN=CEL PE=1 SV=3	0.017159	0.999349
P200	P18	Anthrax toxin receptor 1 OS=Homo sapiens GN=ANTXR1 PE=1 SV=2	0.0168714	0.998857
P200	P18	CD81 antigen OS=Homo sapiens GN=CD81 PE=1 SV=1	0.0302339	0.990716
P200	P18	Carboxypeptidase N subunit 2 OS=Homo sapiens GN=CPN2 PE=1 SV=3	0.0498697	0.997447
P200	P18	Dermatopontin OS=Homo sapiens GN=DPT PE=1 SV=2	0.0341821	0.999559
P200	P18	Retinoid-inducible serine carboxypeptidase OS=Homo sapiens GN=SCPEP1 PE=1 SV=1	0.0274326	0.996327
P200	P18	Ammonium transporter Rh type C OS=Homo sapiens GN=RHCG PE=1 SV=1	0.0241822	0.999616
P200	P18	Kallistatin OS=Homo sapiens GN=SERPINA4 PE=1 SV=3	0.0140028	0.998179
P200	P18	Plasma alpha-L-fucosidase OS=Homo sapiens GN=FUCA2 PE=1 SV=2	0.0260044	0.997697
P200	P18	Immunoglobulin J chain OS=Homo sapiens GN=JCHAIN PE=1 SV=4	0.0847084	0.999494
P200	P18	Ig heavy chain V-III region BRO OS=Homo sapiens PE=1 SV=1	0.0107721	0.989701
P200	P18	Ceruloplasmin OS=Homo sapiens GN=CP PE=1 SV=1	0.120064	0.999702
P200	P18	Beta-galactosidase OS=Homo sapiens GN=GLB1 PE=1 SV=2	0.216799	0.999177
P200	P18	N-acylethanolamine-hydrolyzing acid amidase OS=Homo sapiens GN=NAAA PE=1 SV=3	0.0136192	0.997855
P200	P18	Extracellular superoxide dismutase [Cu-Zn] OS=Homo sapiens GN=SOD3 PE=1 SV=2	0.0251235	0.996979
P200	P18	CD9 antigen OS=Homo sapiens GN=CD9 PE=1 SV=4	0.0521228	0.992166
P200	P18	Cadherin-11 OS=Homo sapiens GN=CDH11 PE=2 SV=2	0.0147288	0.912548
P200	P18	Beta-1,4-galactosyltransferase 1 OS=Homo sapiens GN=B4GALT1 PE=1 SV=5	0.0125502	0.973958
P200	P18	Acid ceramidase OS=Homo sapiens GN=ASAH1 PE=1 SV=5	0.130248	0.998712
P200	P18	Cadherin-1 OS=Homo sapiens GN=CDH1 PE=1 SV=3	0.0164633	0.983128
P18	P200	Coactosin-like protein OS=Homo sapiens GN=COTL1 PE=1 SV=3	-0.00816636	-0.989413
P200	P18	Ribonuclease T2 OS=Homo sapiens GN=RNASET2 PE=1 SV=2	0.0207142	0.924294
P200	P18	Mucin-13 OS=Homo sapiens GN=MUC13 PE=1 SV=3	0.00936278	0.981835
P200	P18	Polypeptide N-acetylgalactosaminyltransferase 18 OS=Homo sapiens GN=GALNT18 PE=2 SV=2	0.00904841	0.987073
P200	P18	Ig mu heavy chain disease protein OS=Homo sapiens PE=1 SV=1	0.0137679	0.989799
P200	P18	Phosphatidylcholine transfer protein OS=Homo sapiens GN=PCTP PE=1 SV=1	0.0199178	0.99844
P200	P18	Lysosomal alpha-glucosidase OS=Homo sapiens GN=GAA PE=1 SV=4	0.0587398	0.999711
P200	P18	Vitamin K-dependent protein Z OS=Homo sapiens GN=PROZ PE=1 SV=2	0.0675589	0.997014
P200	P18	Flotillin-2 OS=Homo sapiens GN=FLOT2 PE=1 SV=2	0.00793889	0.982785
P200	P18	Alpha-galactosidase A OS=Homo sapiens GN=GLA PE=1 SV=1	0.0427062	0.999377
P200	P18	Cadherin-related family member 2 OS=Homo sapiens GN=CDHR2 PE=1 SV=2	0.026677	0.999989
P200	P18	Complement component C9 OS=Homo sapiens GN=C9 PE=1 SV=2	0.0385225	0.999167
P200	P18	Nuclear transport factor 2 OS=Homo sapiens GN=NUTF2 PE=1 SV=1	0.033363	0.983707
P200	P18	Tubulin beta-4B chain OS=Homo sapiens GN=TUBB4B PE=1 SV=1	0.0404006	0.996529

P200	P18	Vacuolar protein sorting-associated protein 37B OS=Homo sapiens GN=VPS37B PE=1 SV=1	0.00652707	0.966549
P200	P18	Tissue alpha-L-fucosidase OS=Homo sapiens GN=FUCA1 PE=1 SV=4	0.0244618	0.999915
P200	P18	Ig gamma-4 chain C region OS=Homo sapiens GN=IGHG4 PE=1 SV=1	0.0257662	0.999421
P200	P18	Microfibril-associated glycoprotein 4 OS=Homo sapiens GN=MFAP4 PE=1 SV=2	0.0169372	0.999026
P200	P18	Calpain-5 OS=Homo sapiens GN=CAPN5 PE=1 SV=2	0.0138268	0.997156
P200	P18	Ig lambda chain V-III region SH OS=Homo sapiens PE=1 SV=1	0.0139814	0.989668
P200	P18	Glycerol-3-phosphate dehydrogenase 1-like protein OS=Homo sapiens GN=GPD1L PE=1 SV=1	0.00881221	0.992839
P200	P18	Guanine nucleotide-binding protein subunit alpha-11 OS=Homo sapiens GN=GNA11 PE=1 SV=2	0.038766	0.999771
P200	P18	Fibroleukin OS=Homo sapiens GN=FGL2 PE=1 SV=1	0.0529234	0.99813
P200	P18	Beta-glucuronidase OS=Homo sapiens GN=GUSB PE=1 SV=2	0.0185702	0.999539
P200	P18	Dipeptidyl peptidase 1 OS=Homo sapiens GN=CTSC PE=1 SV=2	0.0903499	0.999328
P200	P18	Dipeptidyl peptidase 2 OS=Homo sapiens GN=DPP7 PE=1 SV=3	0.00560649	0.935001
P200	P18	Ephrin type-B receptor 4 OS=Homo sapiens GN=EPHB4 PE=1 SV=2	0.0189999	0.926631
P200	P18	Putative heat shock protein HSP 90-beta 2 OS=Homo sapiens GN=HSP90AB2P PE=1 SV=2	0.00849782	0.996265
P200	P18	Clusterin OS=Homo sapiens GN=CLU PE=1 SV=1	0.12778	0.999378
P200	P18	Prominin-1 OS=Homo sapiens GN=PROM1 PE=1 SV=1	0.0793262	0.995576
P200	P18	Glutaminyl-peptide cyclotransferase OS=Homo sapiens GN=QPCT PE=1 SV=1	0.0138592	0.981111
P200	P18	Thyrotropin-releasing hormone-degrading ectoenzyme OS=Homo sapiens GN=TRHDE PE=2 SV=1	0.0206349	0.995285
P200	P18	HLA class I histocompatibility antigen, Cw-16 alpha chain OS=Homo sapiens GN=HLA-C PE=1 SV=1	0.0791277	0.999973
P200	P18	Monocyte differentiation antigen CD14 OS=Homo sapiens GN=CD14 PE=1 SV=2	0.020734	0.99936
P200	P18	Arylsulfatase A OS=Homo sapiens GN=ARSA PE=1 SV=3	0.0464056	0.98446
P200	P18	N-acetylglucosamine-6-sulfatase OS=Homo sapiens GN=GNS PE=1 SV=3	0.0210843	0.966229
P200	P18	Arylsulfatase B OS=Homo sapiens GN=ARSB PE=1 SV=1	0.0109018	0.988204
P200	P18	Pro-epidermal growth factor OS=Homo sapiens GN=EGF PE=1 SV=2	0.237363	0.999811
P200	P18	Serpin B3 OS=Homo sapiens GN=SERPINB3 PE=1 SV=2	0.0057386	0.97663
P200	P18	C-type lectin domain family 14 member A OS=Homo sapiens GN=CLEC14A PE=1 SV=1	0.0118446	0.99602
P200	P18	Eukaryotic translation initiation factor 6 OS=Homo sapiens GN=EIF6 PE=1 SV=1	0.0144761	0.99901
P200	P18	G-protein coupled receptor family C group 5 member B OS=Homo sapiens GN=GPRC5B PE=2 SV=2	0.0411441	0.997656
P200	P18	Thrombospondin-1 OS=Homo sapiens GN=THBS1 PE=1 SV=2	0.0117709	0.956241
P200	P18	Tripeptidyl-peptidase 1 OS=Homo sapiens GN=TPP1 PE=1 SV=2	0.107447	0.999938
P200	P18	Ras-related protein Rab-3A OS=Homo sapiens GN=RAB3A PE=1 SV=1	0.0159511	0.984415
P200	P18	Biglycan OS=Homo sapiens GN=BGN PE=1 SV=2	0.0089511	0.993937
P200	P18	N(G),N(G)-dimethylarginine dimethylaminohydrolase 1 OS=Homo sapiens GN=DDAH1 PE=1 SV=3	0.0282027	0.991949
P200	P18	Complement C1r subcomponent-like protein OS=Homo sapiens GN=C1RL PE=1 SV=2	0.0121753	0.996718
P200	P18	Calmodulin-like protein 5 OS=Homo sapiens GN=CALML5 PE=1 SV=2	0.0160014	0.998478
P200	P18	Ig heavy chain V-III region BUT OS=Homo sapiens PE=1 SV=1	0.00631015	0.997776
P200	P18	Fibrinogen alpha chain OS=Homo sapiens GN=FGA PE=1 SV=2	0.149388	0.999558
P200	P18	Plasma protease C1 inhibitor OS=Homo sapiens GN=SERPING1 PE=1 SV=2	0.0547234	0.995649

P200	P18	Argininosuccinate lyase OS=Homo sapiens GN=ASL PE=1 SV=4	0.0351409	0.999898
P200	P18	Endothelial protein C receptor OS=Homo sapiens GN=PROCR PE=1 SV=1	0.013296	0.948327
P200	P18	Syntenin-1 OS=Homo sapiens GN=SDCBP PE=1 SV=1	0.106527	0.999392
P200	P18	Tetraspanin-1 OS=Homo sapiens GN=TSPAN1 PE=1 SV=2	0.0553186	0.999838
P200	P18	Probable serine carboxypeptidase CPVL OS=Homo sapiens GN=CPVL PE=1 SV=2	0.0678791	0.995037
P200	P18	Ferritin light chain OS=Homo sapiens GN=FTL PE=1 SV=2	0.0299833	0.996689
P200	P18	Copine-3 OS=Homo sapiens GN=CPNE3 PE=1 SV=1	0.0214601	0.99973
P200	P18	Ig heavy chain V-III region 23 OS=Homo sapiens GN=IGHV3-23 PE=1 SV=2	0.0152385	0.978894
P200	P18	Hyaluronidase-1 OS=Homo sapiens GN=HYAL1 PE=1 SV=2	0.0229749	0.976234
P200	P18	Cystatin-M OS=Homo sapiens GN=CST6 PE=1 SV=1	0.015236	0.999666
P200	P18	Cathepsin B OS=Homo sapiens GN=CTSB PE=1 SV=3	0.0461727	0.990182
P200	P18	Intelectin-1 OS=Homo sapiens GN=ITLN1 PE=1 SV=1	0.0143368	0.996368
P200	P18	Pepsin A-3 OS=Homo sapiens GN=PGA3 PE=1 SV=1	0.0258116	0.997763
P200	P18	Serum albumin OS=Homo sapiens GN=ALB PE=1 SV=2	0.278344	0.999892
P200	P18	Cholesteryl ester transfer protein OS=Homo sapiens GN=CETP PE=1 SV=2	0.0164402	0.990449
P200	P18	Transcriptional adapter 2-alpha OS=Homo sapiens GN=TADA2A PE=1 SV=3	0.0409294	0.999003
P200	P18	Charged multivesicular body protein 1b OS=Homo sapiens GN=CHMP1B PE=1 SV=1	0.0173022	0.962517
P200	P18	Ig gamma-2 chain C region OS=Homo sapiens GN=IGHG2 PE=1 SV=2	0.0802132	0.99288
P200	P18	HIV Tat-specific factor 1 OS=Homo sapiens GN=HTATSF1 PE=1 SV=1	0.00483879	0.988365
P18	P200	Complement factor D OS=Homo sapiens GN=CFD PE=1 SV=5	-0.0188449	-0.994905
P200	P18	Peptidoglycan recognition protein 1 OS=Homo sapiens GN=PGLYRP1 PE=1 SV=1	0.0305695	0.988349
P200	P18	G-protein coupled receptor family C group 5 member C OS=Homo sapiens GN=GPRC5C PE=1 SV=2	0.0416075	0.993124
P200	P18	Zymogen granule protein 16 homolog B OS=Homo sapiens GN=ZG16B PE=1 SV=3	0.0255771	0.996416
P200	P18	Maltase-glucoamylase, intestinal OS=Homo sapiens GN=MGAM PE=1 SV=5	0.122294	0.997186
P200	P18	Sushi domain-containing protein 2 OS=Homo sapiens GN=SUSD2 PE=1 SV=1	0.0707976	0.99925
P200	P18	Alpha-1-antitrypsin OS=Homo sapiens GN=SERPINA1 PE=1 SV=3	0.0875429	0.997338
P200	P18	Sorting nexin-6 OS=Homo sapiens GN=SNX6 PE=1 SV=1	0.0119907	0.912288
P200	P18	Thioredoxin-like protein 1 OS=Homo sapiens GN=TXNL1 PE=1 SV=3	0.0335242	0.999591
P200	P18	Alpha-1-antichymotrypsin OS=Homo sapiens GN=SERPINA3 PE=1 SV=2	0.0336989	0.999784
P200	P18	MIT domain-containing protein 1 OS=Homo sapiens GN=MITD1 PE=1 SV=1	0.0112286	0.988345
P200	P18	CD44 antigen OS=Homo sapiens GN=CD44 PE=1 SV=3	0.0227819	0.994567
P200	P18	Ig gamma-1 chain C region OS=Homo sapiens GN=IGHG1 PE=1 SV=1	0.078546	0.977378
P200	P18	Choline transporter-like protein 2 OS=Homo sapiens GN=SLC44A2 PE=1 SV=3	0.0218066	0.999048
P200	P18	Beta-hexosaminidase subunit beta OS=Homo sapiens GN=HEXB PE=1 SV=3	0.0133654	0.976822
P200	P18	Alpha-1B-glycoprotein OS=Homo sapiens GN=A1BG PE=1 SV=4	0.034767	0.991488
P200	P18	Collagen alpha-1(XV) chain OS=Homo sapiens GN=COL15A1 PE=1 SV=2	0.049304	0.998972
P200	P18	ADP-ribosylation factor-like protein 15 OS=Homo sapiens GN=ARL15 PE=1 SV=1	0.00767292	0.995193
P200	P18	Lysosomal acid phosphatase OS=Homo sapiens GN=ACP2 PE=1 SV=3	0.0323368	0.99244
P200	P18	Serotransferrin OS=Homo sapiens GN=TF PE=1 SV=3	0.0591289	0.999536
P200	P18	Pro-cathepsin H OS=Homo sapiens GN=CTSH PE=1 SV=4	0.0148093	0.988637

P200	P18	Amiloride-sensitive amine oxidase [copper-containing] OS=Homo sapiens GN=AOC1 PE=1 SV=4	0.028746	0.997654
P200	P18	CD177 antigen OS=Homo sapiens GN=CD177 PE=1 SV=2	0.0087004	0.994099
P200	P18	Lysosomal Pro-X carboxypeptidase OS=Homo sapiens GN=PRCP PE=1 SV=1	0.0338915	0.997622
P200	P18	Gamma-glutamyl hydrolase OS=Homo sapiens GN=GGH PE=1 SV=2	0.0453294	0.999776
P200	P18	BRO1 domain-containing protein BROX OS=Homo sapiens GN=BROX PE=1 SV=1	0.0393393	0.99715
P200	P18	Cathepsin D OS=Homo sapiens GN=CTSD PE=1 SV=1	0.0679602	0.995833
P200	P18	Vacuolar protein sorting-associated protein 28 homolog OS=Homo sapiens GN=VPS28 PE=1 SV=1	0.0191238	0.996666
P200	P18	N-acetylgalactosamine-6-sulfatase OS=Homo sapiens GN=GALNS PE=1 SV=1	0.0305205	0.985056
P200	P18	Sodium/potassium-transporting ATPase subunit alpha-3 OS=Homo sapiens GN=ATP1A3 PE=1 SV=3	0.0180578	0.992928
P200	P18	Resistin OS=Homo sapiens GN=RETN PE=1 SV=1	0.0198583	0.990447
P200	P18	45 kDa calcium-binding protein OS=Homo sapiens GN=SDF4 PE=1 SV=1	0.00848073	0.992314
P200	P18	Carboxypeptidase M OS=Homo sapiens GN=CPM PE=1 SV=2	0.0188488	0.963071
P200	P18	Angiopoietin-related protein 2 OS=Homo sapiens GN=ANGPTL2 PE=2 SV=1	0.0797494	0.992091
P200	P18	Flotillin-1 OS=Homo sapiens GN=FLOT1 PE=1 SV=3	0.0157706	0.987389
P200	P18	Alpha-mannosidase 2 OS=Homo sapiens GN=MAN2A1 PE=1 SV=2	0.019326	0.993928
P200	P18	Beta-hexosaminidase subunit alpha OS=Homo sapiens GN=HEXA PE=1 SV=2	0.0149981	0.991785
P200	P18	Mannosyl-oligosaccharide 1,2-alpha-mannosidase IA OS=Homo sapiens GN=MAN1A1 PE=1 SV=3	0.068419	0.996817
P200	P18	Nidogen-1 OS=Homo sapiens GN=NID1 PE=1 SV=3	0.0556789	0.994264
P200	P18	Alpha-2-macroglobulin OS=Homo sapiens GN=A2M PE=1 SV=3	0.0361791	0.99741
P200	P18	Matrix-remodeling-associated protein 8 OS=Homo sapiens GN=MXRA8 PE=1 SV=1	0.0593286	0.997664
P200	P18	Ig lambda chain V-I region WAH OS=Homo sapiens PE=1 SV=1	0.0099162	0.999014
P200	P18	Contactin-1 OS=Homo sapiens GN=CNTN1 PE=1 SV=1	0.0102548	0.992306
P200	P18	Epididymis-specific alpha-mannosidase OS=Homo sapiens GN=MAN2B2 PE=1 SV=4	0.0205007	0.964439
P200	P18	IST1 homolog OS=Homo sapiens GN=IST1 PE=1 SV=1	0.0567308	0.999232
P200	P18	Cytosol aminopeptidase OS=Homo sapiens GN=LAP3 PE=1 SV=3	0.0638804	0.979075
P200	P18	Copine-8 OS=Homo sapiens GN=CPNE8 PE=1 SV=2	0.0194343	0.975191
P200	P18	Prosaposin OS=Homo sapiens GN=PSAP PE=1 SV=2	0.089521	0.999013
P200	P18	Inter-alpha-trypsin inhibitor heavy chain H4 OS=Homo sapiens GN=ITIH4 PE=1 SV=4	0.0324376	0.998423
P200	P18	Haptoglobin OS=Homo sapiens GN=HP PE=1 SV=1	0.0858123	0.999221
P200	P18	Alpha-1-acid glycoprotein 1 OS=Homo sapiens GN=ORM1 PE=1 SV=1	0.0202734	0.995613
P200	P18	Antithrombin-III OS=Homo sapiens GN=SERPINC1 PE=1 SV=1	0.0347463	0.994299
P200	P18	Mitochondrial import inner membrane translocase subunit Tim13 OS=Homo sapiens GN=TIMM13 PE=1 SV=1	0.0235304	0.999613
P18	P200	Guanine nucleotide-binding protein G(i) subunit alpha-1 OS=Homo sapiens GN=GNAI1 PE=1 SV=2	-0.0486322	-0.988921
P200	P18	Attractin OS=Homo sapiens GN=ATRN PE=1 SV=2	0.0606933	0.988719
P200	P18	Urokinase-type plasminogen activator OS=Homo sapiens GN=PLAU PE=1 SV=2	0.0113957	0.997174
P200	P18	Insulin-like growth factor-binding protein 7 OS=Homo sapiens GN=IGFBP7 PE=1 SV=1	0.0143762	0.997392
P200	P18	Keratin, type II cytoskeletal 5 OS=Homo sapiens GN=KRT5 PE=1 SV=3	0.0610445	0.999308
P200	P18	Olfactomedin-4 OS=Homo sapiens GN=OLFM4 PE=1 SV=1	0.105346	0.99951

P200	P18	Multivesicular body subunit 12A OS=Homo sapiens GN=MVB12A PE=1 SV=1	0.0100025	0.99466
P200	P18	Vitamin D-binding protein OS=Homo sapiens GN=GC PE=1 SV=1	0.0222084	0.943531
P200	P18	Ras-related protein Rap-1A OS=Homo sapiens GN=RAP1A PE=1 SV=1	0.0250879	0.997671
P200	P18	Ig kappa chain V-III region WOL OS=Homo sapiens PE=1 SV=1	0.0382987	0.999755
P200	P18	Vesicular integral-membrane protein VIP36 OS=Homo sapiens GN=LMAN2 PE=1 SV=1	0.110974	0.996309
P200	P18	Target of Myb protein 1 OS=Homo sapiens GN=TOM1 PE=1 SV=2	0.00667487	0.99221
P200	P18	PDZ domain-containing protein GIPC2 OS=Homo sapiens GN=GIPC2 PE=1 SV=1	0.00599544	0.948514
P200	P18	U3 small nucleolar RNA-associated protein 14 homolog A OS=Homo sapiens GN=UTP14A PE=1 SV=1	0.0386127	0.984799
P200	P18	Ig kappa chain C region OS=Homo sapiens GN=IGKC PE=1 SV=1	0.208688	0.999777
P200	P18	Adenosylhomocysteinase OS=Homo sapiens GN=AHCY PE=1 SV=4	0.00991743	0.974298
P200	P18	Kininogen-1 OS=Homo sapiens GN=KNG1 PE=1 SV=2	0.140607	0.999366
P200	P18	Complement C3 OS=Homo sapiens GN=C3 PE=1 SV=2	0.0658114	0.999103
P200	P18	WD repeat-containing protein 1 OS=Homo sapiens GN=WDR1 PE=1 SV=4	0.0307138	0.991757
P200	P18	Vacuolar protein sorting-associated protein 4B OS=Homo sapiens GN=VPS4B PE=1 SV=2	0.0280072	0.997218
P200	P18	Non-secretory ribonuclease OS=Homo sapiens GN=RNASE2 PE=1 SV=2	0.0215716	0.998051
P200	P18	Heat shock 70 kDa protein 6 OS=Homo sapiens GN=HSPA6 PE=1 SV=2	0.0237189	0.999118
P200	P18	Plasma serine protease inhibitor OS=Homo sapiens GN=SERPINA5 PE=1 SV=3	0.0649208	0.998341
P200	P18	N-acetylmuramoyl-L-alanine amidase OS=Homo sapiens GN=PGLYRP2 PE=1 SV=1	0.0234157	0.994849
P200	P18	3-mercaptopyruvate sulfurtransferase OS=Homo sapiens GN=MPST PE=1 SV=3	0.0175406	0.912039
P200	P18	Cathepsin L1 OS=Homo sapiens GN=CTSL PE=1 SV=2	0.017238	0.993629
P200	P18	Thy-1 membrane glycoprotein OS=Homo sapiens GN=THY1 PE=1 SV=2	0.0197941	0.998355
P200	P18	Hemopexin OS=Homo sapiens GN=HPX PE=1 SV=2	0.0546247	0.994428
P200	P18	Heat shock cognate 71 kDa protein OS=Homo sapiens GN=HSPA8 PE=1 SV=1	0.0539262	0.992427
P200	P18	Serum amyloid P-component OS=Homo sapiens GN=APCS PE=1 SV=2	0.0188658	0.984954
P200	P18	Prenylcysteine oxidase 1 OS=Homo sapiens GN=PCYOX1 PE=1 SV=3	0.00723606	0.927741
P200	P18	Renin receptor OS=Homo sapiens GN=ATP6AP2 PE=1 SV=2	0.0110112	0.995373
P200	P18	EGF-containing fibulin-like extracellular matrix protein 1 OS=Homo sapiens GN=EFEMP1 PE=1 SV=2	0.038586	0.999326
P200	P18	U4/U6 small nuclear ribonucleoprotein Prp4 OS=Homo sapiens GN=PRPF4 PE=1 SV=2	0.039211	0.99869
P200	P18	Glutamyl aminopeptidase OS=Homo sapiens GN=ENPEP PE=1 SV=3	0.0447745	0.995392
P18	P200	Enoyl-CoA hydratase, mitochondrial OS=Homo sapiens GN=ECHS1 PE=1 SV=4	-0.00703316	-0.994977
P200	P18	Sialidase-1 OS=Homo sapiens GN=NEU1 PE=1 SV=1	0.018436	0.992276
P200	P18	Keratin, type I cytoskeletal 19 OS=Homo sapiens GN=KRT19 PE=1 SV=4	0.00648822	0.993575
P200	P18	Vitronectin OS=Homo sapiens GN=VTN PE=1 SV=1	0.0495769	0.996431
P200	P18	Lactotransferrin OS=Homo sapiens GN=LTF PE=1 SV=6	0.0443565	0.999108
P200	P18	Ganglioside GM2 activator OS=Homo sapiens GN=GM2A PE=1 SV=4	0.0263312	0.994696
P200	P18	Pappalysin-2 OS=Homo sapiens GN=PAPPA2 PE=1 SV=4	0.0310891	0.99337
P200	P18	Keratin, type II cytoskeletal 6A OS=Homo sapiens GN=KRT6A PE=1 SV=3	0.0155077	0.98207
P200	P18	Lysosomal alpha-mannosidase OS=Homo sapiens GN=MAN2B1 PE=1 SV=3	0.0284003	0.999414

P200	P18	Glutaredoxin domain-containing cysteine-rich protein 2 OS=Homo sapiens GN=GRXCR2 PE=3 SV=1	0.0184585	0.997183
P200	P18	Vacuolar protein sorting-associated protein 4A OS=Homo sapiens GN=VPS4A PE=1 SV=1	0.033109	0.993466
P200	P18	Pigment epithelium-derived factor OS=Homo sapiens GN=SERPINF1 PE=1 SV=4	0.0155318	0.997341
P200	P18	Rab GDP dissociation inhibitor alpha OS=Homo sapiens GN=GDI1 PE=1 SV=2	0.0355931	0.995457
P200	P18	Unconventional myosin-Ic OS=Homo sapiens GN=MYO1C PE=1 SV=4	0.0206381	0.946821
P200	P18	Lysosome-associated membrane glycoprotein 1 OS=Homo sapiens GN=LAMP1 PE=1 SV=3	0.0146917	0.990215
P200	P18	Prostatic acid phosphatase OS=Homo sapiens GN=ACPP PE=1 SV=3	0.0543599	0.992325
P200	P18	Vasorin OS=Homo sapiens GN=VASN PE=1 SV=1	0.0197979	0.993451
P18	P200	14-3-3 protein gamma OS=Homo sapiens GN=YWHAG PE=1 SV=2	-0.0118064	-0.992673
P200	P18	Chloride intracellular channel protein 1 OS=Homo sapiens GN=CLIC1 PE=1 SV=4	0.0450717	0.997562
P200	P18	Syndecan-1 OS=Homo sapiens GN=SDC1 PE=1 SV=3	0.0119712	0.995045
P200	P18	Vitelline membrane outer layer protein 1 homolog OS=Homo sapiens GN=VMO1 PE=1 SV=1	0.0453038	0.995398
P200	P18	Lysosome-associated membrane glycoprotein 2 OS=Homo sapiens GN=LAMP2 PE=1 SV=2	0.0112998	0.978969
P200	P18	Serine hydroxymethyltransferase, cytosolic OS=Homo sapiens GN=SHMT1 PE=1 SV=1	0.0360506	0.996209
P200	P18	Alpha-1-acid glycoprotein 2 OS=Homo sapiens GN=ORM2 PE=1 SV=2	0.00486933	0.996221
P200	P18	Carbonic anhydrase 4 OS=Homo sapiens GN=CA4 PE=1 SV=2	0.0191136	0.985702
P200	P18	Programmed cell death protein 6 OS=Homo sapiens GN=PDCD6 PE=1 SV=1	0.0350067	0.991079
P200	P18	Sodium/potassium-transporting ATPase subunit alpha-2 OS=Homo sapiens GN=ATP1A2 PE=1 SV=1	0.0178756	0.9897
P200	P18	Ig kappa chain V-IV region Len OS=Homo sapiens PE=1 SV=2	0.0230638	0.905712
P200	P18	Charged multivesicular body protein 5 OS=Homo sapiens GN=CHMP5 PE=1 SV=1	0.051877	0.99983
P200	P18	Agrin OS=Homo sapiens GN=AGRN PE=1 SV=5	0.0256422	0.994507
P200	P18	Apolipoprotein E OS=Homo sapiens GN=APOE PE=1 SV=1	0.0185594	0.982455
P200	P18	Ig heavy chain V-III region GAL OS=Homo sapiens PE=1 SV=1	0.0107438	0.993983
P200	P18	Vacuolar protein sorting-associated protein VTA1 homolog OS=Homo sapiens GN=VTA1 PE=1 SV=1	0.0274448	0.997811
P200	P18	Napsin-A OS=Homo sapiens GN=NAPSA PE=1 SV=1	0.0950882	0.997039
P200	P18	Granulins OS=Homo sapiens GN=GRN PE=1 SV=2	0.0210644	0.991778
P200	P18	Charged multivesicular body protein 4b OS=Homo sapiens GN=CHMP4B PE=1 SV=1	0.0185703	0.925449
P200	P18	Collagen alpha-1(VI) chain OS=Homo sapiens GN=COL6A1 PE=1 SV=3	0.0220305	0.996386
P18	P200	Glutathione S-transferase Mu 3 OS=Homo sapiens GN=GSTM3 PE=1 SV=3	-0.031283	-0.980532
P200	P18	Proteasome subunit alpha type-5 OS=Homo sapiens GN=PSMA5 PE=1 SV=3	0.0112304	0.992075
P200	P18	Melanotransferrin OS=Homo sapiens GN=MFI2 PE=1 SV=2	0.0317766	0.999912
P200	P18	Ig kappa chain V-II region TEW OS=Homo sapiens PE=1 SV=1	0.0191802	0.994447
P200	P18	Tumor susceptibility gene 101 protein OS=Homo sapiens GN=TSG101 PE=1 SV=2	0.020729	0.998132
P200	P18	Prostate stem cell antigen OS=Homo sapiens GN=PSCA PE=1 SV=1	0.0449743	0.991989
P18	P200	Retinoic acid-induced protein 3 OS=Homo sapiens GN=GPRC5A PE=1 SV=2	-0.00791438	-0.975382
P200	P18	6-phosphogluconolactonase OS=Homo sapiens GN=PGLS PE=1 SV=2	0.0351798	0.993916
P18	P200	Actin, alpha skeletal muscle OS=Homo sapiens GN=ACTA1 PE=1 SV=1	-0.0228478	-0.997151

P200	P18	Charged multivesicular body protein 2a OS=Homo sapiens GN=CHMP2A PE=1 SV=1	0.0333963	0.996736
P200	P18	Guanine nucleotide-binding protein subunit alpha-13 OS=Homo sapiens GN=GNA13 PE=1 SV=2	0.00927673	0.989367
P200	P18	Proteasome subunit alpha type-6 OS=Homo sapiens GN=PSMA6 PE=1 SV=1	0.00764897	0.902164
P200	P18	Hemoglobin subunit delta OS=Homo sapiens GN=HBD PE=1 SV=2	0.0131587	0.990631
P200	P18	Ubiquitin-conjugating enzyme E2 variant 3 OS=Homo sapiens GN=UEVLD PE=1 SV=2	0.0106713	0.986595
P200	P18	Aquaporin-2 OS=Homo sapiens GN=AQP2 PE=1 SV=1	0.0273973	0.99047
P200	P18	Ras-related protein Rab-35 OS=Homo sapiens GN=RAB35 PE=1 SV=1	0.00938124	0.950376
P200	P18	Leucine-, glutamate- and lysine-rich protein 1 OS=Homo sapiens GN=LEKR1 PE=2 SV=2	0.00928944	0.932745
P200	P18	Glycerophosphodiester phosphodiesterase domain-containing protein 3 OS=Homo sapiens GN=GDPD3 PE=2 SV=3	0.00728142	0.992963
P200	P18	Mucin-1 OS=Homo sapiens GN=MUC1 PE=1 SV=3	0.0569622	0.994673
P200	P18	Myosin light chain 6B OS=Homo sapiens GN=MYL6B PE=1 SV=1	0.00501625	0.945386
P200	P18	Apolipoprotein D OS=Homo sapiens GN=APOD PE=1 SV=1	0.168426	0.997236
P200	P18	EH domain-containing protein 1 OS=Homo sapiens GN=EHD1 PE=1 SV=2	0.0182616	0.959366
P200	P18	Podocin OS=Homo sapiens GN=NPHS2 PE=1 SV=1	0.00906401	0.991155
P200	P18	Prothrombin OS=Homo sapiens GN=F2 PE=1 SV=2	0.0482174	0.997833
P200	P18	Heat shock protein 75 kDa, mitochondrial OS=Homo sapiens GN=TRAP1 PE=1 SV=3	0.0159971	0.929344
P200	P18	Homogentisate 1,2-dioxygenase OS=Homo sapiens GN=HGD PE=1 SV=2	0.00716008	0.97455
P200	P18	Annexin A11 OS=Homo sapiens GN=ANXA11 PE=1 SV=1	0.0324556	0.998008
P200	P18	Guanine nucleotide-binding protein G(I)/G(S)/G(T) subunit beta-1 OS=Homo sapiens GN=GNB1 PE=1 SV=3	0.0154151	0.970355
P200	P18	EGF-containing fibulin-like extracellular matrix protein 2 OS=Homo sapiens GN=EFEMP2 PE=1 SV=3	0.0114646	0.99617
P200	P18	Desmocollin-2 OS=Homo sapiens GN=DSC2 PE=1 SV=1	0.015245	0.982098
P200	P18	Erythrocyte band 7 integral membrane protein OS=Homo sapiens GN=STOM PE=1 SV=3	0.0210276	0.992324
P200	P18	Solute carrier family 2, facilitated glucose transporter member 5 OS=Homo sapiens GN=SLC2A5 PE=1 SV=1	0.0337002	0.999415
P200	P18	Paralemmin-2 OS=Homo sapiens GN=PALM2 PE=1 SV=3	0.0267467	0.990882
P200	P18	Zinc finger BED domain-containing protein 1 OS=Homo sapiens GN=ZBED1 PE=1 SV=1	0.00758557	0.978167
P18	P200	Sulfotransferase 1A2 OS=Homo sapiens GN=SULT1A2 PE=1 SV=2	-0.00341439	-0.987512
P200	P18	Dipeptidyl peptidase 4 OS=Homo sapiens GN=DPP4 PE=1 SV=2	0.071559	0.998524
P18	P200	Purine nucleoside phosphorylase OS=Homo sapiens GN=PNP PE=1 SV=2	-0.00566846	-0.995086
P200	P18	Protein ABHD14B OS=Homo sapiens GN=ABHD14B PE=1 SV=1	0.0270879	0.994075
P200	P18	Guanine nucleotide-binding protein G(I)/G(S)/G(T) subunit beta-2 OS=Homo sapiens GN=GNB2 PE=1 SV=3	0.0132282	0.941577
P200	P18	Sodium/glucose cotransporter 1 OS=Homo sapiens GN=SLC5A1 PE=1 SV=1	0.0152803	0.964976
P200	P18	4F2 cell-surface antigen heavy chain OS=Homo sapiens GN=SLC3A2 PE=1 SV=3	0.0262584	0.994474
P18	P200	Phosphate carrier protein, mitochondrial OS=Homo sapiens GN=SLC25A3 PE=1 SV=2	-0.00990643	-0.985443
P18	P200	Peptidyl-prolyl cis-trans isomerase A-like 4A OS=Homo sapiens GN=PPIAL4A PE=2 SV=1	-0.0210752	-0.984515
P200	P18	Macrophage-capping protein OS=Homo sapiens GN=CAPG PE=1 SV=2	0.00960401	0.988994

P200	P18	Ras-related protein Rab-7a OS=Homo sapiens GN=RAB7A PE=1 SV=1	0.0151536	0.993589
P200	P18	Beta-actin-like protein 2 OS=Homo sapiens GN=ACTBL2 PE=1 SV=2	0.0249975	0.978883
P200	P18	Disintegrin and metalloproteinase domain-containing protein 10 OS=Homo sapiens GN=ADAM10 PE=1 SV=1	0.00828697	0.909574
P200	P18	Prostate-specific antigen OS=Homo sapiens GN=KLK3 PE=1 SV=2	0.016361	0.975414
P200	P18	Mannan-binding lectin serine protease 2 OS=Homo sapiens GN=MASP2 PE=1 SV=4	0.0719671	0.999185
P200	P18	Pituitary tumor-transforming gene 1 protein-interacting protein OS=Homo sapiens GN=PTTG1IP PE=1 SV=1	0.0296841	0.95419
P200	P18	Electrogenic sodium bicarbonate cotransporter 1 OS=Homo sapiens GN=SLC4A4 PE=1 SV=1	0.0364444	0.980048
P200	P18	Calpain-7 OS=Homo sapiens GN=CAPN7 PE=1 SV=1	0.0232505	0.993405
P200	P18	Keratin, type I cytoskeletal 16 OS=Homo sapiens GN=KRT16 PE=1 SV=4	0.0280494	0.994863
P200	P18	Alpha-2-HS-glycoprotein OS=Homo sapiens GN=AHSG PE=1 SV=1	0.017551	0.992433
P200	P18	Palmitoyl-protein thioesterase 1 OS=Homo sapiens GN=PPT1 PE=1 SV=1	0.0118548	0.971523
P200	P18	Secreted and transmembrane protein 1 OS=Homo sapiens GN=SECTM1 PE=1 SV=2	0.0466123	0.98841
P18	P200	Carboxypeptidase E OS=Homo sapiens GN=CPE PE=1 SV=1	-0.0253776	-0.991968
P200	P18	Carboxymethylenebutenolidase homolog OS=Homo sapiens GN=CMBL PE=1 SV=1	0.0125996	0.997822
P18	P200	Hsc70-interacting protein OS=Homo sapiens GN=ST13 PE=1 SV=2	-0.00656364	-0.969805
P200	P18	Protein AMBP OS=Homo sapiens GN=AMBP PE=1 SV=1	0.0793425	0.996758
P200	P18	Proteasome subunit alpha type-7-like OS=Homo sapiens GN=PSMA8 PE=2 SV=3	0.00822444	0.981163
P200	P18	Zinc-alpha-2-glycoprotein OS=Homo sapiens GN=AZGP1 PE=1 SV=2	0.0209526	0.948597
P18	P200	Glutathione S-transferase A1 OS=Homo sapiens GN=GSTA1 PE=1 SV=3	-0.0129133	-0.988673
P200	P18	Prolactin-inducible protein OS=Homo sapiens GN=PIP PE=1 SV=1	0.0173955	0.992501
P18	P200	WAP four-disulfide core domain protein 2 OS=Homo sapiens GN=WFDC2 PE=1 SV=2	-0.00821208	-0.995172
P200	P18	Annexin A1 OS=Homo sapiens GN=ANXA1 PE=1 SV=2	0.0130384	0.91895
P18	P200	Src substrate cortactin OS=Homo sapiens GN=CTTN PE=1 SV=2	-0.0335653	-0.964795
P18	P200	Aldo-keto reductase family 1 member C3 OS=Homo sapiens GN=AKR1C3 PE=1 SV=4	-0.00667197	-0.962843
P18	P200	T-complex protein 1 subunit beta OS=Homo sapiens GN=CCT2 PE=1 SV=4	-0.00311411	-0.93832
P18	P200	Keratin, type I cytoskeletal 13 OS=Homo sapiens GN=KRT13 PE=1 SV=4	-0.0206433	-0.980976
P200	P18	Aquaporin-1 OS=Homo sapiens GN=AQP1 PE=1 SV=3	0.0330986	0.99846
P18	P200	Insulin-like growth factor-binding protein 2 OS=Homo sapiens GN=IGFBP2 PE=1 SV=2	-0.00622513	-0.974712
P18	P200	Keratin, type II cytoskeletal 80 OS=Homo sapiens GN=KRT80 PE=1 SV=2	-0.00527196	-0.95274
P200	P18	Transitional endoplasmic reticulum ATPase OS=Homo sapiens GN=VCP PE=1 SV=4	0.0119243	0.955597
P200	P18	Small integral membrane protein 24 OS=Homo sapiens GN=SMIM24 PE=2 SV=2	0.0126153	0.995687
P200	P18	Heat shock 70 kDa protein 1-like OS=Homo sapiens GN=HSPA1L PE=1 SV=2	0.0114105	0.971519
P200	P18	Epidermal growth factor receptor kinase substrate 8 OS=Homo sapiens GN=EPS8 PE=1 SV=1	0.0074306	0.922902
P18	P200	Doublecortin domain-containing protein 2 OS=Homo sapiens GN=DCDC2 PE=1 SV=2	-0.0162838	-0.969057
P200	P18	Angiotensin-converting enzyme 2 OS=Homo sapiens GN=ACE2 PE=1 SV=2	0.0242566	0.999528
P200	P18	Aminopeptidase N OS=Homo sapiens GN=ANPEP PE=1 SV=4	0.159937	0.999214
P200	P18	Uroplakin-1a OS=Homo sapiens GN=UPK1A PE=2 SV=1	0.0251676	0.978671

P18	P200	S-methylmethionine--homocysteine S-methyltransferase BHMT2 OS=Homo sapiens GN=BHMT2 PE=1 SV=1	-0.00455371	-0.931948
P200	P18	Hemoglobin subunit alpha OS=Homo sapiens GN=HBA1 PE=1 SV=2	0.00731551	0.973594
P200	P18	Annexin A7 OS=Homo sapiens GN=ANXA7 PE=1 SV=3	0.0202419	0.997661
P200	P18	Prostaglandin-H2 D-isomerase OS=Homo sapiens GN=PTGDS PE=1 SV=1	0.0413715	0.998714
P200	P18	Annexin A6 OS=Homo sapiens GN=ANXA6 PE=1 SV=3	0.0160982	0.912131
P200	P18	Neprilysin OS=Homo sapiens GN=MME PE=1 SV=2	0.0615054	0.992838
P18	P200	Unconventional myosin-VI OS=Homo sapiens GN=MYO6 PE=1 SV=4	-0.021349	-0.9208
P200	P18	Uroplakin-3a OS=Homo sapiens GN=UPK3A PE=1 SV=3	0.00902496	0.977852
P200	P18	Gamma-glutamyltranspeptidase 1 OS=Homo sapiens GN=GGT1 PE=1 SV=2	0.00566793	0.941884
P200	P18	Type 2 lactosamine alpha-2,3-sialyltransferase OS=Homo sapiens GN=ST3GAL6 PE=1 SV=1	0.018426	0.992024
P200	P18	Pyruvate kinase PKM OS=Homo sapiens GN=PKM PE=1 SV=4	0.0295031	0.935731
P18	P200	fMet-Leu-Phe receptor OS=Homo sapiens GN=FPR1 PE=1 SV=3	-0.00835799	-0.991966
P18	P200	Na(+)/H(+) exchange regulatory cofactor NHE-RF1 OS=Homo sapiens GN=SLC9A3R1 PE=1 SV=4	-0.0531738	-0.984863
P200	P18	Sodium-coupled monocarboxylate transporter 2 OS=Homo sapiens GN=SLC5A12 PE=2 SV=2	0.0154247	0.962749
P200	P18	N(G),N(G)-dimethylarginine dimethylaminohydrolase 2 OS=Homo sapiens GN=DDAH2 PE=1 SV=1	0.0162268	0.998769
P200	P18	Voltage-dependent anion-selective channel protein 2 OS=Homo sapiens GN=VDAC2 PE=1 SV=2	0.0114917	0.989464
P18	P200	Superoxide dismutase [Cu-Zn] OS=Homo sapiens GN=SOD1 PE=1 SV=2	-0.0130295	-0.920085
P18	P200	Cofilin-1 OS=Homo sapiens GN=CFL1 PE=1 SV=3	-0.0137485	-0.991209
P200	P18	Annexin A2 OS=Homo sapiens GN=ANXA2 PE=1 SV=2	0.0189853	0.991736
P200	P18	Annexin A5 OS=Homo sapiens GN=ANXA5 PE=1 SV=2	0.0188091	0.974331
P18	P200	Radixin OS=Homo sapiens GN=RDX PE=1 SV=1	-0.0074875	-0.973789
P200	P18	Ras-related protein Rab-10 OS=Homo sapiens GN=RAB10 PE=1 SV=1	0.0104716	0.986375
P200	P18	Alpha-1,6-mannosylglycoprotein 6-beta-N-acetylglucosaminyltransferase A OS=Homo sapiens GN=MGAT5 PE=2 SV=1	0.00646431	0.946986
P200	P18	Ketimine reductase mu-crystallin OS=Homo sapiens GN=CRYM PE=1 SV=1	0.0110998	0.982246
P200	P18	14-3-3 protein zeta/delta OS=Homo sapiens GN=YWHAZ PE=1 SV=1	0.0194105	0.9914
P18	P200	Cathepsin G OS=Homo sapiens GN=CTSG PE=1 SV=2	-0.0509977	-0.989126
P200	P18	Transketolase OS=Homo sapiens GN=TKT PE=1 SV=3	0.00898661	0.99937
P200	P18	Plasminogen OS=Homo sapiens GN=PLG PE=1 SV=2	0.0219994	0.969804
P200	P18	Retinal dehydrogenase 1 OS=Homo sapiens GN=ALDH1A1 PE=1 SV=2	0.0207607	0.989432
P18	P200	Retinol-binding protein 4 OS=Homo sapiens GN=RBP4 PE=1 SV=3	-0.0119673	-0.97878
P18	P200	Chloride intracellular channel protein 4 OS=Homo sapiens GN=CLIC4 PE=1 SV=4	-0.02183	-0.995161
P200	P18	Phospholipid hydroperoxide glutathione peroxidase, mitochondrial OS=Homo sapiens GN=GPX4 PE=1 SV=3	0.00774613	0.978176
P18	P200	Villin-1 OS=Homo sapiens GN=VIL1 PE=1 SV=4	-0.015823	-0.976316
P18	P200	Apolipoprotein A-I OS=Homo sapiens GN=APOA1 PE=1 SV=1	-0.0264564	-0.994525
P200	P18	EH domain-containing protein 4 OS=Homo sapiens GN=EHD4 PE=1 SV=1	0.0118406	0.980934
P200	P18	Protein tweety homolog 3 OS=Homo sapiens GN=TTYH3 PE=1 SV=3	0.0131757	0.969962
P200	P18	CD59 glycoprotein OS=Homo sapiens GN=CD59 PE=1 SV=1	0.0213445	0.906374
P200	P18	Fibrinogen beta chain OS=Homo sapiens GN=FGB PE=1 SV=2	0.00874747	0.994478

P18	P200	Plastin-1 OS=Homo sapiens GN=PLS1 PE=1 SV=2	-0.0023124	-0.918576
P200	P18	Desmoglein-1 OS=Homo sapiens GN=DSG1 PE=1 SV=2	0.00559341	0.992219
P200	P18	Glucose-6-phosphate isomerase OS=Homo sapiens GN=GPI PE=1 SV=4	0.0140775	0.976198
P200	P18	Fructose-bisphosphate aldolase A OS=Homo sapiens GN=ALDOA PE=1 SV=2	0.0116353	0.989914
P18	P200	Uromodulin OS=Homo sapiens GN=UMOD PE=1 SV=1	-0.177385	-0.99518
P200	P18	L-xylulose reductase OS=Homo sapiens GN=DCXR PE=1 SV=2	0.00933627	0.983738
P18	P200	Actin, cytoplasmic 1 OS=Homo sapiens GN=ACTB PE=1 SV=1	-0.0939253	-0.983303
P18	P200	Apolipoprotein A-IV OS=Homo sapiens GN=APOA4 PE=1 SV=3	-0.0255959	-0.970023
P200	P18	Actin-related protein 2/3 complex subunit 2 OS=Homo sapiens GN=ARPC2 PE=1 SV=1	0.0169374	0.919449
P18	P200	Tubulin beta chain OS=Homo sapiens GN=TUBB PE=1 SV=2	-0.00619707	-0.941706
P18	P200	Tubulin beta-3 chain OS=Homo sapiens GN=TUBB3 PE=1 SV=2	-0.00586377	-0.940259
P18	P200	Malate dehydrogenase, cytoplasmic OS=Homo sapiens GN=MDH1 PE=1 SV=4	-0.0154978	-0.983061
P18	P200	Na(+)/H(+) exchange regulatory cofactor NHE-RF3 OS=Homo sapiens GN=PDZK1 PE=1 SV=2	-0.0412959	-0.985348
P18	P200	Quinone oxidoreductase OS=Homo sapiens GN=CRYZ PE=1 SV=1	-0.00344869	-0.900317
P200	P18	Brain-specific angiogenesis inhibitor 1-associated protein 2 OS=Homo sapiens GN=BAIAP2 PE=1 SV=1	0.0105435	0.971745
P200	P18	Dipeptidase 1 OS=Homo sapiens GN=DPEP1 PE=1 SV=3	0.0648745	0.99099
P200	P18	Gelsolin OS=Homo sapiens GN=GSN PE=1 SV=1	0.0302858	0.988085
P200	P18	Dermcidin OS=Homo sapiens GN=DCD PE=1 SV=2	0.0250014	0.980782
P200	P18	Cathepsin Z OS=Homo sapiens GN=CTSZ PE=1 SV=1	0.0126514	0.94495
P18	P200	Aspartate aminotransferase, cytoplasmic OS=Homo sapiens GN=GOT1 PE=1 SV=3	-0.00631861	-0.966952
P18	P200	Claudin-16 OS=Homo sapiens GN=CLDN16 PE=1 SV=1	-0.00584719	-0.908886
P18	P200	Fatty acid-binding protein, liver OS=Homo sapiens GN=FABP1 PE=1 SV=1	-0.0165542	-0.953229
P200	P18	Keratin, type II cytoskeletal 1 OS=Homo sapiens GN=KRT1 PE=1 SV=6	0.0889889	0.977723
P18	P200	Osteopontin OS=Homo sapiens GN=SPP1 PE=1 SV=1	-0.00746237	-0.90027
P200	P18	V-type proton ATPase catalytic subunit A OS=Homo sapiens GN=ATP6V1A PE=1 SV=2	0.016334	0.984022
P200	P18	Keratin, type I cytoskeletal 10 OS=Homo sapiens GN=KRT10 PE=1 SV=6	0.0586634	0.915438
P18	P200	Gap junction alpha-1 protein OS=Homo sapiens GN=GJA1 PE=1 SV=2	-0.00425051	-0.950256
P18	P200	Voltage-dependent anion-selective channel protein 1 OS=Homo sapiens GN=VDAC1 PE=1 SV=2	-0.019425	-0.983882
P200	P18	Uroplakin-2 OS=Homo sapiens GN=UPK2 PE=1 SV=2	0.0151759	0.919957
P200	P18	Aflatoxin B1 aldehyde reductase member 3 OS=Homo sapiens GN=AKR7A3 PE=1 SV=2	0.0119828	0.93696
P18	P200	Nicotinamide phosphoribosyltransferase OS=Homo sapiens GN=NAMPT PE=1 SV=1	-0.00230504	-0.9608
P200	P18	Lambda-crystallin homolog OS=Homo sapiens GN=CRYL1 PE=1 SV=3	0.0108364	0.986094
P200	P18	Keratin, type II cytoskeletal 2 oral OS=Homo sapiens GN=KRT76 PE=1 SV=2	0.0114675	0.968403
P18	P200	Triosephosphate isomerase OS=Homo sapiens GN=TPI1 PE=1 SV=3	-0.0182586	-0.954163
P18	P200	Calmodulin OS=Homo sapiens GN=CALM1 PE=1 SV=2	-0.0137222	-0.939388
P200	P18	Moesin OS=Homo sapiens GN=MSN PE=1 SV=3	0.0181273	0.959497
P18	P200	Aminoacylase-1 OS=Homo sapiens GN=ACY1 PE=1 SV=1	-0.0274601	-0.965669
P18	P200	Glycerol-3-phosphate dehydrogenase [NAD(+)], cytoplasmic OS=Homo sapiens GN=GPD1 PE=1 SV=4	-0.00718951	-0.972866
P200	P18	ADP-ribosylation factor 1 OS=Homo sapiens GN=ARF1 PE=1 SV=2	0.0134561	0.957347

P200	P18	Aromatic-L-amino-acid decarboxylase OS=Homo sapiens GN=DDC PE=1 SV=2	0.00857648	0.988048
P200	P18	Formimidoyltransferase-cyclodeaminase OS=Homo sapiens GN=FTCD PE=1 SV=2	0.00689556	0.930641
P200	P18	Cell division control protein 42 homolog OS=Homo sapiens GN=CDC42 PE=1 SV=2	0.00928424	0.914252
P200	P18	Glyceraldehyde-3-phosphate dehydrogenase OS=Homo sapiens GN=GAPDH PE=1 SV=3	0.0377487	0.934878
P18	P200	Keratin, type I cytoskeletal 24 OS=Homo sapiens GN=KRT24 PE=1 SV=1	-0.00911415	-0.973
P18	P200	Phosphoglycerate kinase 1 OS=Homo sapiens GN=PGK1 PE=1 SV=3	-0.0176474	-0.905495
P18	P200	L-lactate dehydrogenase B chain OS=Homo sapiens GN=LDHB PE=1 SV=2	-0.0148779	-0.923777
P18	P200	Phosphoenolpyruvate carboxykinase, cytosolic [GTP] OS=Homo sapiens GN=PCK1 PE=1 SV=3	-0.0116297	-0.938965
P200	P18	Histone H4 OS=Homo sapiens GN=HIST1H4A PE=1 SV=2	0.004287	0.960698
P200	P18	Keratin, type II cytoskeletal 2 epidermal OS=Homo sapiens GN=KRT2 PE=1 SV=2	0.0257826	0.94864
P18	P200	Fructose-bisphosphate aldolase B OS=Homo sapiens GN=ALDOB PE=1 SV=2	-0.0301774	-0.957998
