# Supplementary material for: Urinary extracellular vesicles carry multiple activators and regulators of coagulation
Source: Front Cell Dev Biol. 2022 Sep 7;10:967482. doi: 10.3389/fcell.2022.967482 (PMC9489905; doi:10.3389/fcell.2022.967482)
Supplement: Supplementary file 2 [file DataSheet1.PDF]

Urinary extracellular vesicles carry multiple activators and regulators of coagulation

Mayank Saraswat[1, *], Beata Przybyla[2], Sakari Joenvaara[1, 3], Tiialotta Tohmola[4], Tomas Strandin[5], Maija Puhka[6], Annukka Jouppila[7], Riitta Lassila[8, #], Risto Renkonen[1, 3, #]

Supplementary Methods:

Western blotting

Briefly, total protein concentration of EVs (P18 and P200 pellets) was determined using the Pierce BCA assay kit by microBCA procedure (Thermo Fisher, CA, USA) according to the manufacturer's instructions. Fifty µg of total protein was dried in speed vacuum and dissolved in 2x laemmli buffer (Bio Rad, CA, USA). The sample was subsequently boiled with or without 50mM DTT (final concentration) and loaded into the 4–15% TGX precast gels (Bio-Rad). The gels were electroblotted to iBlot® 2 nitrocellulose regular stacks (Invitrogen) for 7 min with iBlot® 2 gel transfer device. The membranes were blocked in 1:1 odyssey blocking buffer: PBS for 1 h at room temperature (RT). Primary antibody solution made in 1:1 Odyssey blocking buffer: PBS (1:1000) was incubated with the membrane for overnight at 4°C with end to end rotation. The next day, membrane was washed six times with PBS-Tween (Tween-20 at concentration of 0.1%) and incubated with Li-Cor secondary antibodies (680 or 800nm dye conjugated, Li-Cor, NE, USA) for 1 h at RT (1:10,000, 0.01% SDS in blocking buffer). The membranes were again washed five times with PBS-Tween and once with PBS. The membranes were then scanned with Odyssey Infrared image scanner (Li-Cor). No blot was cropped and the subsequent blots are presented after converted to black and white by Odyssey software. FVIII blot was a little overexposed due to the low protein signal, compatible with the low concentration of FVIII overall in the body. No other

manipulation was involved. Primary antibodies used were as follows: Anti- Protein C (PA5-28321, Thermo Fisher), CD142/TF (PA1-43058, Thermo Fisher), AT (PA1-43054, Thermo Fisher), TFPI (PA5-19809), FV (sc-13512, Santa Cruz Biotechnology), FVII (sc-81679, Santa Cruz Biotechnology), FVIII (PA1-43045, Thermo Fisher), FX (MA1-43013, Thermo Fisher), Prothrombin (05-879, Merck Millipore). All secondary antibodies were from Li-Cor (NE, USA).

Transmission electron microscopy

Briefly, EV samples were loaded on grids, fixed with 2 % PFA, stained with 2 % neutral uranyl acetate and embedded in methyl cellulose uranyl acetate mixture (1.8/0.4%). The samples were viewed with transmission EM using Tecnai 12 (FEI Company, Eindhoven, The Netherlands) operating at 80 kV and imaged with Gatan Orius SC 1000B CCD-camera (Gatan Inc. USA).

Calibrated Automated Thrombogram

Plasma (80 µL) as such or diluted with P18 and P200 (5:4 ratio, plasma:vesicles at initial vesicle concentration of 50 µg/mL) or PBS (as control in the same ratio) was supplemented either with PPP reagent Low® (20 µL) including 1 pM TF and 4 µM phospholipids (PLs) or with the calibrator (Thrombin calibrator®) of known thrombin activity. TG was initiated by re-calcification with FluCa® (20 µL) including the fluorogenic substrate for thrombin. TG was monitored with Thrombinoscope software (Synapse BV, The Netherlands) during 60 min in all assays, and 120 min in case of assaying FVIII with CAT® (because TG came down to baseline after 90 minutes). CAT® reagents were from Diagnostica Stago, France.

Trypsin digestion and Proteomic analysis

Briefly, thirty µg of P18 and P200 total proteins, dried in separate tubes, were dissolved in 0.5% Rapigest SF in 50 mM ammonium bicarbonate (Waters Corp., Milford, MA, USA) and boiled for 5 min. Thereafter, they were digested according to manufacturer's instructions (Waters). Samples were cleaned with C18 micro-columns (Pierce, Thermo Fisher) and after drying, dissolved in 0.1% formic acid supplemented with 12.5 fmol/µL of Hi3 peptide mixture (Waters). Mass spectrometry analysis was performed as described previously(Saraswat et al., 2016). Briefly, TRIZAIC nanoTile 85µm x 100 mm HSS-T3u wTRAP was used as separating device prior to mass spectrometer. The analytical gradient used for UPLC is given in the Table below:

Minutes	Flow rate (µL/min)	%B
Initial	0.550	1
4	0.550	1
7	0.550	5
17	0.550	7.6
26	0.550	10.3
35	0.550	13.1
43	0.550	16.1
51	0.550	19.2
59	0.550	22.4
67	0.550	25.7
75	0.550	29.1
83	0.550	32.6
90	0.550	36.2
97	0.550	40
98	0.550	90
100.5	0.800	90
102	0.800	1
102.5	0.550	1
117	0.450	1
118	0.450	1

The raw files were imported to Progenesis QI for proteomics (Version V2, Nonlinear Dynamics, Newcastle, UK) for relative quantification and identification of proteins. Post-acquisition mass correction was performed during the file import using lock mass of 785.8426 m/z (2+ Glu1-Fibrinopeptide B). The default parameters for peak picking and alignment were used and proteins were identified by Protein Lynx Global Server within Progenesis QI for proteomics. Orthogonal

projections to latent structures-discriminant analysis (OPLS-DA) was performed in EZInfo 3.0 and visualized by S-Plot.
